# Arylazo Sulfones as 1,3-Dipole Acceptors in the (Photo)-Micellar van Leusen Triazole Synthesis

**DOI:** 10.1021/acsorginorgau.5c00080

**Published:** 2025-09-15

**Authors:** Carmine Volpe, Luca Nicchio, Federica Santoro, Teresa Silvestri, Anna Di Porzio, Camilla Russo, Fabiana Quaglia, Antonio Randazzo, Alfonso Carotenuto, Diego Brancaccio, Stefano Protti, Mariateresa Giustiniano

**Affiliations:** † Department of Pharmacy, 9307 University of Naples Federico II, via D. Montesano 49, 80131 Napoli, Italy; ‡ PhotoGreen Lab, Department of Chemistry, 19001 University of Pavia, Viale Taramelli 12, 27100 Pavia, Italy

**Keywords:** arylazo sulfones, isonitriles, micellar catalysis, 1,2,4-triazoles, van Leusen reaction

## Abstract

Arylazo sulfones represent a valuable class of organic compounds, which can be exploited as safer and bench-stable analogues of diazonium salts. Notwithstanding their significant applications in light-triggered reactions as precursors of either diazenyl or aryl radicals, their reactivity as 1,3-dipole acceptors has been poorly investigated. The present manuscript addresses the study of a [3 + 2] cycloaddition reaction between arylazo sulfones and α-acidic isonitriles such as TosMIC and related analogues. Reaction conditions have been optimized in a micellar medium, namely CTAC 2% aq solution, in order to afford sustainable synthetic access to 1,2,4-triazole derivatives, a relevant scaffold in medicinal and material chemistry. The substrate scope has been showcased with 24 examples (24–90% yield) with good functional group tolerability. Worthy of note, solution NMR techniques have been applied to characterize the reaction at the molecular level, providing a groundwork to drive a rational choice of the micellar medium on the basis of the substrates’ chemical nature. Moreover, the recyclability of the micellar medium was investigated via dynamic light scattering (DLS) analyses.

## Graphical abstract

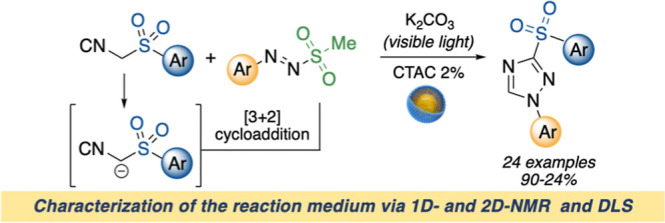

## Introduction

Arylazo sulfones (ArN_2_SO_2_R) are bench-stable and safer analogues of diazonium salts, sharing with the latter a rich and fascinating chemistry.^1–3^ Indeed, the presence of the dyedauxiliary group –N_2_SO_2_R enables the direct harvesting of visible light, and the so-generated excited state eventually results in a homolytic fragmentation, affording the diazenyl radical (ArN_2_^•^)/sulfonyl (RSO_2_^•^) radical pair (Scheme 1a). The former can either be trapped by an electron-rich species^4^ or undergo a loss of molecular nitrogen (N_2_) and release an aryl radical (Ar^•^), in turn suitable for addition to π-systems such as alkenes and heterocycles and to isonitriles.^1–3^ The same intermediate can also act as a hydrogen atom transfer (HAT) agent to promote the formation of alkyl radicals.^5^ More recently, however, the reactivity of arylazo sulfones as electrophiles was explored by Jiangand and co-workers, who proposed a base-mediated approach for the preparation of asymmetric diazoarenes in alcohols.^6^ Intrigued by the multifaceted reactivity of arylazo sulfones and by the possibility of developing synthetic protocols under environmentally benign reaction conditions (water-based reaction medium, light as a renewable energy source, absence of catalysts/additives), we wondered to enlarge the applications of such azocompounds by focusing on their use as precursors of nitrogen-based heterocycles. Thus, as a continuation of our research interests in isonitrile chemistry,^4,5^ we focused the attention on the van Leusen triazole synthesis typically involving electron-poor diazonium salts and *p*-toluenesulfonylmethylisocyanide (TosMIC) (Scheme 1b).^7^ This reaction was reported early by van Leusen in 1976 and emerged from the exploitation of both reactive sites of TosMIC—i.e., the activated methylene and the isonitrile functional group—in a [3 + 2] cycloaddition with poorly stable diazonium salts to afford 1,2,4-triazoles as a mixture of regioisomers (Scheme 1b). Notably, 1,2,4-triazoles are endowed with a plethora of biological activities, including antibacterial, antiviral, anticoagulant, anti-inflammatory, and anticancer.^8–11^ Accordingly, they represent an emerging privileged scaffold in medicinal chemistry, featuring relevant physicochemical properties such as dipolar character, rigidity, and hydrogen bonding ability, along with improved stability to metabolic degradation.^12–14^

**Scheme 1:**
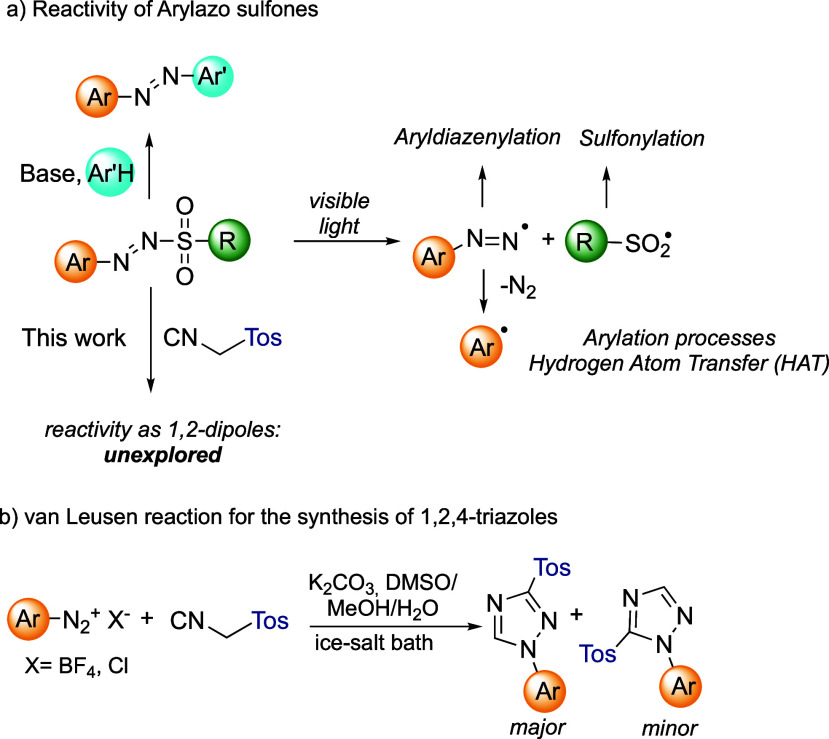
a) General Reactivity of Arylazo Sulfones and (b) van Leusen Approach for the Synthesis of 1,2,4-Triazoles

Recently, they have also been reported as amide bioisosters.^15^ Furthermore, these heterocycles have a prominent role in the development of functional and energetic materials, polymers, metal–organic frameworks (MOFs), corrosion inhibitors, and sensors.^8^ Despite the huge potential of the van Leusen triazole synthesis, the reaction conditions developed at that time led to a poor substrate scope (7 examples, limited to electron-rich substrates, 0–94% yield).^7^ Alternatively, according to more recent methods, 1,3-disubstituted 1,2,4-triazoles can be accessed starting either from acylhydrazides and formamidinium acetate or hydrazinoyl chlorides and *N*-methylimidazole.^16,17^ However, these reactions require the use of metal catalysts, additives, halogenated solvents, and heating over long reaction times. In rare cases, light-mediated approaches have been proposed. Considering the wide range of industrial applications of 1,2,4-triazoles, we engaged in the development of a new synthetic method featuring mild and green reaction conditions, as well as a good substrate scope and functional group orthogonality.

## Results and Discussion

### Optimization of Reaction Conditions and Investigation of the Substrate Scope

In light of our interests in developing (photo)­micellar catalyzed reactions, we set out conditions featuring the use of a water-based reaction medium. More in detail, given that, in the presence of a base, TosMIC would form a carbanion able to react as a 1,3-dipole, and considering the feasibility of a reverse polarity principle,^18^ we observed that good yields were obtained by using a positively charged micelle-forming surfactant, namely a CTAC 2% aqueous solution (0.1 M) and K_2_CO_3_ (2 equiv) as a base (61% yield for the test substrates as in entry 1, Table 1). Light irradiation has a slightly beneficial effect, boosting the yield to 71% (entry 2). The same trend, i.e., 51% yield in the dark versus 61% yield under blue light irradiation, arose from using pure water as a reaction medium (entries 3–4), thus proving that both light and micellar medium are favorable. Negatively charged micelles such as SDS and neutral ones, such as TPGS-750M, led to poorer outcomes (56 and 46% yields, respectively, entries 5–6). Similar results were also obtained (1) with a milder base such as NaHCO_3_ (2 equiv), independently from the reaction solvents (either pure water or micellar media, entries 7–13), (2) by increasing the equivalents of TosMIC (entries 9–14), and (3) in a mixture of MeCN/H_2_O 9:1 (entry 13). Not surprisingly, the presence of a base is required for the formation of the product (entry 14). A 64% of **3a** was isolated when the reaction mixture was heated at 50 °C (entry 15).

**Table 1:**
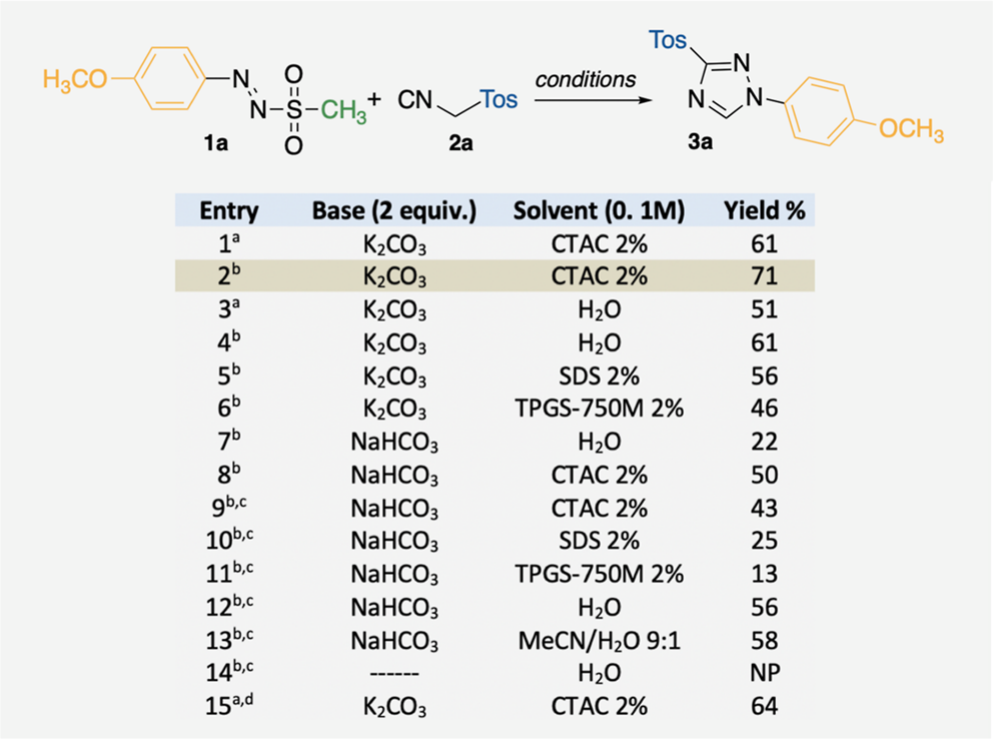
Standard Conditions for Test Substrates and Relative Deviations^a^

a **1a**, 0.03 mmol, 1 equiv.; **2a**, 0.06 mmol, 2 equiv. ^a^In the dark. ^b^Blue LEDs 30 W. ^c^5 equiv of TosMIC. ^d^In the dark at 50 °C. ^e^Reactions run for 20 h, at room temperature, with NMR yields except for entry 1.

To investigate the substrate scope and the functional group tolerance of the photomicellar van Leusen triazole synthesis, a library of 24 derivatives was prepared (**3b**–**x**, Figure 1). Good to excellent yields were obtained with different isocyanomethyl sulfonyl arenes, featuring naphthyl (**3b** and **3c**, 75 and 63% yields, respectively), 4-chloro (**3d**–**3g**, in the 90–62% yield range), and 4-bromo (**3g**–**3i**, 87–72% yields) aryl moieties. Interestingly, methylisocyanoacetate was able to react as a 1,3-dipole comparably to isocyanomethyl sulfonyl arenes, as shown for product **3j** (64% yield). A broader survey of the arylazo sulfone moiety was performed by keeping TosMIC as the 1,3-dipole (**3k**–**3x**, Figure 1). The general conditions proved to be suitable for functional groups such as dimethylamino (**3k**, 79%), amide (**3l**, 41%), sulfide (**3m**, 62%), halogens (**3n**–**3r**, 44–80%), and trifluoromethoxy groups (**3r**, 34%). Unsubstituted phenylazo sulfone afforded the product **3s** in 40% yield, while electron-donor substituents such as in products **3t** and **3u** led to an increased 53% yield for both. As for substrates bearing electron-withdrawing functional groups, the reaction performed under light irradiation led to a complex mixture of adducts, while under dark conditions the regioselectivity resulted in improved yields, affording the corresponding 1-aryl monosubstituted triazole **3v** (83% yield, Figure 1) or a mixture of mono- and disubstituted products **3w** and **3x** (50% each by NMR yield, 30% and 10% yields, respectively, by PLC). It is worth noting that under the classical van Leusen conditions, the use of *p*-nitrophenyl diazonium tetrafluoroborate led to a benzamide derivative without any traces of triazole isomers detected.^7^

**Figure 1:**
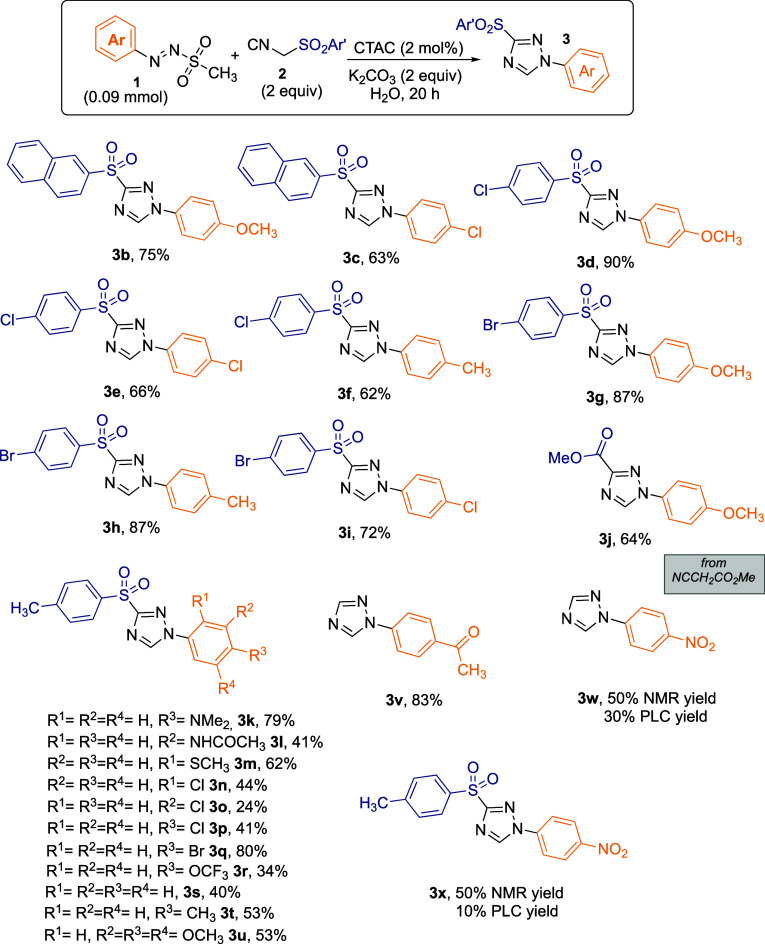
Reaction scope (products **3k**–**3u** were obtained under irradiation with 30 W blue LEDs; products **3v**–**3x** obtained under dark conditions).

### Mechanistic Studies

A working mechanistic hypothesis is depicted in Scheme 2. Based on the reaction conditions, it is likely to suppose the formation of a TosMIC carbanion **2a** ^**–**^, which can act as a 1,3-dipole and trigger a formal [3 + 2]-cycloaddition with the arylazo sulfone **1** to give intermediate **A** (Scheme 2a). The latter undergoes protonation by water to give **B**, followed by aromatization to triazole **3** with concomitant loss of methylsulfinate CH_3_SO^–^ (Scheme 2a). This hypothesis was corroborated by the obtainment of a deuterium-labeled triazole **3a–D** with 80% yield and 99.9% of deuterium incorporation (Scheme 2b) when the reaction was performed in the presence of deuterium oxide (D_2_O). As apparent in Table 1, in some cases, light can slightly influence the reaction. We suggested that, similar to what has been recently observed for arylazo sulfonates,^19^ irradiation of arylazo sulfones in aqueous media can result in the heterolytic cleavage of an N–S bond to release a diazonium salt (which was also detected by performing the reaction in the presence of indole, see Supporting Information for further details), which generates **3** via a classic van Leusen reaction (Scheme 2c).

**Scheme 2:**
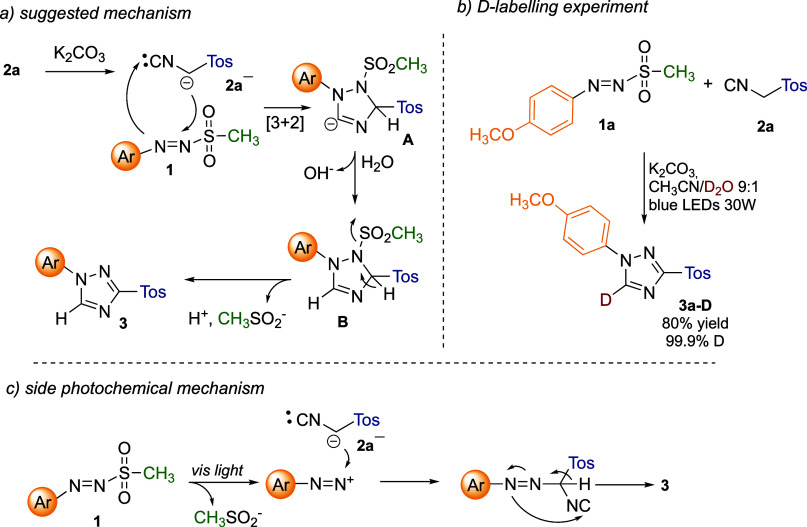
Mechanistic Hypotheses and Experimental Studies

On the other hand, the formation of radical species via homolytic dissociation of **1a** was excluded on the basis of a test reaction performed under the standard conditions and in the presence of the radical quencher TEMPO (2,2,6,6-Tetramethylpiperidine 1-oxyl, 1 equiv) (see Supporting Information 156 for further details). The expected triazole **3a** was obtained without any decrease in the yield (namely, 70%), thus proving that radical species, such as a diazenyl radical, are not involved in triazole formation. While it is possible to indicate path a as the main player in dark reaction conditions, it is more difficult to establish which between path a and path c is the predominant mechanism when the reaction is performed under visible light irradiation, as well as whether the two pathways would contribute to a different extent based on the arylazosulfone substitution pattern (electron-rich versus electron-poor substrates).

### NMR Characterization of the Reaction Environment

Photomicellar catalysis has been becoming a more and more attractive research field thanks to the ability to combine the innate green features of both water-based media and light-triggered chemical processes.^20^ Nevertheless, the photomicellar reaction environment is usually poorly investigated at a molecular level, despite its importance in providing a rational groundwork for the proper choice of micelle-forming surfactants (neutral and either positively or negatively charged). The interactions occurring between the reagents and the employed micellar systems, CTAC, SDS, and TPGS-750-M 2% solutions, were investigated at the molecular level via solution NMR techniques.^21^

1D ^1^H NMR spectra of TosMIC (**2a**) and arylazo sulfone **1a** were acquired in pure water and in the presence of micelles (Supporting Information, Figures S1 and S2); for TosMIC (**2a**), the same spectra in the presence of carbonate K_2_CO_3_ were also registered (Figure 2).

**Figure 2:**
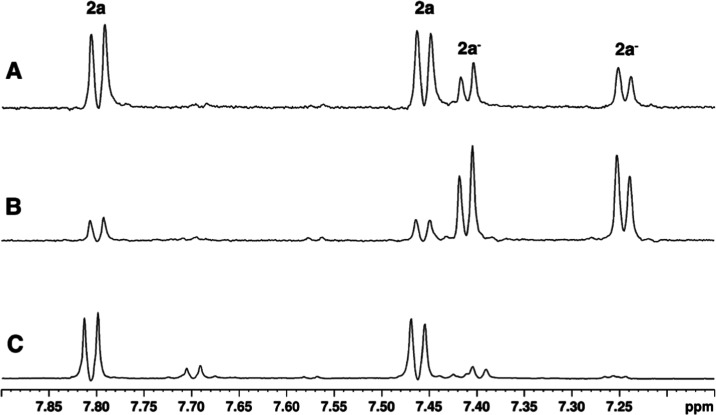
1D ^1^H NMR spectra of TosMIC **2a** (0.12 mmol) in pure water (0.1 M) in the absence (C) and in the presence of K_2_CO_3_ (1 equiv), freshly prepared (A) and after 24 h (B). Signals attributed to the neutral form (**2a**) and the dissociated form (**2a** ^**–**^) are labeled accordingly in the spectra.

TosMIC (**2a**) in a carbonate solution showed two sets of signals (Figure 2). One system was almost perfectly overlapping with that observable in pure water and tended to reduce its intensity over time; conversely, the intensity of the other system tended to increase over time and an equilibrium was reached after about 24 h. This could be interpreted as a slow and partial dissociation of the TosMIC (**2a**) to form the carbanion **2a** ^**–**^ with a final ratio between the neutral and dissociated form of 1:4 from the NMR peaks’ integration under these experimental conditions.

In a 0.1 M CTAC 2% aq solution, TosMIC also slowly dissociated to form the carbanion **2a** ^**–**^ (Supporting Information, Figure S1). Noteworthy, unlike what happens in pure water, this conversion was complete in CTAC micelles. The localization of TosMIC (**2a**) relative to the surface and interior of CTAC micelles was studied using 2D NOESY spectra (Figure 3). NOESY displayed clear dipolar couplings between TosMIC (**2a**) and the micelle protons, both methylene and ammonium methyl groups, indicating that TosMIC (**2a**) interacted with the CTAC micelles mainly at the surface.

**Figure 3:**
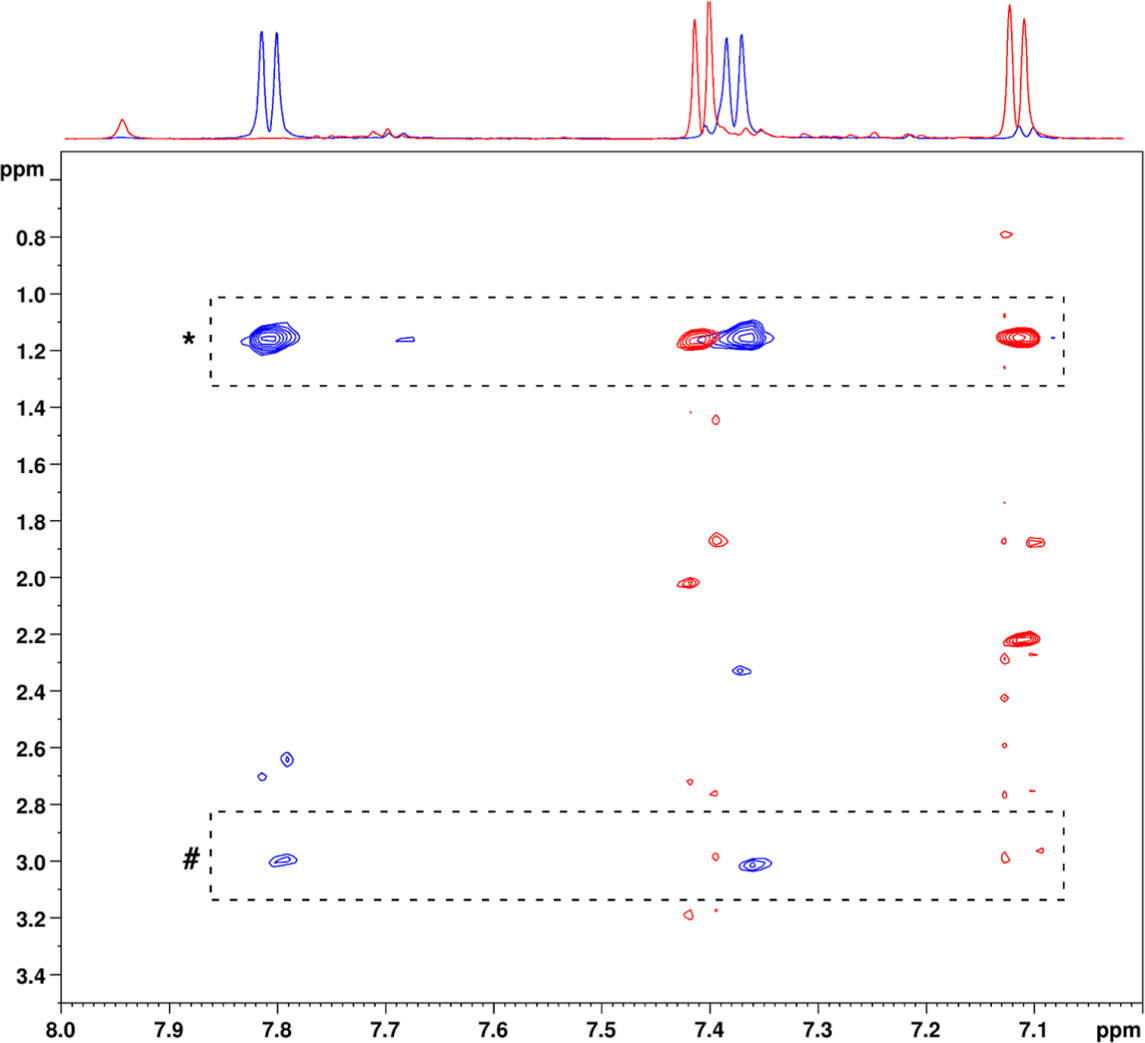
2D ^1^H–^1^H NOESY spectra of TosMIC (0.12 mmol) in CTAC 2% aq. solution (0.1 M) and K_2_CO_3_ (1 equiv) freshly prepared (blue) and after 24 h (red). NOE cross-peaks between TosMIC (**2a**) and CTAC micelle protons are highlighted with dashed boxes; in particular, those with methylene groups are marked with (*), and those with ammonium methyl groups with (#).

As for the arylazo sulfone **1a**, its spectra in pure water indicated the presence of three signal systems compatible with the *trans* (likely the predominant one), the *cis* form, and the diazonium salt, the latter forming from **1a** upon heterolytic cleavage (Supporting Information, Figure S2). In the CTAC 2% aq solution, the predominant system had no NOEs contacts with the micelles, and the chemical shifts of **1a** were superimposable with those of the spectrum registered in pure water (Figure 4). In contrast, the lower-intensity systems showed NOE interactions with the CTAC micelles. In particular, NOEs are observed between the aromatic proton signals and the N–CH_2_ groups (3.2 ppm) on the polar surface. To further determine the relative position of the arylazo sulfone **1a** and micelles, paramagnetic Mn^2+^ was also used (Supporting Information, Figure S3). This paramagnetic probe was expected to cause broadening of the NMR signals and a decrease in resonance intensities for a molecule outside the micelle in the bulk water. Addition of Mn^2+^ to a 0.1 M CTAC 2% aq. solution of compound **1a** broadened the signals belonging to all three systems, but only to a limited extent. This confirmed that the minor components are engaged in contacts with the micelles, but it also disclosed that the main system of arylazo sulfone **1a** is partially protected from the bulk water and localized at the Stern layer.

**Figure 4:**
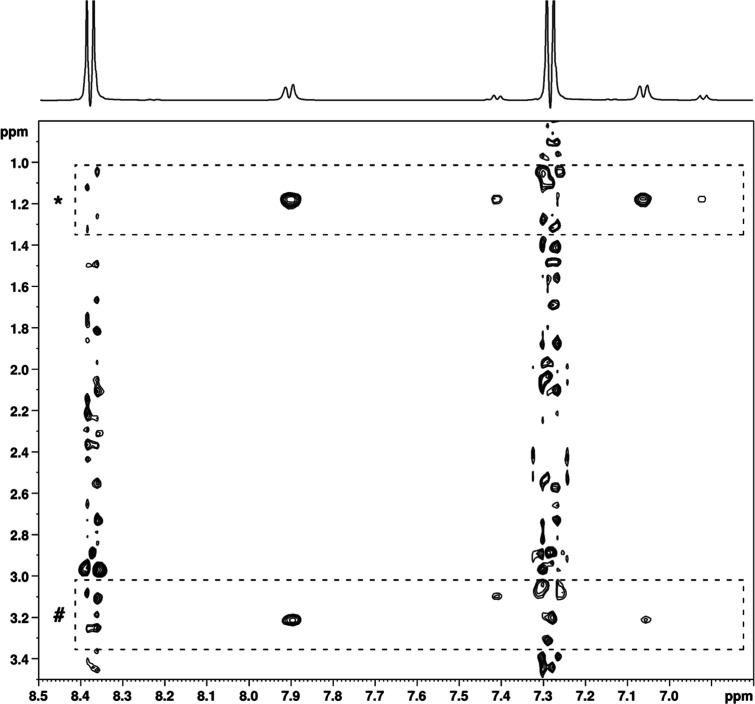
2D ^1^H–^1^H NOESY spectrum of arylazo sulfone **1a** (0.06 mmol) in CTAC 2% aq. Solution (0.1 M). NOE cross-peaks between **1a** and CTAC micelle protons are highlighted with dashed boxes; in particular, those with methylene groups are marked with (*), and those with ammonium methyl groups with (#).

To summarize, CTAC micelles enhanced the deprotonation of TosMIC to form the reactive species **2a** ^**–**^ probably via ionic interactions, and concentrated the same **2a** ^**–**^ and the other reaction component, the arylazo sulfone **1a** (*trans*, *cis*, and diazonium forms), in the micelle Stern layer, thus improving the reaction yield compared to pure water.

In SDS 2% aq solution, TosMIC (**2a**) showed a 1D ^1^H NMR spectrum almost perfectly superimposable with that observed in water (Supporting Information, Figure S1), and no correlation signals were observed in the NOESY spectrum between TosMIC (**2a**) and SDS micelle signals, indicating the absence of interaction of the TosMIC (**2a**) and micelles (data not shown). Moreover, in SDS micelles, signals assignable to the hydrated form of TosMIC (formamide derivative) are clearly observable. As a result, the proportion of the reactive dissociated **2a** ^**–**^ relative to the neutral **2a** was lower in the presence of SDS compared to that of water, accounting for 58% in SDS versus 80% in water. Considering the arylazo sulfone **1a**, the NOESY spectrum did not show any interaction with the SDS micelles, and the addition of Mn^2+^ had the effect of remarkably broadening all the signals, indicating that the arylazo sulfone **1a** (in all its forms) has weak, if any, interactions with the SDS micelles (Supporting Information, Figure S4). As a consequence of the almost absent interactions between the SDS micelles and both reagents **1a** and **2a**, the reaction yield is very similar to that observed in water. The minimal reduction in the reaction yield can derive from a reduced percentage of reactive dissociated TosMIC (**2a** ^–^) observed in the presence of this micelle.

In TPGS-750-M 2% aq solution, 1D ^1^H NMR signals deriving from the undissociated form of TosMIC (**2a**) had NOE correlations with the micelles, including the methyl signals of the micelle core. Moreover, these signals were significantly broader compared to the corresponding signals in the proton 1D ^1^H NMR spectrum registered in pure water. In contrast, the signals from the dissociated form (**2a** ^**–**^) had no NOEs with the micelles and resonated at the same chemical shifts as the corresponding signals in pure water (Supporting Information, Figures S1 and S5). Hence, the neutral **2a** was deeply inserted into the micelle, while the anionic **2a** ^**–**^ did not interact with the same TPGS-750-M micelles. Finally, the percentage of reactive **2a** ^**–**^ remained as low as 20% after 24 h.

The main system (*trans* isomer) of arylazo sulfone **1a** weakly interacted with the external PEG region of the TPGS-750-M micelles as deduced from the NOEs in the NOESY spectrum and from the virtually identical chemical shift of the signals in pure water and TPGS-750-M micelles. In contrast, the species belonging to minor systems (*cis* isomer and diazonium ion) more deeply interacted with the TPGS-750-M, as deduced from the NOE contacts with more internal micelle signals (Supporting Information, Figure S6). Minimal line broadening observed for all signals upon the addition of Mn^2+^ confirms that compound **1a** was engaged in the association with the micelles to a certain extent (Supporting Information, Figure S7).

The seizure of the neutral TosMIC (**2a**) within the TPGS-750-M micelles, the apparent decrease in its dissociation tendency in the presence of these micelles, and the low ability of the same micelles to concentrate the arylazo sulfone **1a** in the micellar core caused the observed decrease of the reaction yield in the presence of TPGS-750-M.

### Recycling of the Micellar Reaction Medium

Recycling of the micellar aqueous mixture could, in theory, enable a consistent E-factor decrease, a green chemistry metric that is key on a manufacturing scale. In order to investigate whether this could be feasible, after running a first reaction with arylazo sulfone **1a** and TosMIC (**2a**), the product **3a** was extracted with ethyl acetate (70% yield, reaction scale: **1a** 0.09 mmol, ethyl acetate: 0.9 mL × 3, Figure 5) directly in the reaction flask, and the starting materials **1a** and **2a** were added again to the micellar mixtures. While the first recycle afforded product **3a** in a 65% yield, the attempt of a second recycle (third reaction run) led to a dramatic drop in the yield of **3a** (20%, Figure 5).

**Figure 5:**
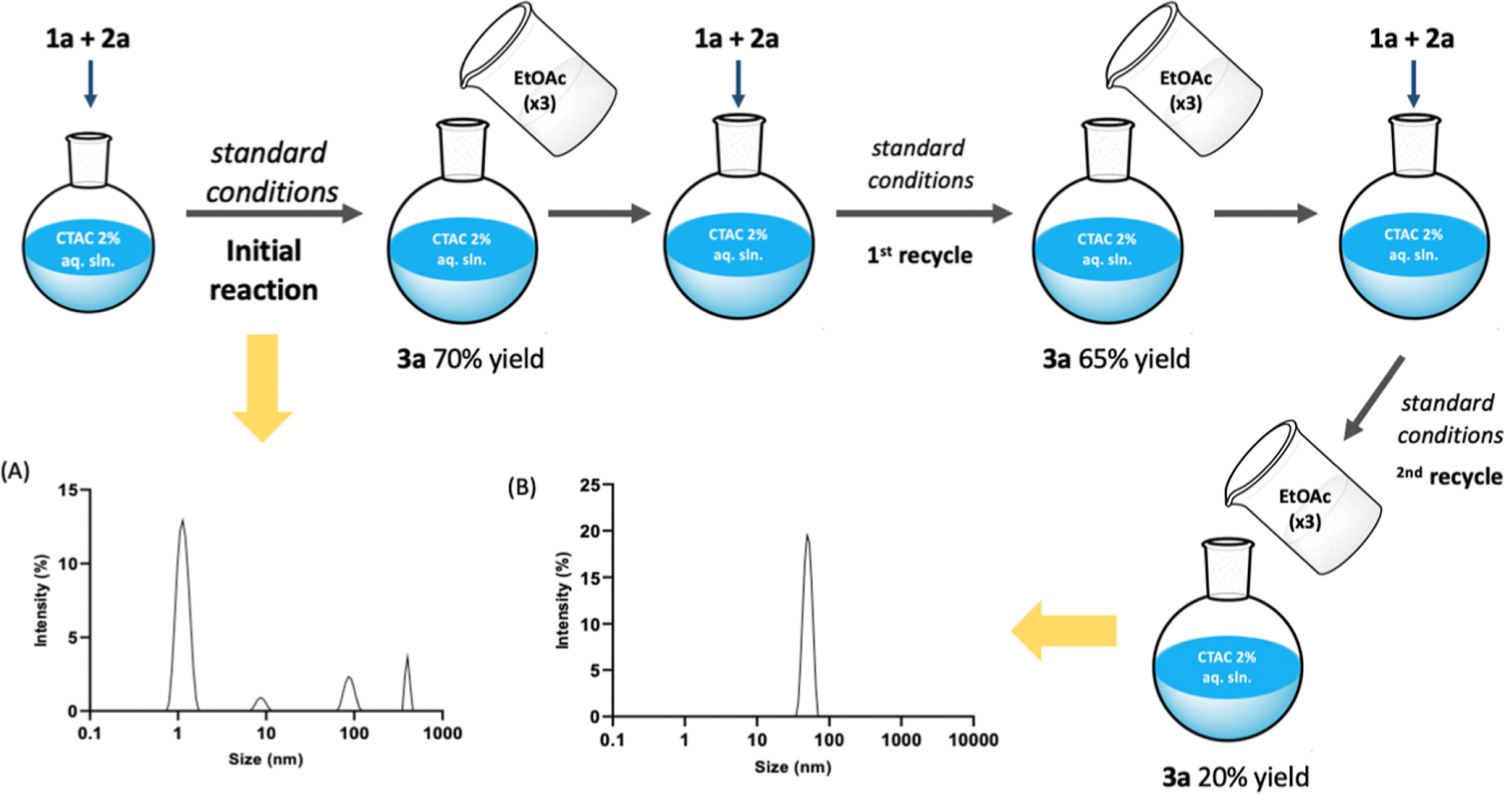
Recycling of the micellar reaction mixture and MADLS analysis of fresh CTAC 2% aq. solution (A) and CTAC 2% aq. solution after two recycles with ethyl acetate (B).

To investigate the root cause of this trend, a dynamic light scattering analysis of the reaction media before (i.e., a CTAC 2% aq. sln.) and after two recycles (3 reaction runs) was performed. The samples were run in multiangle DLS (MADLS) mode to evaluate properly the presence of nonisotropic scatterers (multimodal sample). The size distribution curve of the untreated CTAC 2% aq. solution (panel A, Figure 5) confirmed the presence of micellar aggregates, consistent with a solution above the critical micelle concentration (CMC), i.e., 0.95 mM at 293.15 K.^22^ In the examined medium, the most abundant species at ca. 1.2 nm in CTAC 2% aq solution completely disappeared after two recycles, when only a population at ca. 50 nm was observed (panel B, Figure 5). This trend suggested that the ethyl acetate extraction effectively disrupted or removed a substantial fraction of the micellar structures from the aqueous phase. Ethyl acetate, while only moderately polar, could partition hydrophobic components or interact with the hydrophobic tails of the CTAC micelle, thereby destabilizing the micellar assembly. Overall, the MADLS data clearly demonstrated that ethyl acetate extraction led to a depletion of CTAC micelles in the aqueous phase, thus being one of the main factors accounting for the yield decrease observed over consecutive recycling steps.

## Conclusions

In conclusion, arylazo sulfones have been exploited here as 1,3-dipole acceptors in a [3 + 2] cycloaddition reaction with the carboanions of α-acidic isonitriles such as TosMIC and related analogues. Notably, arylazo sulfones, representing safer and stable analogues of diazonium salts, were confirmed to be valuable reservoirs of multiple reactivities not completely explored so far. The developed reaction conditions rely on the use of a water-based reaction medium, namely a CTAC 2% aq. solution, in accordance with the increasing global need to access sustainable organic synthetic methodologies. In order to further promote the exploitation of micellar catalysis, the reaction medium has been characterized at the molecular level via solution NMR techniques. These studies provided a rational explanation for the experimental data observed and a guideline to the choice of the optimal micellar medium on the basis of the reaction components’ physicochemical nature. The recyclability of the reaction medium posed major challenges, as also highlighted by DLS studies, pointing out the need to invest more time and resources in such a task and accomplish full and real sustainable organic synthesis via micellar catalysis.

## Experimental Section

### General Methods

Commercially available reagents and solvents were used without further purification. Photochemical reactions were carried out using a PhotoRedOx Box (EvoluChem) equipped with 30 W blue LEDs (EvoluChem, model: HCK1012 01-008, wavelength 450 nm, LEDs: CREE XPE). A holder suitable for 4 mL scintillation vials (45 mm × 14.7 mm) was fitted within the box to allow a fixed distance of the samples from the light source. All reactions were routinely checked by thin-layer chromatography (TLC) on 5 × 20 cm plates with a layer thickness of 0.25 mm (silica gel 60 *F*_254_) and monitored by using UV and/or KMnO_4_ as the revelation method. Column chromatography purifications were carried out on silica gel 60 (70–230 mesh ASTM) using the reported eluents. Proton 1H NMR spectra were recorded on a Bruker Avance NEO 400 or Bruker Avance 300 MHz, and proton-decoupled carbon ^13^C­{^1^H} NMR spectra were recorded either at 101 or 75 MHz. Proton-decoupled fluorine ^19^F­{^1^H} NMR spectra were recorded at 376 MHz. Structural assignments were made with additional information from the gCOSY, gHSQC, and gHMBC experiments. Chemical shifts (δ) are reported in parts per million (ppm) relative to the residual solvent as the internal reference. The following abbreviations are used for the multiplicities: s: singlet; d: doublet; t: triplet; q: quadruplet; quint: quintuplet; hept: heptaplet; m: multiplet or overlap of nonequivalent resonances; and br s: broadened singlet. Experiments for structure elucidation were performed in the reported deuterated solvents, at 25 °C, with an RT-DR-BF/1H-5 mm-OZ SmartProbe. Chemical shifts (δ) are reported in parts per million (ppm) relative to the residual solvent peak; coupling constants (*J*) are reported in hertz (Hz). High-resolution mass spectra were determined on an AEI MS-9 using electrospray ionization (ESI) and a time-of-flight (TOF). The spectra were recorded by infusion into the ESI source using MeOH as the solvent. Dynamic light scattering (DLS) analysis was performed on a Malvern Zetasizer Advance Ultra (Malvern Instruments Ltd., UK), equipped with a 633 nm laser and a multiangle detection (13°, 90°, 173°). Measurements were carried out in a ZEN2112 ultralow volume Quartz Glass cuvette. The particle concentration was directly calculated by the software. Measurements were performed in triplicate to ensure reproducibility, and results are reported as the average ± standard deviation.

### General Procedure for the Synthesis of Compounds **3a–3v** (Method A)

To a 4 mL colorless screw-cap glass vial equipped with a magnetic stir bar were added the arylazo sulfone (0.09 mmol, 1 equiv), K_2_CO_3_ (0.18 mmol, 2 equiv), and the isocyanide (0.18 mmol, 2 equiv). These were dissolved in a 2% aqueous micellar solution of CTAC (0.1 M, 0.9 mL). The resulting mixture was stirred for 20 h in a PhotoRedOx Box (EvoluChem) equipped with 30 W blue LEDs (450 nm) at room temperature. The reaction was analyzed by means of TLC, and the crude was diluted with ethyl acetate. The aqueous layer was extracted 3 times with ethyl acetate, and the collected organic phases were dried over Na_2_SO_4_. After evaporation of the solvent, the crude material was purified with the specified mobile phase via silica-gel chromatography.

### General Procedure for the Synthesis of Compounds **3w** and **3x** (Method B)

To a 4 mL colorless screw-cap glass vial equipped with a magnetic stir bar were added the arylazo sulfone (0.09 mmol, 1 equiv), K_2_CO_3_ (0.18 mmol, 2 equiv), and the isocyanide (0.18 mmol, 2 equiv). These were dissolved in a 2% aqueous micellar solution of CTAC (0.1 M, 0.9 mL). The resulting mixture was stirred at room temperature for 20 h. The reaction was analyzed by means of TLC, and the crude was diluted with ethyl acetate. The aqueous layer was extracted 3 times with ethyl acetate, and the collected organic phases were dried over Na_2_SO_4_. After the solvent was evaporated, the crude material was purified with the specified mobile phase via silica-gel chromatography.

### General Procedure for the Preparation of Triazole **3** on the **1** mmol Scale

To a 20 mL colorless screw-cap glass vial equipped with a magnetic stir bar were added the arylazo sulfone (1 mmol, 1 equiv, 214.2 mg), K_2_CO_3_ (2 mmol, 2 equiv, 276.4 mg), and the isocyanide (2 mmol, 2 equiv, 390.5 mg). The compounds were dissolved in a 2% aqueous micellar solution of CTAC (0.1 M, 10 mL), and the resulting mixture was stirred for 20 h in a PhotoRedOx Box (EvoluChem) equipped with 30 W blue LEDs (450 nm) at room temperature. The reaction was analyzed by means of TLC, and the crude was diluted with ethyl acetate. The aqueous layer was extracted with ethyl acetate (3 × 30 mL), and the collected organic phases were dried over Na_2_SO_4_. After evaporation of the solvent, the crude material was purified with the specified mobile phase via silica-gel chromatography (*n*-hexane/ethyl acetate from 70:30 to 50:50). Amount of product **3a** isolated: 224.3 mg, reaction yield: 68%.

### NMR Spectroscopy

NMR samples were prepared by dissolving TosMIC (**2a**) (200 mM), arylazo sulfone **1a** (100 mM), and K_2_CO_3_ (200 mM) in 0.54 mL of either SDS, CTAC, or TPGS 2% (w/v) aqueous micellar solutions and 0.06 mL of D_2_O (final volume = 0.60 mL). All NMR experiments were conducted on a Bruker Avance NEO 600 MHz spectrometer equipped with a z-gradient 5 mm triple-resonance probe. The 1D ^1^H and 2D ^1^H-^1^H NOESY spectra were processed and analyzed by using Bruker TopSpin 4.1.1. Phase-sensitive NOESY spectra were acquired using the States-TPPI method,^23,24^ with 4096 complex points in t_2_ and 256 t_1_ increments, a mixing time of 500 ms, a spectral width of 9615.38 Hz, an acquisition time of 0.213 s, and a relaxation delay (D1) of 4.0 s. The 90° pulse length (P1) was 10.25 μs, and 16 scans were collected per increment. Titration experiments were carried out with arylazo sulfone **1**a by recording 1D ^1^H NMR spectra in SDS, CTAC, and TPGS 2% micellar solutions upon incremental addition of MnCl_2_·4H_2_O at the following concentrations: 0.05, 0.1, 0.15, 0.2, 0.25, 0.3, 0.4, and 0.5 mM. These experiments were designed to probe potential interactions between the azo compound and the micellar environment via paramagnetic relaxation enhancement (PRE). The titration with Mn^2+^ was not performed in the presence of TosMIC **2a** and K_2_CO_3_ due to the precipitation of Mn^2+^ as MnCO_3_ or Mn­(OH)_2_ under basic conditions, which prevents reliable analysis of paramagnetic relaxation effects.

## Supplementary Material



## Data Availability

The data underlying this study are available in the published article and its Supporting Information.

## Notes

Copies of ^1^H-, 2D NOESY, and ^13^C-NMR spectra (PDF)
