# Supplementary material for: Arylazo Sulfones as 1,3-Dipole Acceptors in the (Photo)-Micellar van Leusen Triazole Synthesis
Source: ACS Org Inorg Au. 2025 Sep 15;5(6):539–47. doi: 10.1021/acsorginorgau.5c00080 (PMC12679303; doi:10.1021/acsorginorgau.5c00080)

SUPPORTING INFORMATION

Arylazo Sulfones as 1,3-dipole acceptors in the (Photo)-Micellar van Leusen Triazole Synthesis

Carmine Volpe,[a]‡ Luca Nicchio,[b]‡ Federica Santoro,[a]‡ Teresa Silvestri,[a] Anna Di Porzio,[a] Camilla Russo,[a] Fabiana Quaglia,[a] Antonio Randazzo,[a] Alfonso Carotenuto,[a] Diego Brancaccio,*[a] Stefano Protti,*[b] and Mariateresa Giustiniano*[a]

[a] *Department of Pharmacy, University of Naples Federico II, via D. Montesano 49, 80131, Napoli, Italy*

[b] *PhotoGreen Lab, Department of Chemistry, University of Pavia, viale Taramelli 12, 27100, Pavia, Italy.*

‡ *The authors contributed equally to this work.*

Table of Contents

1. **Synthesis of arylazo sulfones** S3
2. **Synthesis of TosMIC analogues** S4
3. **Characterization data for 3a-3x** S5
4. **Additional experiments** S13
5. **Characterization of reaction medium at molecular level via 1D ^{1}H and 2D ^{1}H-^{1}H NMR solution techniques** S14
6. **References** S21
7. **Copies of ^{1}H and ^{13}C{^{1}H} NMR** S22

1. Synthesis of arylazo sulfones 1a-1n.

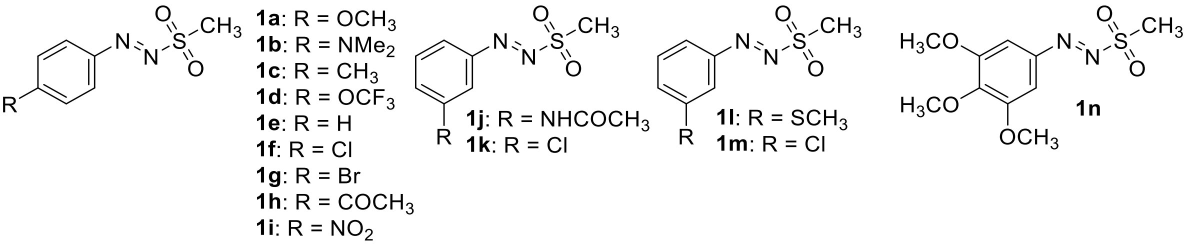

Scheme S1. Arylazo sulfones **1** employed in the present work.

Arylazo sulfones have been prepared according to the experimental procedure reported in literature.[S1] A 100 mL round-bottom flask was charged with the chosen arylamine (10 mmol), EtOH (2 mL) and HBF_4 (48%$_{aq}$, 6 mL). After stirring for 15 minutes, an aqueous solution of $NaNO_2$ (759 mg, 1.1 equiv, dissolved in a minimum amount of deionized H_2O) was added dropwise at 0 °C. The mixture was stirred for an additional 30 min at 0 °C, then 50 mL of diethyl ether were added, and the resulting arenediazonium tetrafluoroborate was filtered and washed with additional diethyl ether. The obtained product was employed in the next step without further purification. In a 250 mL round-bottom flask, the previously prepared arenediazonium tetrafluoroborate (10 mmol) was suspended in DCM (100 mL) and stirred at 0 °C; then the chosen sulfinate (1.1 equiv.) was added to the reaction system in one portion. The suspension was left at room temperature and stirred overnight. The resulting mixture was filtered, and the obtained solution evaporated under vacuo. The crude was revived with cold DCM and the product was precipitated by adding *n*-hexane and then filtered and dried in vacuo. Spectroscopical data of **1a-n** are in accordance with the literature.[S1]

2. Synthesis of TosMIC analogues and characterization data.

TosMIC [(tolylsulfonyl)methyl isocyanide] analogues have been synthesized via a three-step sulfide to isonitrile synthesis according to a previous experimental procedure reported in literature.[S2] Spectroscopical data were in accordance with the literature, except for **2d**, which characterization has been reported below.

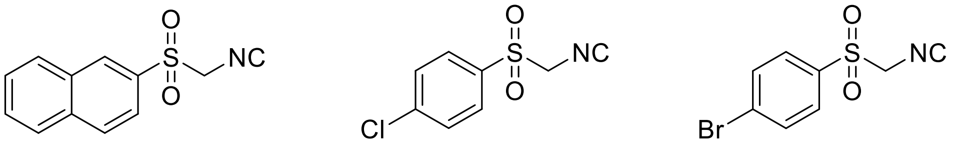

2b 2c 2d

1-bromo-4-((isocyanomethyl)sulfonyl)benzene (2d)

Compound **2d** was prepared via a three-step sulfide to isocyanide synthesis reported in literature from 4-bromobenzenethiol (5.28 mmol, 1 eq.).[S2]

^{1}H NMR (400 MHz, $CDCl_3$) δ 7.83 – 7.79 (m, 2H), 7.76 – 7.73 (m, 2H), 4.53 (s, 2H); $^{13}C\{^{1}H\}$ NMR (101 MHz, $CDCl_3$) δ 166.9, 133.9, 133.2, 131.4, 130.9, 61.0.

HRMS (ESI-TOF) m/z: calcd. for $C_{10}H_{16}{}^{35}BrNO_3S^+$ $[M+CH_3OHH]^+$: 291.9638, found 291.9644.

HRMS (ESI-TOF) m/z: calcd. for $C_{10}H_{16}{}^{35}BrNO_3S^+$ $[M+CH_3OHH]^+$: 293.9618, found 293.9620.

3. **Characterization data for compounds 3a-3x.**

5-tosyl-1-(4-(methoxyphenyl)-1*H*-1,2,4-triazole (3a).

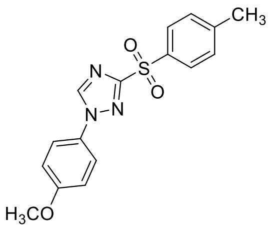

Starting from arylazo sulfone **1a** (19.3 mg, 0.09 mmol), potassium carbonate (24.9 mg, 0.18 mmol), and 1-((isocyanomethyl)sulfonyl)-4-methylbenzene (35.1 mg, 0.18 mmol). Isolated by column chromatography (*n*-hexane/ethyl acetate from 70:30 to 50:50). Yellowish powder (71% yield, 21.0 mg).

(3a). ^{1}H NMR (400 MHz, $CDCl_3$) δ 8.51 (s, 1H), 7.93 (d, *J* = 8.1 Hz, 2H), 7.55 – 7.43 (m, 2H), 7.28 (d, *J* = 8.1 Hz, 2H), 6.93 – 6.84 (m, 2H), 3.74 (s, 3H), 2.34 (s, 3H).

^{13}C{^{1}H} NMR (101 MHz, $CDCl_3$) δ 163.4, 160.2, 145.8, 136.4, 130.0, 129.3, 128.7, 122.3, 114.9, 55.7, 21.7.

HRMS (ESI-TOF) m/z: calcd. for $C_{16}H_{16}N_3O_3S^+$ $[M+H]^+$: 330.0907, found 330.0902.

1-(4-methoxyphenyl)-5-(naphthalen-2-ylsulfonyl)-1*H*-1,2,4-triazole (3b).

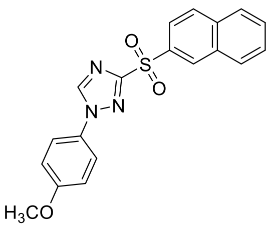

Starting from arylazo sulfone **1a** (19.3 mg, 0.09 mmol), potassium carbonate (24.9 mg, 0.18 mmol), and 2-((isocyanomethyl)sulfonyl)naphthalene (41.6 mg, 0.18 mmol). Isolated by column chromatography (*n*-hexane/ethyl acetate from 90:10 to 70:30). Yellowish powder (75% yield, 24.5 mg).

(3b). ^{1}H NMR (400 MHz, $CDCl_3$) δ 8.77 – 8.74 (m, 1H), 8.46 (s, 1H), 8.11 (dd, *J* = 8.7, 1.9 Hz, 1H), 8.03 – 7.97 (m, 2H), 7.90 (d, *J* = 8.0 Hz, 1H), 7.69 – 7.58 (m, 2H), 7.57 – 7.52 (m, 2H), 7.00 – 6.95 (m, 2H), 3.84 (s, 3H); ^{13}C{^{1}H} NMR (101 MHz, $CDCl_3$) δ 161.7, 160.3, 136.0, 135.6, 132.2, 129.7, 129.6, 129.6, 129.3, 128.0, 127.7, 123.2, 122.5, 114.9, 55.7.

HRMS (ESI-TOF) m/z: calcd. for $C_{19}H_{15}N_3O_3SNa^+$ $[M+Na]^+$: 388.0726, found 388.0723.

1-(4-chlorophenyl)-5-(naphthalen-2-ylsulfonyl)-1*H*-1,2,4-triazole (3c).

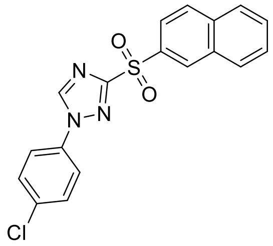

Starting from arylazo sulfone **1f** (19.7 mg, 0.09 mmol), potassium carbonate (24.9 mg, 0.18 mmol), and 2-((isocyanomethyl)sulfonyl)naphthalene (41.6 mg, 0.18 mmol). Isolated by column chromatography (*n*-hexane/ethyl acetate from 95:5 to 70:30). Yellowish powder (63% yield, 20.9 mg).

(3c) ^{1}H NMR (400 MHz, $CDCl_3$) δ 8.76 (d, *J* = 1.9 Hz, 1H), 8.54 (s, 1H), 8.11 (dd, *J* = 8.8, 2.0 Hz, 1H), 8.05 – 7.99 (m, 2H), 7.92 (d, *J* = 8.1 Hz, 1H), 7.71 – 7.61 (m, 4H), 7.52 – 7.46 (m, 2H). ^{13}C{^{1}H} NMR (101 MHz, $CDCl_3$) δ 164.1, 135.7, 135.5, 134.6, 132.2, 130.9, 130.2, 129.7, 128.0, 127.8, 123.2, 121.9.

HRMS (ESI-TOF) m/z: calcd. for $C_{18}H_{13}{}^{17}ClN_3O_2S^+$ $[M+H]^+$: 370.0412, found 370.0407.

HRMS (ESI-TOF) m/z: calcd. for $C_{18}H_{13}{}^{19}ClN_3O_2S^+$ [M+H]$^+$: 372.0383, found 372.0383.

5-((4-chlorophenyl)sulfonyl)-1-(4-methoxyphenyl)-1*H*-1,2,4-triazole (3d).

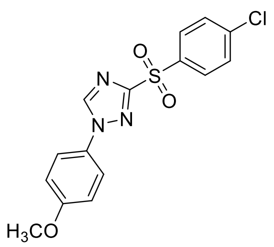

Starting from arylazo sulfone **1a** (19.3 mg, 0.09 mmol), potassium carbonate (24.9 mg, 0.18 mmol), and 1-chloro-4-((isocyanomethyl)sulfonyl)benzene (38.8 mg, 0.18 mmol). Isolated by column chromatography (*n*-hexane/ethyl acetate from 90:10 to 80:20). Yellowish powder (90% yield, 28.2 mg).

(3d). ^{1}H NMR (400 MHz, $CDCl_3$) δ 8.47 (s, 1H), 8.10 (d, *J* = 8.3 Hz, 2H), 7.56 (d, *J* = 6.3 Hz, 2H), 7.54 (d, *J* = 6.1 Hz, 2H), 7.00 (d, *J* = 8.6 Hz, 2H), 3.86 (s, 3H).

$^{13}C\{^1H\}$ NMR (101 MHz, $CDCl_3$) δ 163.1, 160.4, 141.2, 137.6, 130.3, 129.8, 129.3, 122.4, 115.0, 55.7.

HRMS (ESI-TOF) m/z: calcd. for $C_{15}H_{14}{}^{17}ClN_3O_3S^+$ [M+H]$^+$: 350.0361, found 350.0353.

HRMS (ESI-TOF) m/z: calcd. for $C_{15}H_{14}{}^{19}ClN_3O_3S^+$ [M+H]$^+$: 352.0332, found 352.0330.

1-(4-chlorophenyl)-5-((4-chlorophenyl)sulfonyl)-1*H*-1,2,4-triazole (3e).

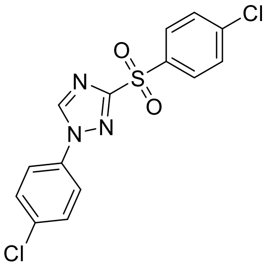

Starting from arylazo sulfone **1f** (19.7 mg, 0.09 mmol), potassium carbonate (24.9 mg, 0.18 mmol), and 1-chloro-4-((isocyanomethyl)sulfonyl)benzene (38.8 mg, 0.18 mmol). Isolated by column chromatography (*n*-hexane/ethyl acetate from 95:5 to 85:15). Yellowish powder (66% yield, 20.9 mg).

(3e). ^{1}H NMR (400 MHz, $CDCl_3$) δ 8.55 (s, 1H), 8.10 (d, *J* = 8.3 Hz, 2H), 7.63 (d, *J* = 8.6 Hz, 2H), 7.56 (d, *J* = 8.6 Hz, 2H), 7.51 (d, *J* = 8.9 Hz, 2H). $^{13}C\{^1H\}$ NMR (101 MHz, $CDCl_3$) δ 141.4, 137.3, 135.6, 134.5, 130.4, 130.2, 129.8, 121.9.

HRMS (ESI-TOF) m/z: calcd. for $C_{14}H_{10}{}^{17}Cl_2N_3O_2S^+$ [M+H]$^+$: 353.9866, found 353.9861.

HRMS (ESI-TOF) m/z: calcd. for $C_{14}H_{10}{}^{19}Cl_2N_3O_2S^+$ [M+H]$^+$: 355.9836, found 355.9834.

5-((4-chlorophenyl)sulfonyl)-1-(*p*-tolyl)-1*H*-1,2,4-triazole (3f).

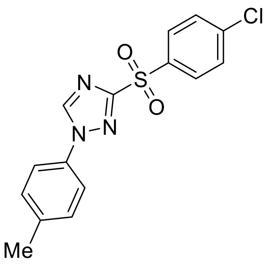

Starting from arylazo sulfone **1c** (17.8 mg, 0.08 mmol), potassium carbonate (24.9 mg, 0.16 mmol), and 1-chloro-4-((isocyanomethyl)sulfonyl)benzene (38.8 mg, 0.16 mmol). Isolated by column chromatography (*n*-hexane/ethyl acetate 90:10). Yellowish powder (62% yield, 18.6 mg).

(3f). ^{1}H NMR (400 MHz, $CDCl_3$) δ 8.52 (s, 1H), 8.13 – 8.07 (m, 2H), 7.57 – 7.50 (m, 4H), 7.31 (d, *J* = 8.2 Hz, 2H), 2.41 (s, 3H). $^{13}C\{^1H\}$ NMR (101 MHz, $CDCl_3$) δ 163.3, 141.2, 139.9, 137.5, 133.7, 130.5, 130.3, 129.7, 120.6, 21.2.

HRMS (ESI-TOF) m/z: calcd. for $C_{15}H_{13}{}^{17}ClN_3O_2S^+$ [M+H]$^+$: 334.0412, found 334.0404.

HRMS (ESI-TOF) m/z: calcd. for $C_{15}H_{13}{}^{19}ClN_3O_2S^+$ [M+H]$^+$: 336.0383, found 336.0380.

5-((4-bromophenyl)sulfonyl)-1-(4-methoxyphenyl)-1*H*-1,2,4-triazole (3g).

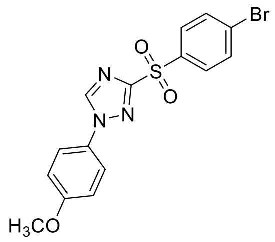

Starting from arylazo sulfone **1a** (19.3 mg, 0.09 mmol), potassium carbonate (24.9 mg, 0.18 mmol), and 1-bromo-4-((isocyanomethyl)sulfonyl)benzene (46.8 mg, 0.18 mmol). Isolated by column chromatography (*n*-hexane/ethyl acetate 9:1). Yellowish powder (87% yield, 31 mg).

(3g). ^{1}H NMR (400 MHz, $CDCl_3$) δ 8.46 (s, 1H), 8.04 – 8.00 (m, 2H), 7.74 – 7.69 (m, 2H), 7.58 – 7.53 (m, 2H), 7.03 – 6.98 (m, 2H), 3.86 (s, 3H); ^{13}C{^{1}H} NMR (101 MHz, $CDCl_3$) δ 163.1, 160.4, 138.1, 132.7, 130.3, 129.9, 129.3, 122.5, 115.0, 55.7.

HRMS (ESI-TOF) m/z: calcd. for $C_{15}H_{13}{}^{35}BrN_3O_3S^+$ [M+H]$^+$: 393.9856, found 393.9857.

HRMS (ESI-TOF) m/z: calcd. for $C_{15}H_{13}{}^{37}BrN_3O_3S^+$ [M+H]$^+$: 395.9836, found 395.9837.

5-((4-bromophenyl)sulfonyl)-1-(*p*-tolyl)-1*H*-1,2,4-triazole (3h).

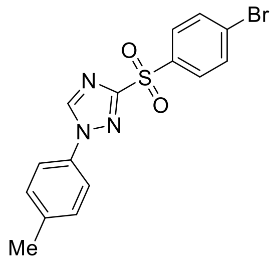

Starting from arylazo sulfone **1c** (17.8 mg, 0.09 mmol), potassium carbonate (24.9 mg, 0.18 mmol), and 1-bromo-4-((isocyanomethyl)sulfonyl)benzene (46.8 mg, 0.18 mmol). Isolated by column chromatography (*n*-hexane/ethyl acetate from 95:5 to 90:10). Yellowish powder (87% yield, 29.6 mg).

(3h). ^{1}H NMR (400 MHz, $CDCl_3$) δ 8.52 (s, 1H), 8.02 (d, *J* = 8.2 Hz, 2H), 7.71 (d, *J* = 8.2 Hz, 2H), 7.53 (d, *J* = 8.0 Hz, 2H), 7.31 (d, *J* = 8.0 Hz, 2H), 2.42 (s, 3H). ^{13}C{^{1}H} (101 MHz, $CDCl_3$) δ 163.2, 139.9, 138.1, 133.7, 132.7, 130.5, 130.3, 129.9, 120.6, 21.2.

HRMS (ESI-TOF) m/z: calcd. for $C_{15}H_{13}{}^{35}BrN_3O_2S^+$ [M+H]$^+$: 377.9907, found 377.9902.

HRMS (ESI-TOF) m/z: calcd. for $C_{15}H_{13}{}^{37}BrN_3O_2S^+$ [M+H]$^+$: 379.9886, found 379.9881.

5-((4-bromophenyl)sulfonyl)-1-(4-chlorophenyl)-1*H*-1,2,4-triazole (3i).

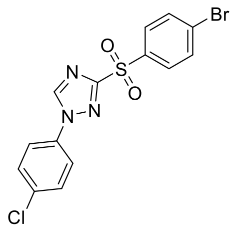

Starting from arylazo sulfone **1f** (19.7 mg, 0.09 mmol), potassium carbonate (24.9 mg, 0.18 mmol), and 1-bromo-4-((isocyanomethyl)sulfonyl)benzene (46.8 mg, 0.18 mmol). Isolated by column chromatography (*n*-hexane/ethyl acetate from 95:5 to 90:10). Yellowish powder (72% yield, 25.8 mg).

(3i). ^{1}H NMR (400 MHz, $CDCl_3$) δ 8.55 (s, 1H), 8.06 – 7.99 (m, 2H), 7.76 – 7.69 (m, 2H), 7.66 – 7.60 (m, 2H), 7.54 – 7.49 (m, 2H). ^{13}C{^{1}H} (101 MHz, $CDCl_3$) δ 137.8, 135.6, 134.5, 132.8, 130.4, 130.2, 130.1, 121.9.

HRMS (ESI-TOF) m/z: calcd. for $C_{14}H_{10}{}^{35}Br^{17}ClN_3O_2S^+$ [M+H]$^+$: 397.9361, found 397.9362.

HRMS (ESI-TOF) m/z: calcd. for $C_{14}H_{10}{}^{37}Br^{19}ClN_3O_2S^+$ [M+H]$^+$: 399.9340, found 399.9339.

Methyl 1-(4-methoxyphenyl)-1*H*-1,2,4-triazole-5-carboxylate (3j).

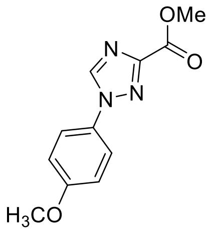

Starting from arylazo sulfone **1a** (17.8 mg, 0.09 mmol), potassium carbonate (24.9 mg, 0.18 mmol), and methylisocyanoacetate (16 μl, 0.18 mmol). Isolated by column chromatography (*n*-hexane/ethyl acetate from 95:5 to 90:10). Yellowish powder (64% yield, 13.5 mg).

(3j). ^{1}H NMR (400 MHz, $CDCl_3$) δ 8.57 (s, 1H), 7.63 – 7.58 (m, 2H), 7.32 (d, *J* = 8.1 Hz, 2H), 4.04 (s, 3H), 2.43 (s, 3H). $^{13}C\{^{1}H\}$ (101 MHz, $CDCl_3$) δ 160.2, 139.4, 134.1, 130.4, 120.5, 52.9, 21.1.

Compound unstable upon HRMS analysis.

***N*,*N*-dimethyl-4-(3-tosyl-1*H*-1,2,4-triazol-1-yl)aniline (3k).**

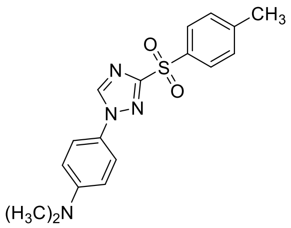

Starting from arylazo sulfone **1b** (20.5 mg, 0.09 mmol), potassium carbonate (24.9 mg, 0.18 mmol), and 1-((isocyanomethyl)sulfonyl)-4-methylbenzene (35.1 mg, 0.18 mmol). Isolated by column chromatography (*n*-hexane/ethyl acetate from 90:10 to 70:30). Yellowish powder (79% yield, 24.3 mg).

(3k). ^{1}H NMR (300 MHz, $CDCl_3$) δ 8.38 (s, 1H), 8.04 (d, *J* = 8.2 Hz, 2H), 7.45 (d, *J* = 9.2 Hz, 2H), 7.35 (d, *J* = 8.2 Hz, 2H), 6.71 (d, *J* = 9.2 Hz, 2H), 3.01 (s, 6H), 2.42 (s, 3H). $^{13}C\{^{1}H\}$ NMR (75 MHz, $CDCl_3$) δ 163.3, 151.0, 145.4, 142.4, 136.5, 130.0, 128.9, 125.5, 122.3, 112.3, 40.5, 21.8.

HRMS (ESI-TOF) m/z: calcd. for $C_{17}H_{19}N_4O_2S^+$ $[M+H]^+$: 343.1223, found 343.1223.

***N*-(3-(5-tosyl-1*H*-1,2,4-triazol-1-yl)phenyl)acetamide (3l).**

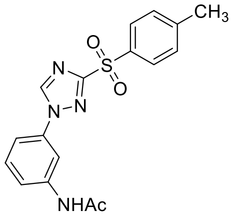

Starting from arylazo sulfone **1j** (21.7 mg, 0.09 mmol), potassium carbonate (24.9 mg, 0.18 mmol), and 1-((isocyanomethyl)sulfonyl)-4-methylbenzene (35.1 mg, 0.18 mmol). Isolated by column chromatography (*n*-hexane/ethyl acetate from 70:30 to 50:50). Yellowish powder (41% yield, 13.2 mg).

(3l). ^{1}H NMR (300 MHz, $CDCl_3$) δ 8.58 (s, 1H), 8.06 – 7.95 (m, 3H), 7.71 (s, 1H), 7.59 – 7.53 (m, 1H), 7.43 – 7.34 (m, 4H), 2.43 (s, 3H), 2.22 (s, 3H). $^{13}C\{^{1}H\}$ NMR (75 MHz, $CDCl_3$) δ 169.0, 164.0, 145.8, 143.0, 139.7, 136.6, 136.0, 130.6, 130.2, 129.0, 120.2, 116.0, 111.7, 24.8, 21.9.

HRMS (ESI-TOF) m/z: calcd. for $C_{17}H_{17}N_4O_3S^+$ $[M+H]^+$: 357.1016, found 357.1012.

1-(2-(methylthio)phenyl)-5-tosyl-1*H*-1,2,4-triazole (3m).

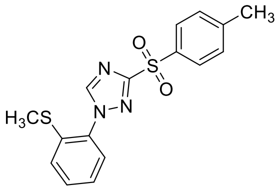

Starting from arylazo sulfone **1l** (20.7 mg, 0.09 mmol), potassium carbonate (24.9 mg, 0.18 mmol), and 1-((isocyanomethyl)sulfonyl)-4-methylbenzene (35.1 mg, 0.18 mmol). Isolated by column chromatography (*n*-hexane/ethyl acetate from 90:10 to 60:40). Yellowish powder (62% yield, 19.3 mg).

(3m). ^{1}H NMR (300 MHz, $CDCl_3$) (300 MHz, $CDCl_3$) δ 8.45 (s, 1H), 8.07 – 8.03 (m, 2H), 7.50 – 7.45 (m, 1H), 7.42 – 7.35 (m, 5H), 7.32 – 7.27 (m, 1H), 2.44 (s, 3H), 2.37 (s, 3H). $^{13}C\{^1H\}$ NMR (75 MHz, $CDCl_3$) δ 163.7, 146.5, 145.6, 136.2, 135.4, 134.6, 130.9, 130.1, 129.0, 128.1, 127.0, 126.3, 21.9, 16.3.

HRMS (ESI-TOF) m/z: calcd. for $C_{16}H_{16}N_3O_2S_2^+$ $[M+H]^+$: 346.0678, found 346.0676.

1-(2-chlorophenyl)-3-tosyl-1*H*-1,2,4-triazole (3n).

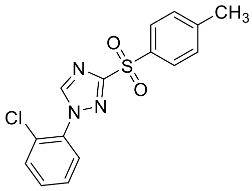

Starting from arylazo sulfone **1m** (19.7 mg, 0.09 mmol), potassium carbonate (24.9 mg, 0.18 mmol), and 1-((isocyanomethyl)sulfonyl)-4-methylbenzene (35.1 mg, 0.18 mmol). Isolated by column chromatography (*n*-hexane/ethyl acetate from 90:10 to 60:40). Yellowish powder (44% yield, 13.2 mg).

(3n). ^{1}H NMR (300 MHz, $CDCl_3$) δ 8.55 (s, 1H), 8.05 (d, J = 8.4 Hz, 2H), 7.62 – 7.53 (m, 2H), 7.48 – 7.42 (m, 2H), 7.41 – 7.35 (m, 2H), 2.44 (s, 3H). $^{13}C\{^1H\}$ NMR (75 MHz, $CDCl_3$) δ 164.0, 146.4, 145.8, 136.0, 134.0, 131.4, 131.0, 130.2, 129.0, 128.6, 128.3, 128.0, 21.9.

HRMS (ESI-TOF) m/z: calcd. for $C_{15}H_{13}{}^{17}ClN_3O_2S^+$ $[M+H]^+$: 334.0412, found 334.0412.

HRMS (ESI-TOF) m/z: calcd. for $C_{15}H_{13}{}^{19}ClN_3O_2S^+$ $[M+H]^+$: 336.0383, found 336.0386.

1-(3-chlorophenyl)-5-tosyl-1*H*-1,2,4-triazole (3o).

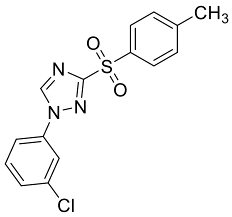

Starting from arylazo sulfone **1k** (19.7 mg, 0.09 mmol), potassium carbonate (24.9 mg, 0.18 mmol), and 1-((isocyanomethyl)sulfonyl)-4-methylbenzene (35.1 mg, 0.18 mmol). Isolated by column chromatography (*n*-hexane/ethyl acetate from 90:10 to 60:40). Yellowish powder (24% yield, 7.2 mg).

(3o). ^{1}H NMR (300 MHz, $CDCl_3$) δ 8.57 (s, 1H), 8.15 – 7.95 (m, 2H), 7.79 – 7.70 (m, 1H), 7.60 – 7.55 (m, 1H), 7.49 – 7.43 (m, 2H), 7.40 – 7.35 (m, 2H), 2.44 (s, 3H). $^{13}C\{^1H\}$ NMR (75 MHz, $CDCl_3$) δ 164.5, 145.9, 142.8, 137.1, 136.1, 1356.0, 131.1, 130.2, 129.7, 129.0, 121.1, 118.6, 21.9.

HRMS (ESI-TOF) m/z: calcd. for $C_{15}H_{13}{}^{17}ClN_3O_2S^+$ $[M+H]^+$: 334.0412, found 334.0411.

HRMS (ESI-TOF) m/z: calcd. for $C_{15}H_{13}{}^{19}ClN_3O_2S^+$ $[M+H]^+$: 336.0383, found 336.0385.

1-(4-chlorophenyl)-5-tosyl-1*H*-1,2,4-triazole (3p).

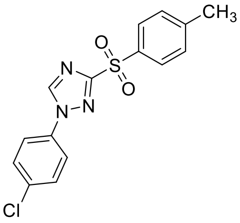

Starting from arylazo sulfone **1f** (19.7 mg, 0.09 mmol), potassium carbonate (24.9 mg, 0.18 mmol), and 1-((isocyanomethyl)sulfonyl)-4-methylbenzene (35.1 mg, 0.18 mmol). Isolated by column chromatography (*n*-hexane/ethyl acetate from 90:10 to 60:40). Yellowish powder (41% yield, 12.3 mg).

(3p). ^{1}H NMR (300 MHz, $CDCl_3$) δ 8.54 (s, 1H), 8.04 (d, *J* = 8.4 Hz, 2H), 7.63 (d, *J* = 9.0 Hz, 2H), 7.49 (d, *J* = 9.0 Hz, 2H), 7.37 (d, *J* = 8.4 Hz, 2H), 2.44 (s, 3H). $^{13}C\{^{1}H\}$ NMR (75 MHz, $CDCl_3$) δ 164.4, 145.8, 142.7, 136.0, 135.5, 134.7, 130.3, 130.2, 129.0, 122.0, 21.9.

HRMS (ESI-TOF) m/z: calcd. for $C_{15}H_{13}{}^{17}ClN_3O_2S^+$ $[M+H]^+$: 334.0412, found 334.0410.

HRMS (ESI-TOF) m/z: calcd. for $C_{15}H_{13}{}^{19}ClN_3O_2S^+$ $[M+H]^+$: 336.0383, found 336.0383.

1-(4-bromophenyl)-5-tosyl-1*H*-1,2,4-triazole (3q).

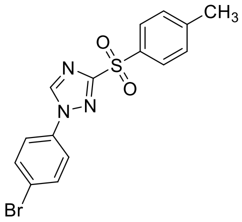

Starting from arylazo sulfone **1g** (23.7 mg, 0.09 mmol), potassium carbonate (24.9 mg, 0.18 mmol), and 1-((isocyanomethyl)sulfonyl)-4-methylbenzene (35.1 mg, 0.18 mmol). Isolated by column chromatography (*n*-hexane/ethyl acetate from 90:10 to 60:40). Yellowish powder (80% yield, 27.2 mg).

(3q). ^{1}H NMR (300 MHz, $CDCl_3$) δ 8.55 (s, 1H), 8.10 – 7.98 (m, 2H), 7.66 – 7.55 (m, 4H), 7.40 – 7.34 (m, 2H), 2.44 (s, 3H). $^{13}C\{^{1}H\}$ NMR (75 MHz, $CDCl_3$) δ 164.4, 145.8, 142.7, 136.0, 135.2, 133.3, 133.1, 130.2, 129.0, 19.8, 122.2, 21.9.

HRMS (ESI-TOF) m/z: calcd. for $C_{15}H_{13}{}^{35}BrN_3O_2S^+$ $[M+H]^+$: 377.9907, found 377.9908.

HRMS (ESI-TOF) m/z: calcd. for $C_{15}H_{13}{}^{37}BrN_3O_2S^+$ $[M+H]^+$: 379.9886, found 379.9883.

5-tosyl-1-(4-(trifluoromethoxy)phenyl)-1*H*-1,2,4-triazole (3r).

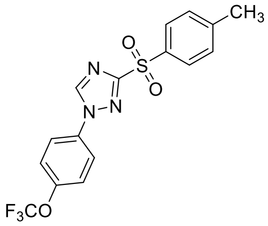

Starting from arylazo sulfone **1d** (24.1 mg, 0.09 mmol), potassium carbonate (24.9 mg, 0.18 mmol), and 1-((isocyanomethyl)sulfonyl)-4-methylbenzene (35.1 mg, 0.18 mmol). Isolated by column chromatography (*n*-hexane/ethyl acetate from 70:30 to 50:50). Yellowish powder (34% yield, 11.7 mg).

(3r). ^{1}H NMR (300 MHz, $CDCl_3$) δ 8.54 (s, 1H), 8.09 – 8.01 (m, 2H), 7.76-7.70 (m, 2H), 7.41 – 7.34 (m, 4H), 2.44 (s, 3H). $^{13}C\{^{1}H\}$ NMR (75 MHz, $CDCl_3$) δ 164.6, 149.7, 145.9, 142.8, 136.0, 134.6, 130.2, 129.1, 122.6 (q, *J* = 1.0 Hz), 122.4, 120.4 (q, *J* = 258.9 Hz), 21.9. ^{19}F NMR (376 MHz, $CDCl_3$) δ -58.01.

HRMS (ESI-TOF) m/z: calcd. for $C_{16}H_{13}F_3N_3O_3S^+$ $[M+H]^+$: 384.0624, found 384.0620.

1-phenyl-5-tosyl-1*H*-1,2,4-triazole (3s).

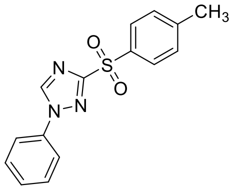

Starting from arylazo sulfone **1e** (16.6 mg, 0.09 mmol), potassium carbonate (24.9 mg, 0.18 mmol), and 1-((isocyanomethyl)sulfonyl)-4-methylbenzene (35.1 mg, 0.18 mmol). Isolated by column chromatography (*n*-hexane/ethyl acetate from 90:10 to 60:40). Yellowish powder (40% yield, 10.8 mg).

(3s). ^{1}H NMR (300 MHz, $CDCl_3$) δ 8.56 (s, 1H), 8.11 – 7.95 (m, 2H), 7.71 – 7.64 (m, 2H), 7.56 – 7.42 (m, 3H), 7.39 – 7.32 (m, 2H), 2.43 (s, 3H). ^{13}C{^{1}H} NMR (75 MHz, $CDCl_3$) δ 164.1, 145.7, 142.8, 136.2, 136.1, 130.1, 130.0, 129.5, 129.0, 120.8, 21.8.

HRMS (ESI-TOF) m/z: calcd. for $C_{15}H_{14}N_3O_2S^+$ [M+H]$^+$: 300.0801, found 300.0879.

1-(p-tolyl)-3-tosyl-1H-1,2,4-triazole (3t).

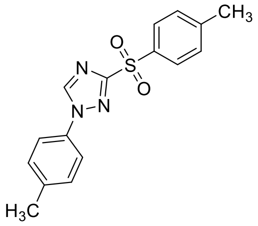

Starting from arylazo sulfone **1c** (17.8 mg, 0.09 mmol), potassium carbonate (24.9 mg, 0.18 mmol), and 1-((isocyanomethyl)sulfonyl)-4-methylbenzene (35.1 mg, 0.18 mmol). Isolated by column chromatography (*n*-hexane/ethyl acetate from 90:10 to 60:40). Yellowish powder (53% yield, 14.9 mg).

(3t). ^{1}H NMR (300 MHz, $CDCl_3$) δ 8.50 (s, 1H), 8.13 – 7.97 (m, 2H), 7.58 – 7.48 (m, 2H), 7.39 – 7.27 (m, 4H), 2.43 (s, 3H), 2.41 (s, 3H).^{13}C{^{1}H} NMR (75 MHz, $CDCl_3$) δ 164.0, 145.6, 142.7, 139.7, 136.3, 134.0, 130.6, 130.1, 129.0, 120.7, 21.9, 21.3.

HRMS (ESI-TOF) m/z: calcd. for $C_{16}H_{16}N_3O_2S^+$ [M+H]$^+$: 314.0958, found 314.0950.

5-tosyl-1-(3,4,5-trimethoxyphenyl)-1*H*-1,2,4-triazole (3u).

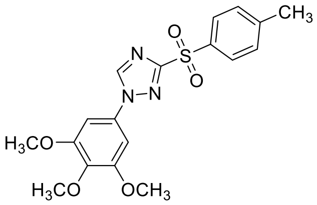

Starting from arylazo sulfone **1m** (29.8 mg, 0.09 mmol), potassium carbonate (24.9 mg, 0.18 mmol), and 1-((isocyanomethyl)sulfonyl)-4-methylbenzene (35.1 mg, 0.18 mmol). Isolated by column chromatography (*n*-hexane/ethyl acetate from 70:30 to 50:50). Yellowish powder (53% yield, 18.6 mg).

(3u). ^{1}H NMR (300 MHz, $CDCl_3$) δ 8.49 (s, 1H), 8.04 (d, *J* = 8.3 Hz, 2H), 7.37 (d, *J* = 8.3 Hz, 2H), 6.84 (s, 2H), 3.91 (s, 6H), 3.87 (s, 3H), 2.43 (s, 3H). ^{13}C{^{1}H} NMR (75 MHz, $CDCl_3$) δ 163.9, 154.2, 145.7, 143.0, 139.1, 136.1, 132.0, 130.1, 129.0, 98.9, 61.2, 56.7, 21.9.

HRMS (ESI-TOF) m/z: calcd. for $C_{18}H_{20}N_3O_5S^+$ [M+H]$^+$: 390.1118, found 390.1112.

1-(4-(1*H*-1,2,4-triazol-1-yl)phenyl)ethan-1-one (3v).

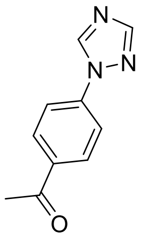

Starting from arylazo sulfone **1h** (17.8 mg, 0.09 mmol), potassium carbonate (24.9 mg, 0.18 mmol), and TosMIC **1a** (35.1 mg, 0.18 mmol). Isolated by column chromatography (*n*-hexane/ethyl acetate from 90:10 to 60:40). Yellowish powder (83% yield, 14 mg) **(3v).** ^{1}H NMR (400 MHz, $CDCl_3$) δ 8.66 (s, 1H), 8.15 – 8.10 (m, 3H), 7.85 – 7.80 (m, 2H), 2.65 (s, 3H). ^{13}C{^{1}H} NMR (101 MHz, $CDCl_3$) δ 196.6, 153.1, 141.1, 140.1, 136.4, 130.2, 119.8, 26.7.

HRMS (ESI-TOF) m/z: calcd. for $C_{10}H_{10}N_3O^+$ [M+H]$^+$: 188.0818, found 188.0814.

1-(4-nitrophenyl)-1*H*-1,2,4-triazole (3w) and **1-(4-nitrophenyl)-3-tosyl-1*H*-1,2,4-triazole (3x).**

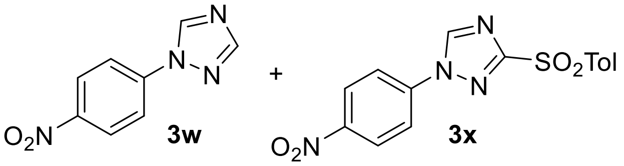

Starting from arylazo sulfone **1i** (17.8 mg, 0.09 mmol), potassium carbonate (24.9 mg, 0.18 mmol), and TosMIC (35.1 mg, 0.18 mmol). purification via preparative TLC (*n*-hexane/ethyl acetate 40:60) afforded **3w** (Yellowish powder (30% isolated yield, 5.1 mg) and **3x** (yellowish powder, 10% isolated yield, 3.1 mg). NMR analyses of the crude point out a 50% yield for **3w** and 50% yield for **3x**.

(3w). ^{1}H NMR (400 MHz, $CDCl_3$) δ 8.70 (s, 1H), 8.43 – 8.38 (m, 2H), 8.17 (s, 1H), 7.95 – 7.90 (m, 2H). ^{13}C{^{1}H} NMR (101 MHz, $CDCl_3$) δ 153.5, 146.8, 129.8, 125.7, 119.9.

HRMS (ESI-TOF) m/z: calcd. for $C_8H_7N_4O_2^+$ [M+H]$^+$: 191.0564, found 191.0562.**(3x).** ^{1}H NMR ^{1}H NMR (400 MHz, $CDCl_3$) δ 8.70 (s, 1H), 8.45 – 8.37 (m, 2H), 8.05 (d, *J* = 8.3 Hz, 2H), 7.95 – 7.91 (m, 2H), 7.39 (d, *J* = 8.3 Hz, 2H), 2.45 (s, 3H).

^{13}C{^{1}H} NMR (101 MHz, $CDCl_3$) δ 147.7, 146.0, 140.3, 135.5, 130.1, 129.0, 125.7, 120.8, 21.8.

HRMS (ESI-TOF) m/z: calcd. for $C_{15}H_{13}N_4O_4^+$ [M+H]$^+$: 345.0652, found 345.0654.

4. Additional experiments.

Experiments in the presence of indole.

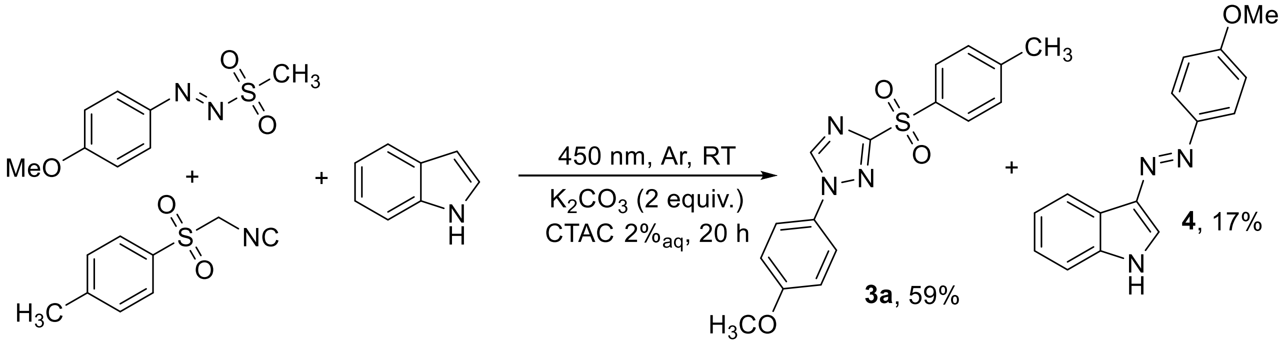

To a 4 mL colorless screw-cap glass vial equipped with a magnetic stir bar were added the arylazo sulfone (0.09 mmol, 1 equiv.), K_2CO_3 (0.18 mol, 2 equiv.), isocyanide (0.18 mmol, 2 equiv.) and indole (0.18 mmol, 2 equiv.). These were dissolved in a 2% aqueous micellar solution of CTAC (0.1 M, 0.9 mL). The resulting mixture was degassed with Argon for 15 min. The Argon purged mixture was stirred for 20 h in a PhotoRedOx Box (EvoluChem™) equipped with 30 W blue LEDs (450 nm), at room temperature. The reaction was analyzed by means of TLC and the crude was extracted 3 times with ethyl acetate, and the collected organic phases were dried over Na_2SO_4. After evaporation of the solvent, the crude material was purified via silica-gel chromatography (Eluent *n*-hexane/ethyl acetate from 90:10 to 50:50) affording 59% of product **3a** along with 17% of 3-((4-methoxyphenyl)diazenyl)-1*H*-indole (**4**). Spectroscopic data of compound **4** are in accordance with the literature.[S3]

Radical trapping experiment

To a 4 mL colorless screw-cap glass vial equipped with a magnetic stir bar were added *p*-methoxyphenylazosulfone (0.09 mmol, 1 equiv.), K_2CO_3 (0.18 mol, 2 equiv.), TosMIC (0.18 mmol, 2 equiv.) and TEMPO (0.09 mmol, 1 equiv.). These were dissolved in a 2% aqueous micellar solution of CTAC (0.1 M, 0.9 mL). The resulting mixture was stirred for 20 h in a PhotoRedOx Box (EvoluChem™) equipped with 30 W blue LEDs (450 nm), at room temperature. The reaction was analyzed by means of TLC and the crude was diluted with ethyl acetate. The aqueous layer was extracted 3 times with ethyl acetate, and the collected organic phases were dried over Na_2SO_4. After evaporation of the solvent, the crude material was dissolved in $CDCl_3$ and the NMR yield was determined using TMB (1,2,3-trimethoxybenzene) as an internal standard.

Aqueous Surfactant Recycling

Reaction was carried out according to General procedure A with *p*-methoxyphenylazosulfone (0.09 mmol, 1 equiv.), K_2CO_3 (0.18 mmol, 2 equiv.), TosMIC (0.18 mmol, 2 equiv.) as starting materials. After the reaction was completed, the crude was diluted with ethyl acetate. The aqueous layer was extracted 3 times with ethyl acetate. To the surfactant solution remaining in the reaction flask appropriate amounts of each reagent were then added: 4-methoxyphenylazosulfone (0.09 mmol, 1 equiv.), K_2CO_3 (0.18 mmol, 2 equiv.), TosMIC (0.18 mmol, 2 equiv.) and the resulting mixture was stirred for 20 h in a PhotoRedOx Box (EvoluChem^{TM}) equipped with 30 W blue LEDs (450 nm), at room temperature, according to the same procedure. This procedure was continued until the surfactant solution had been recycled two times (third reaction run).

5. Characterization of reaction medium at molecular level via NMR solution techniques

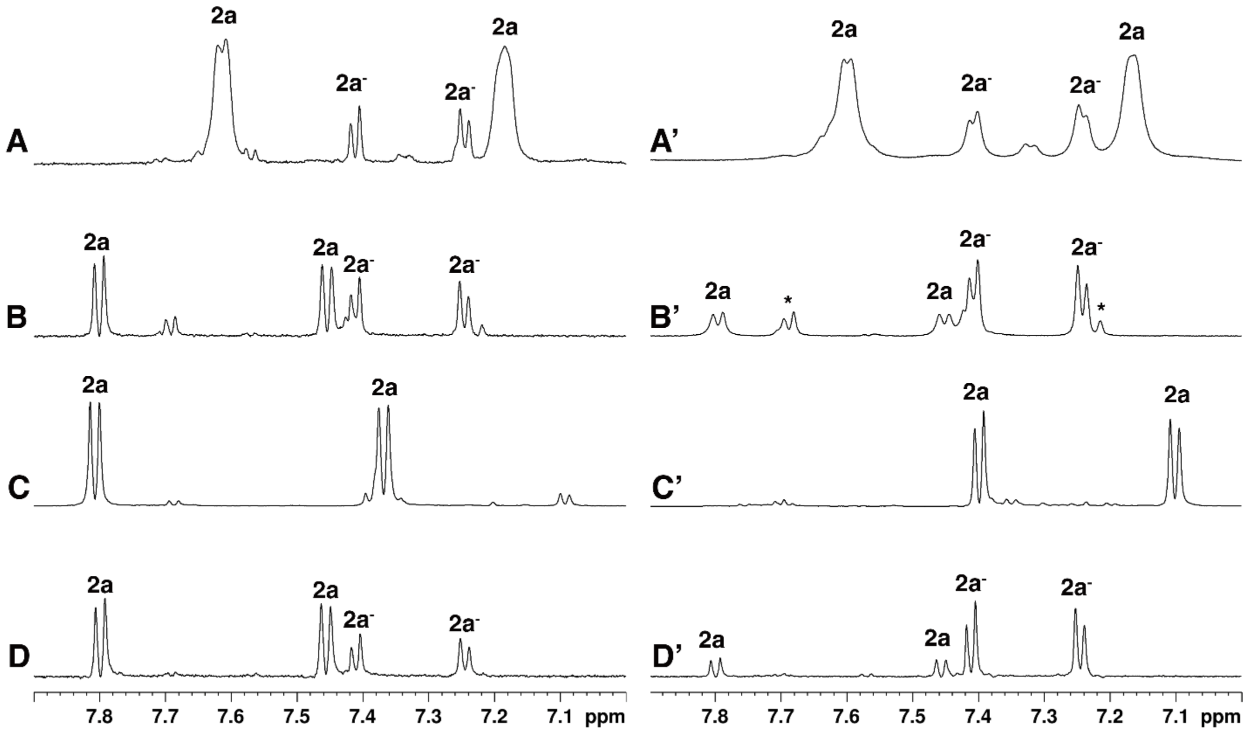

Figure S1. 1D ^{1}H NMR spectra of TosMIC in different aqueous media in the presence of 1 equivalent of K_2CO_3: (A) 2% *w/v* TPGS-750-M; (B) 2% *w/v* SDS; (C) 2% *w/v* CTAC; and (D) pure water. Panels (A′–D′) show spectra of the same samples re-acquired after approximately 24 hours. Signals are assigned to the neutral, undissociated form of TosMIC (**2a**), its dissociated carbanion form (**2a**$^-$), and, in SDS micelles, additional signals (*) corresponding to a hydrated form (formamide derivative) of TosMIC.

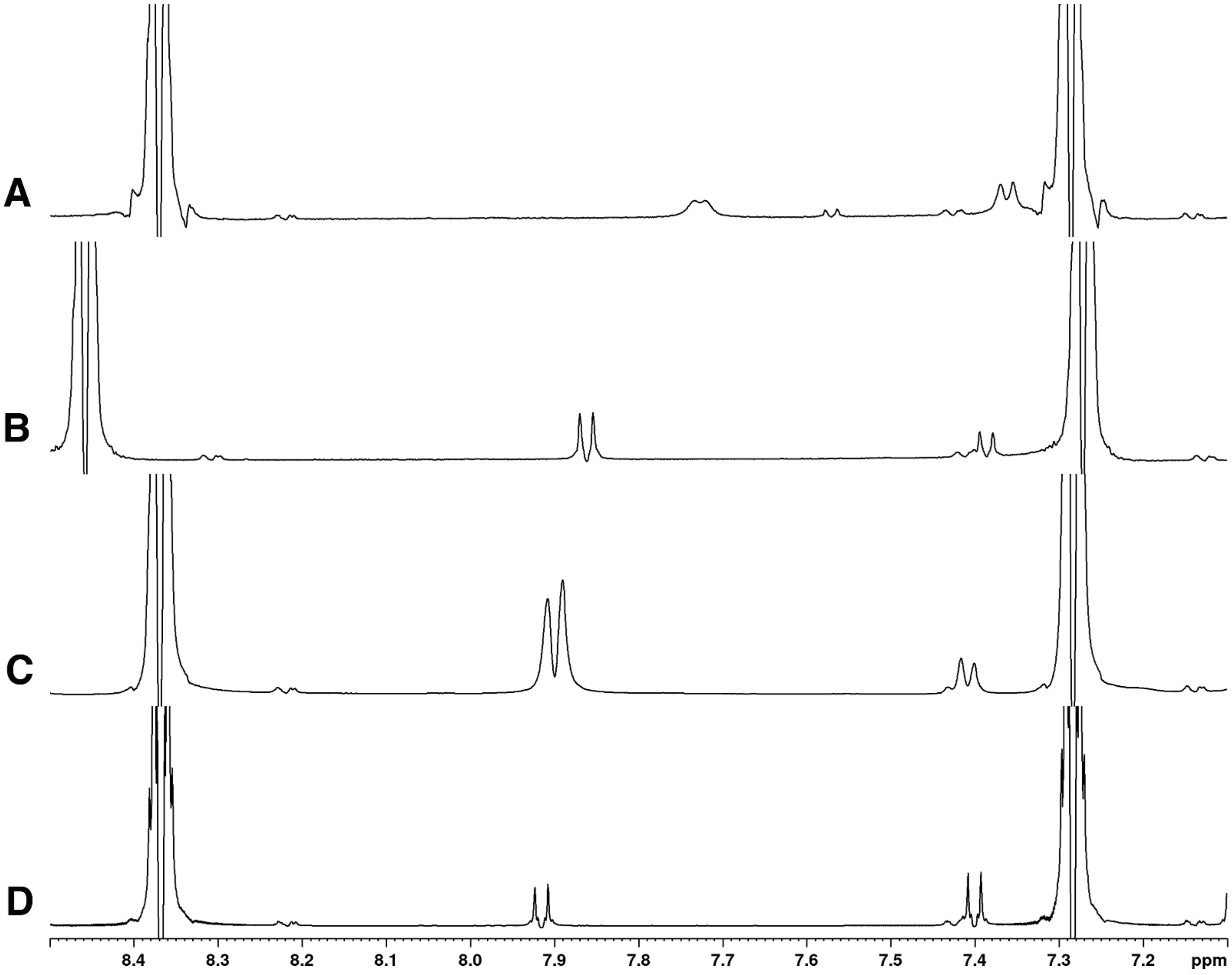

Figure S2. 1D ^{1}H NMR spectra of arylazo sulfone **1a** in: (A) 2% *w/v* TPGS-750-M; (B) 2% *w/v* SDS; (C) 2% *w/v* CTAC; and (D) pure water.

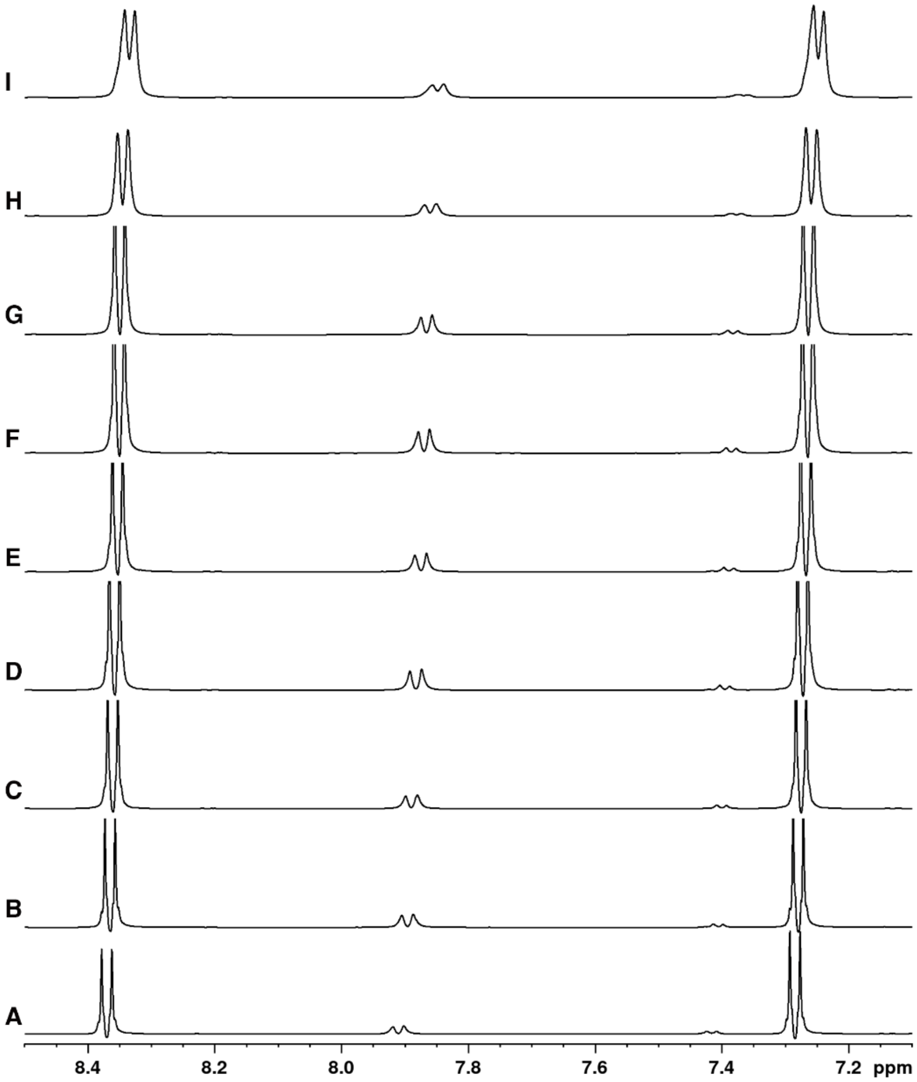

Figure S3. ^{1}H NMR titration of arylazo sulfone **1a** in 2% *w/v* **CTAC** micellar solution with increasing concentrations of $MnCl_2 \cdot 4H_2O$. Spectra A–I correspond to Mn^{2+} concentrations of 0, 0.05, 0.1, 0.15, 0.2, 0.25, 0.3, 0.4, and 0.5 mM, respectively.

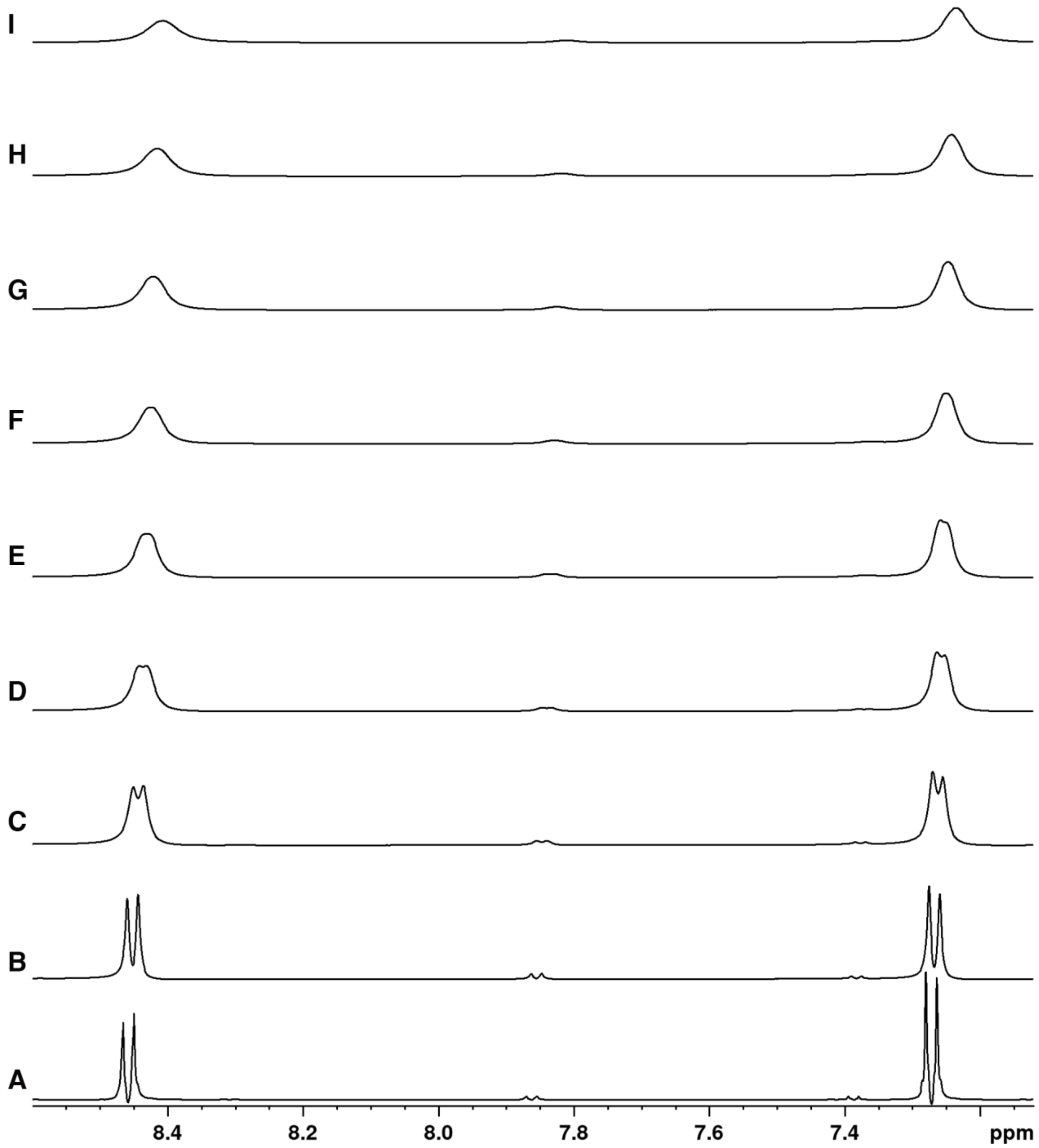

Figure S4. ^{1}H NMR titration of arylazo sulfone **1a** in 2% *w/v* **SDS** micellar solution with increasing concentrations of $MnCl_2{\cdot}4H_2O$. Spectra A–I correspond to Mn^{2+} concentrations of 0, 0.05, 0.1, 0.15, 0.2, 0.25, 0.3, 0.4, and 0.5 mM, respectively.

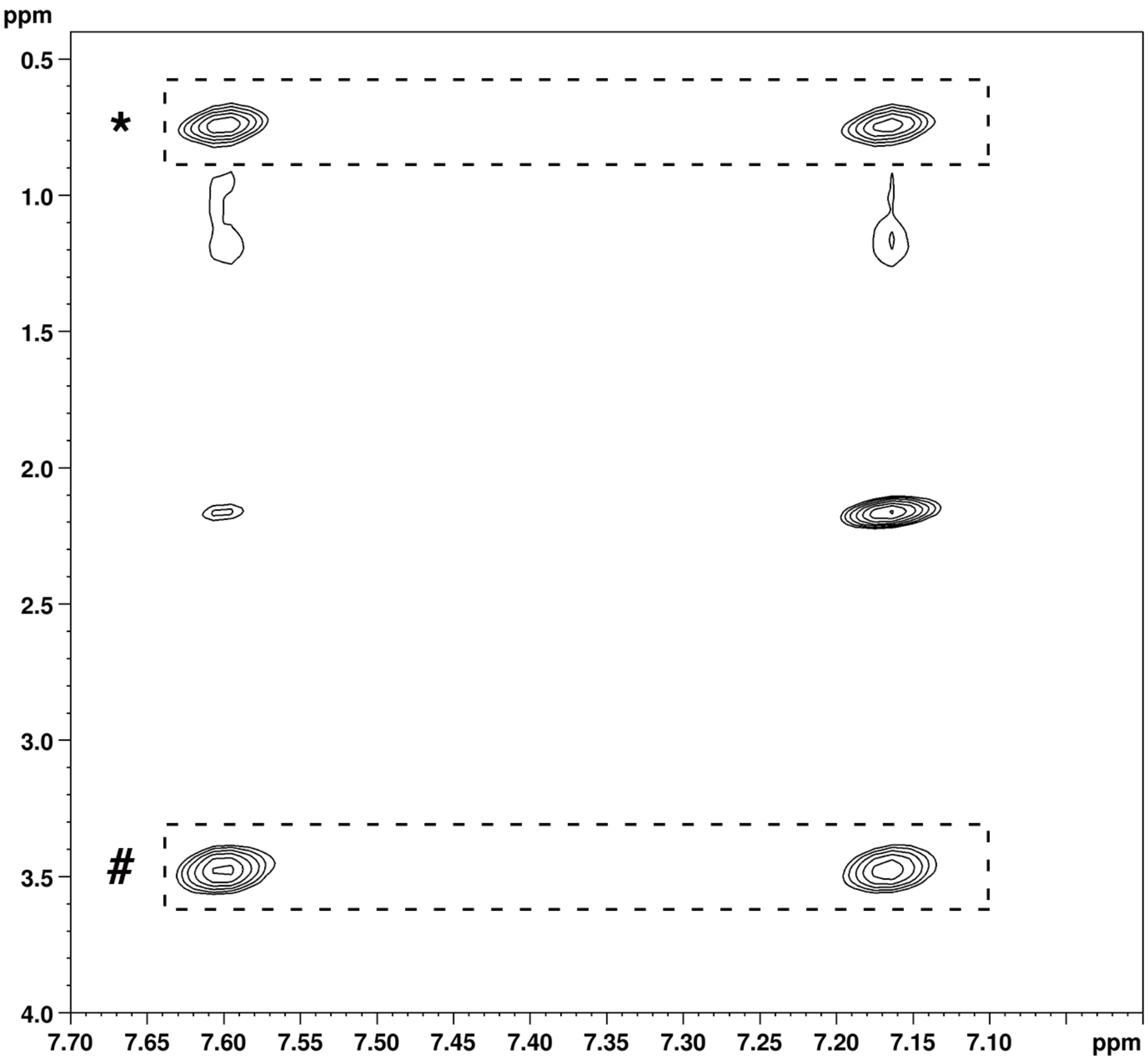

Figure S5. 2D ^{1}H-^{1}H NOESY spectrum of TosMIC (0.12 mmol) in in 2% *w/v* **TPGS-750-M** and K_2CO_3 (1 equiv.). NOE cross-peaks between TosMIC and **TPGS** micelle protons are highlighted with dashed boxes; in particular, those with PEG groups are marked with (#), and those with methyl signals of the micelle core with (*).

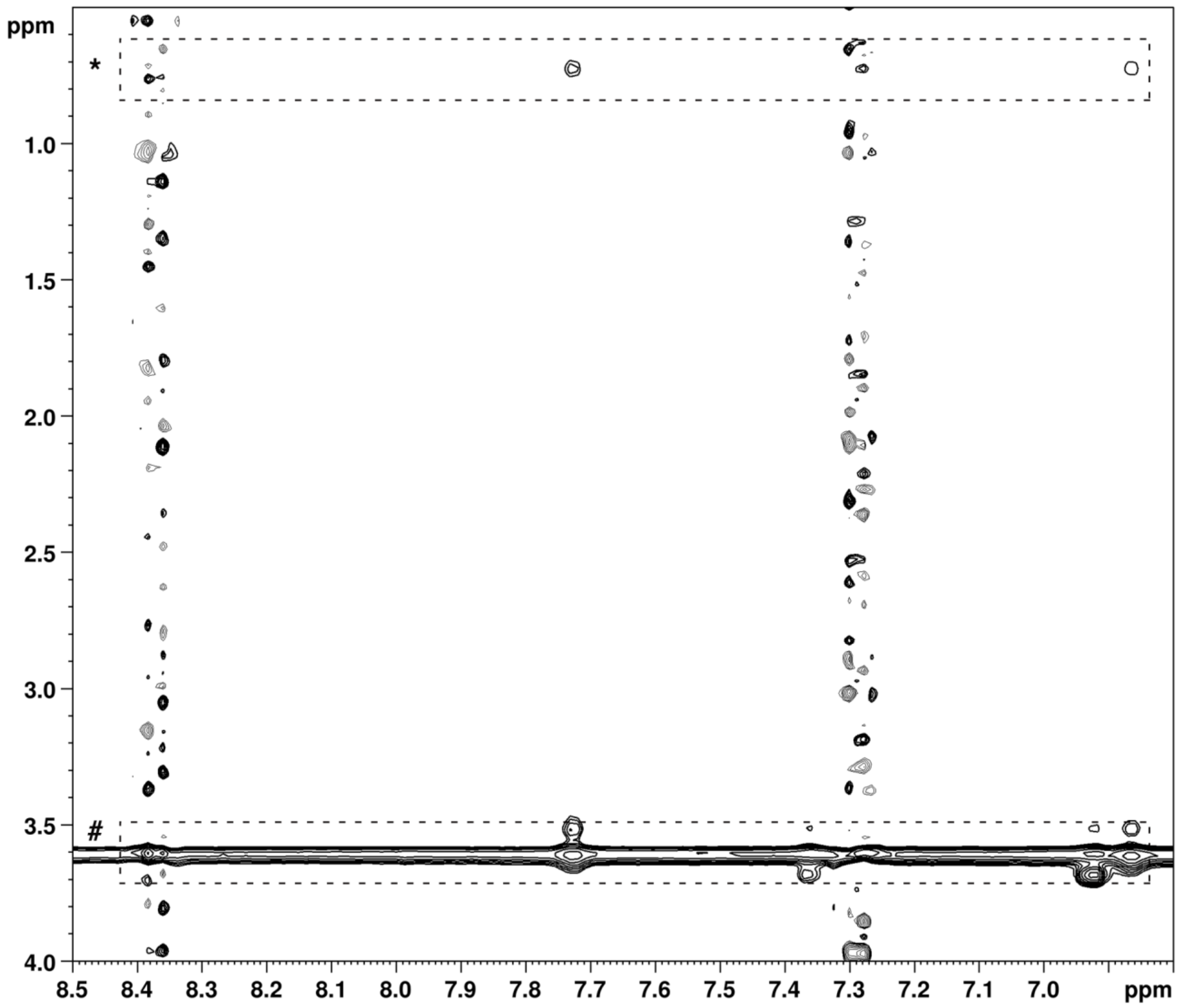

Figure S6. 2D ^{1}H-^{1}H NOESY spectrum of arylazo sulfone **1a** in in 2% *w/v* **TPGS-750-M**. NOE cross-peaks between arylazo sulfone **1a** and **TPGS** micelle protons are highlighted with dashed boxes; in particular, those with PEG groups are marked with (#), and those with methyl signals of the micelle core with (*).

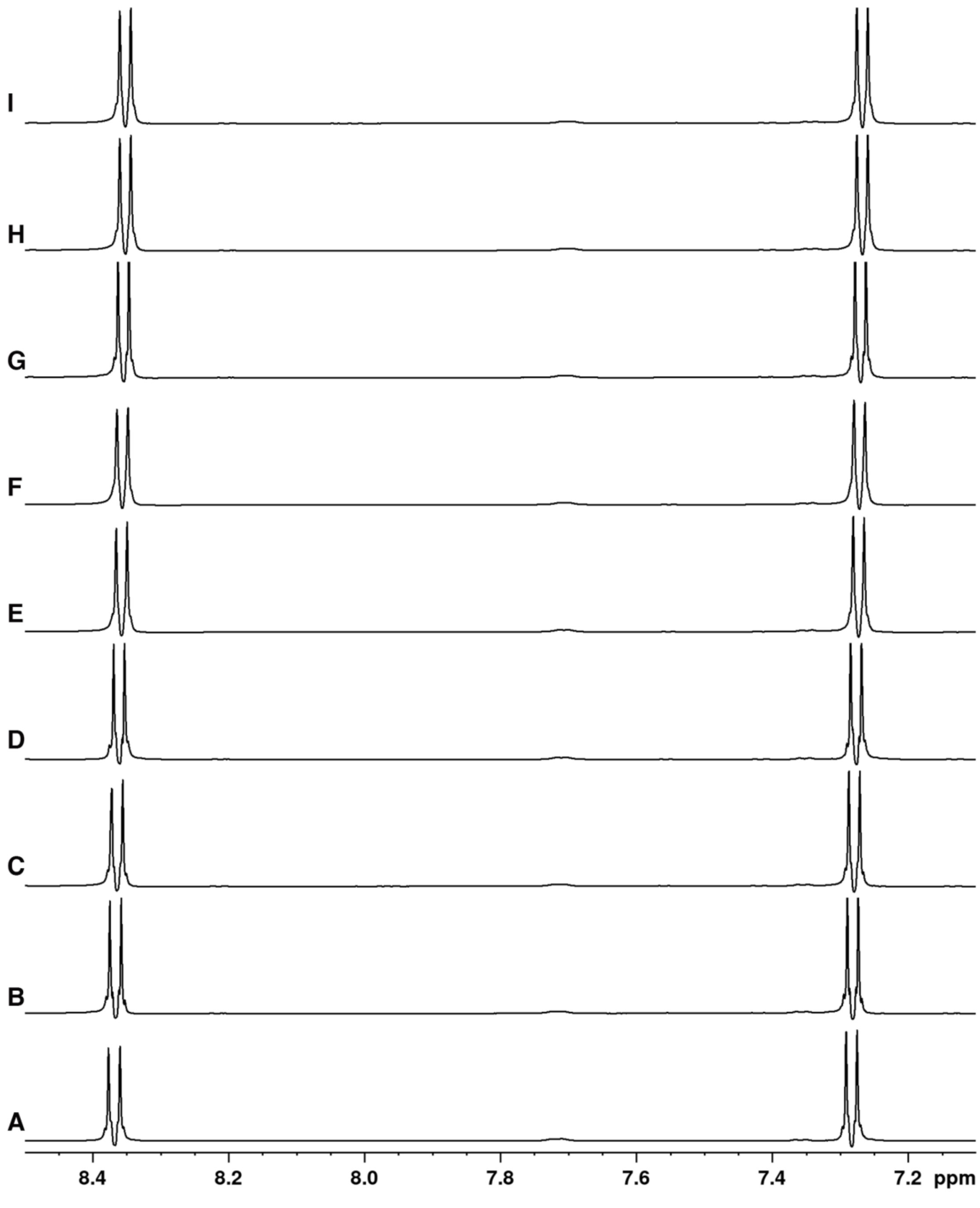

Figure S7. ^{1}H NMR titration of arylazo sulfone **1a** in 2% *w/v* **TPGS-750-M** micellar solution with increasing concentrations of $MnCl_2·4H_2O$. Spectra A–I correspond to Mn^{2+} concentrations of 0, 0.05, 0.1, 0.15, 0.2, 0.25, 0.3, 0.4, and 0.5 mM, respectively.

6.References

S1) Lian, C.; Yue, G.; Mao, J.; Liu, D.; Ding, Y.; Liu, Z.; Qiu, D.; Zhao, X.; Lu, K.; Fagnoni, M.; Protti, S. Visible-Light-Driven Synthesis of Arylstannanes from Arylazo Sulfones, *Org. Lett.* **2019**, *21*, 5187-5191.

S2) Lujan-Montelongo, J.A., Estevez, A.O. and Fleming, F.F. Alkyl Sulfinates: Formal Nucleophiles for Synthesizing TosMIC Analogs. *Eur. J. Org. Chem*. **2015**, *7*, 1602-1605.

S3) Nicchio, L.; Amin, H. I. M.; Genualdo, S.; Protti, S.; Fagnoni, M. Water Effect on the Photochemistry of Arylazo Sulfonates, *J. Org. Chem*. **2025**, *90*, 6726–6736.

7. Copy of ^{1}H and $^{13}C\{^{1}H\}$ NMR spectra.

1-bromo-4-((isocyanomethyl)sulfonyl)benzene (2d). ^{1}H NMR (400 MHz, $CDCl_3$)

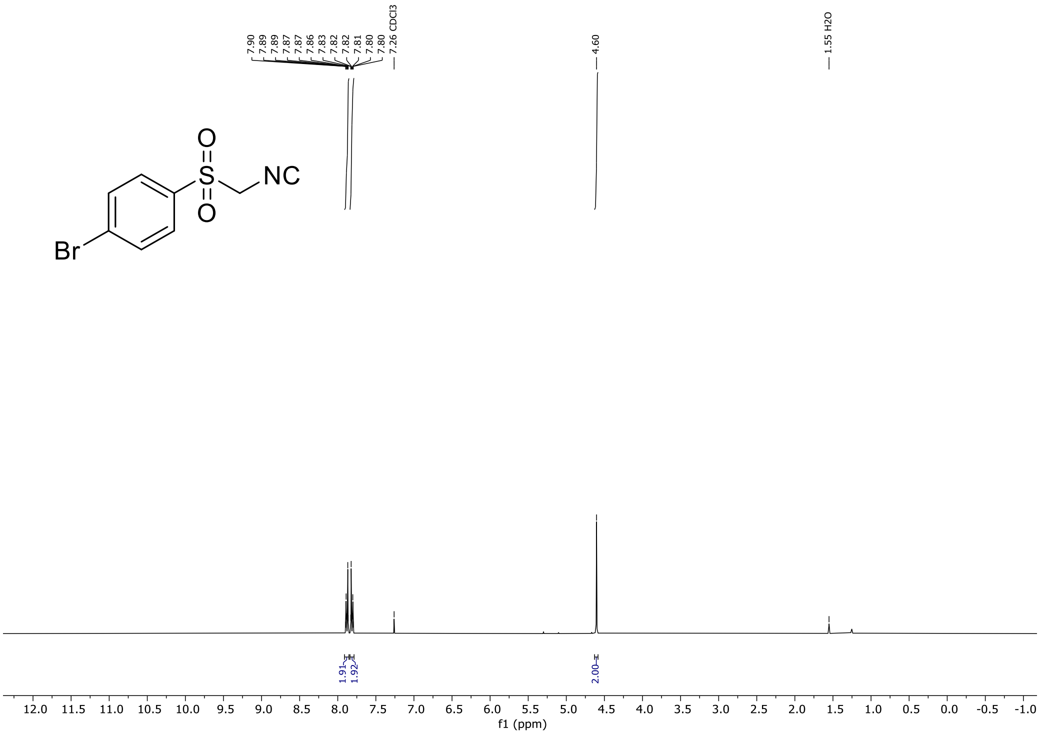

1-bromo-4-((isocyanomethyl)sulfonyl)benzene (2d). $^{13}C\{^{1}H\}$ NMR (101 MHz, $CDCl_3$)

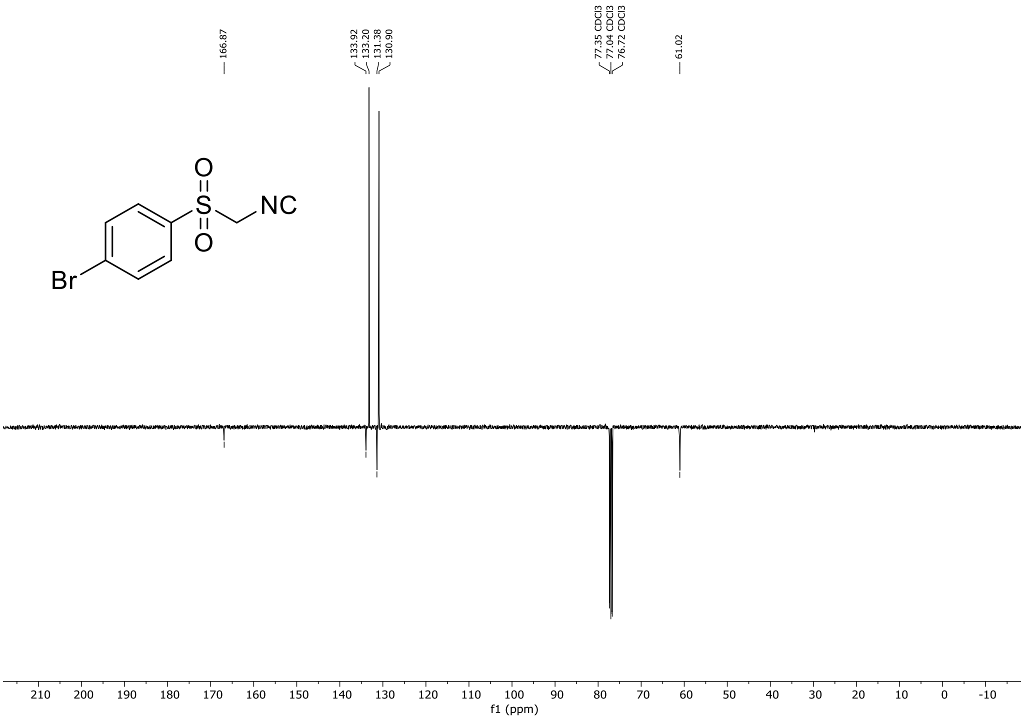

1-(4-methoxyphenyl)-3-tosyl-1*H*-1,2,4-triazole (3a). ^{1}H NMR (400 MHz, $CDCl_3$)

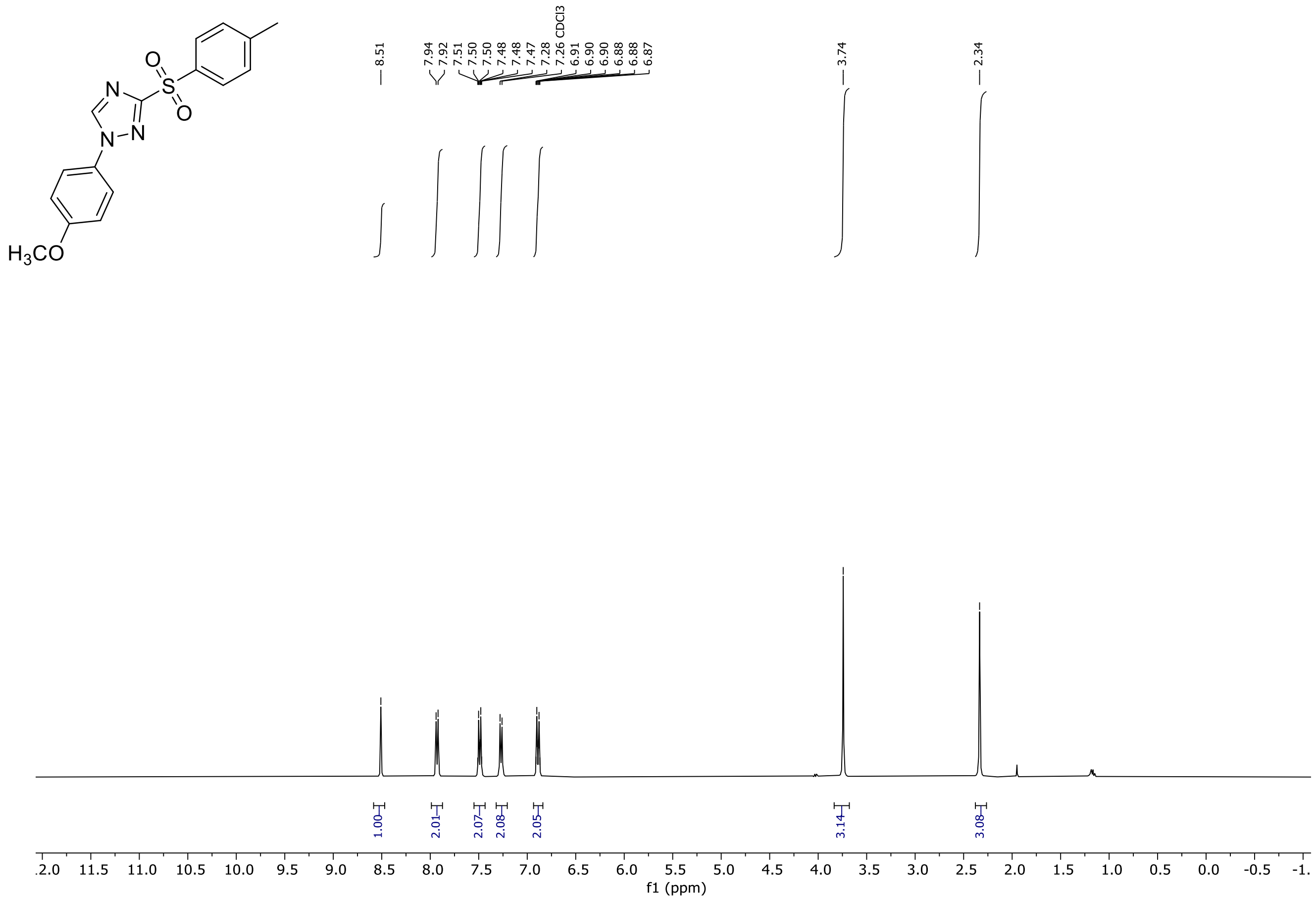

1-(4-methoxyphenyl)-3-tosyl-1*H*-1,2,4-triazole (3a). ^{13}C{^{1}H} NMR (101 MHz, $CDCl_3$)

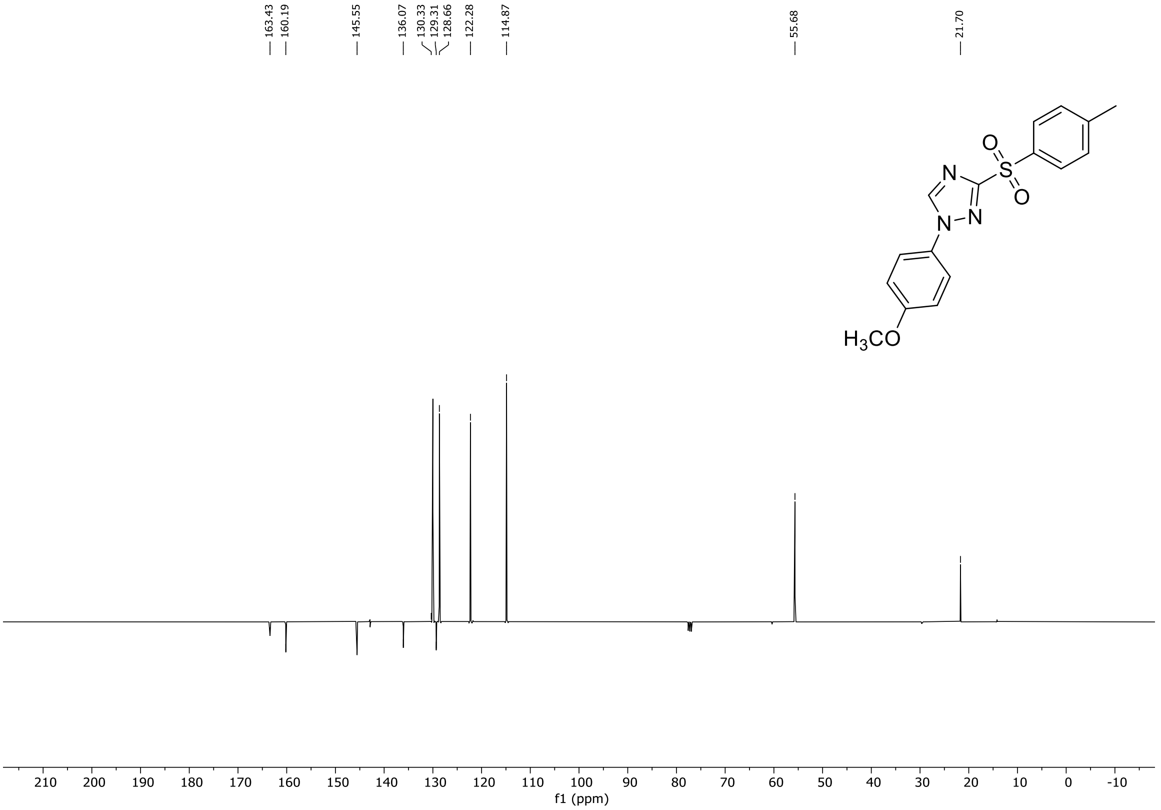

1-(4-methoxyphenyl)-5-(naphthalen-2-ylsulfonyl)-1*H*-1,2,4-triazole (3b). ^{1}H NMR (400 MHz, $CDCl_3$)

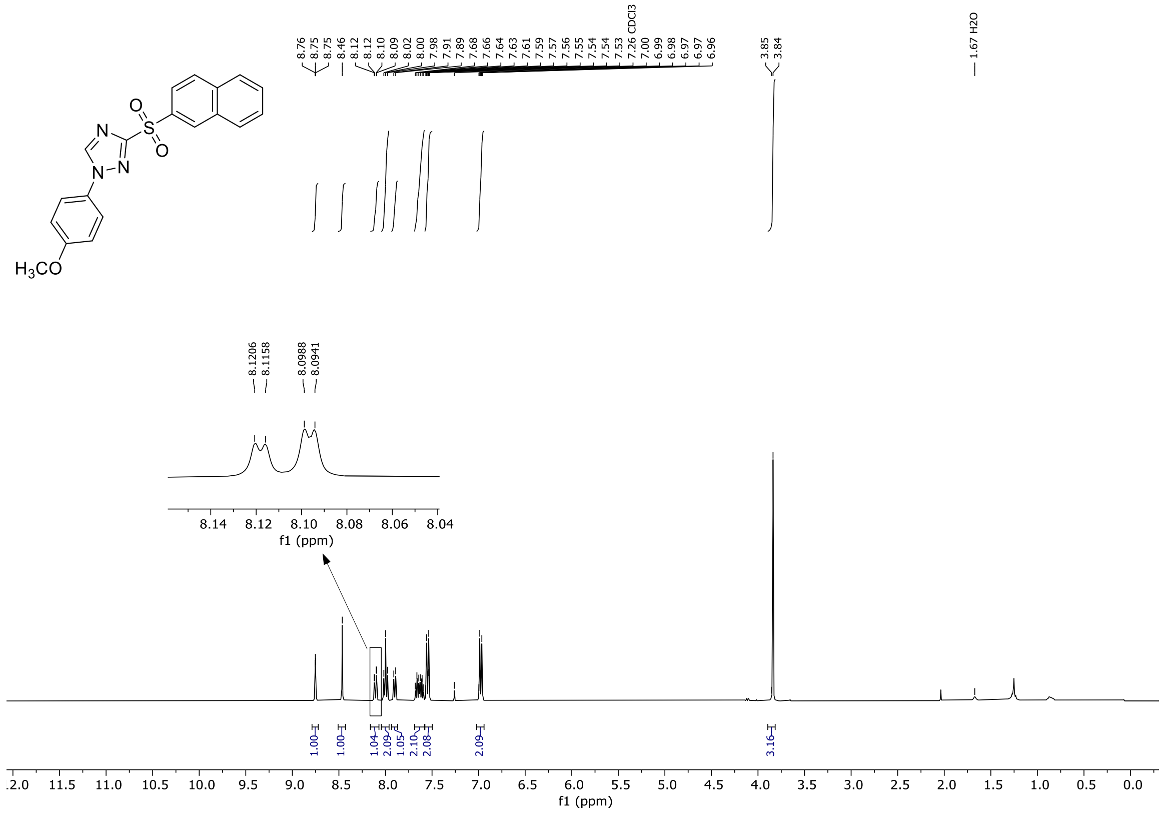

1-(4-methoxyphenyl)-5-(naphthalen-2-ylsulfonyl)-1*H*-1,2,4-triazole (3b). $^{13}C\{^{1}H\}$ NMR (101 MHz, $CDCl_3$)

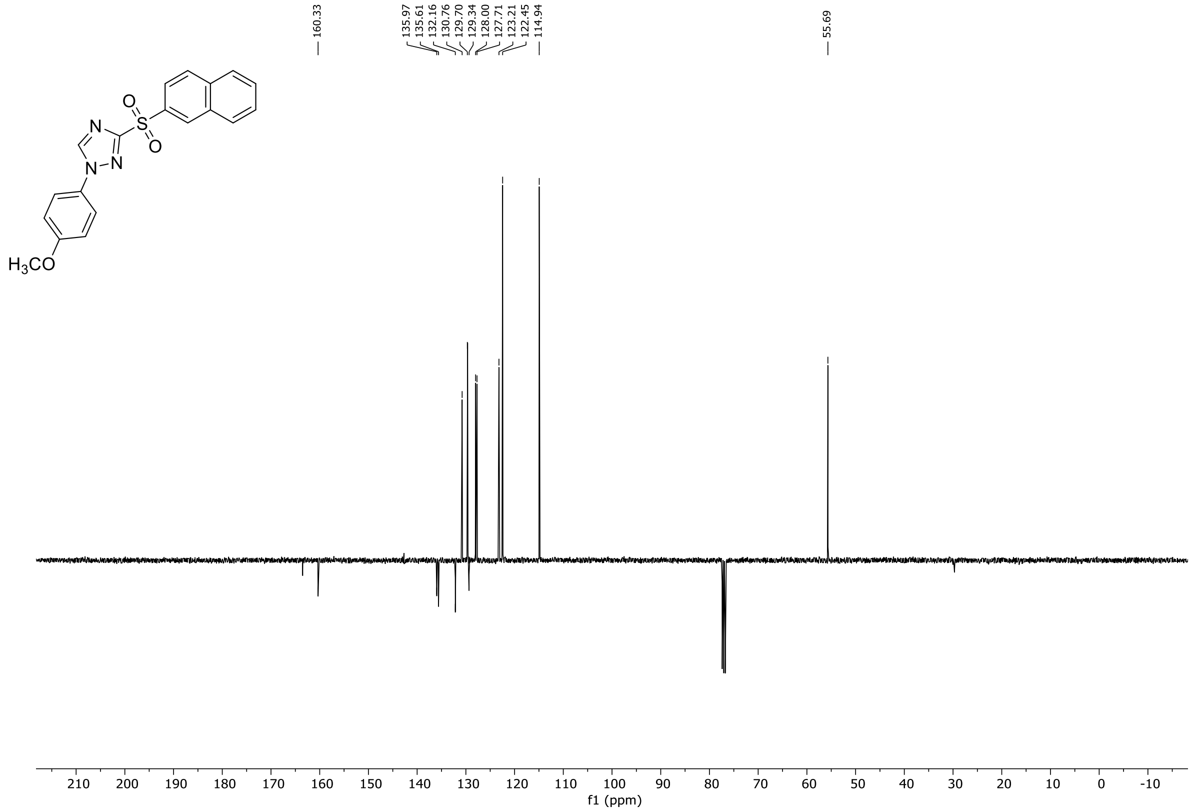

1-(4-chlorophenyl)-5-(naphthalen-2-ylsulfonyl)-1*H*-1,2,4-triazole (3c). ^{1}H NMR (400 MHz, $CDCl_3$)

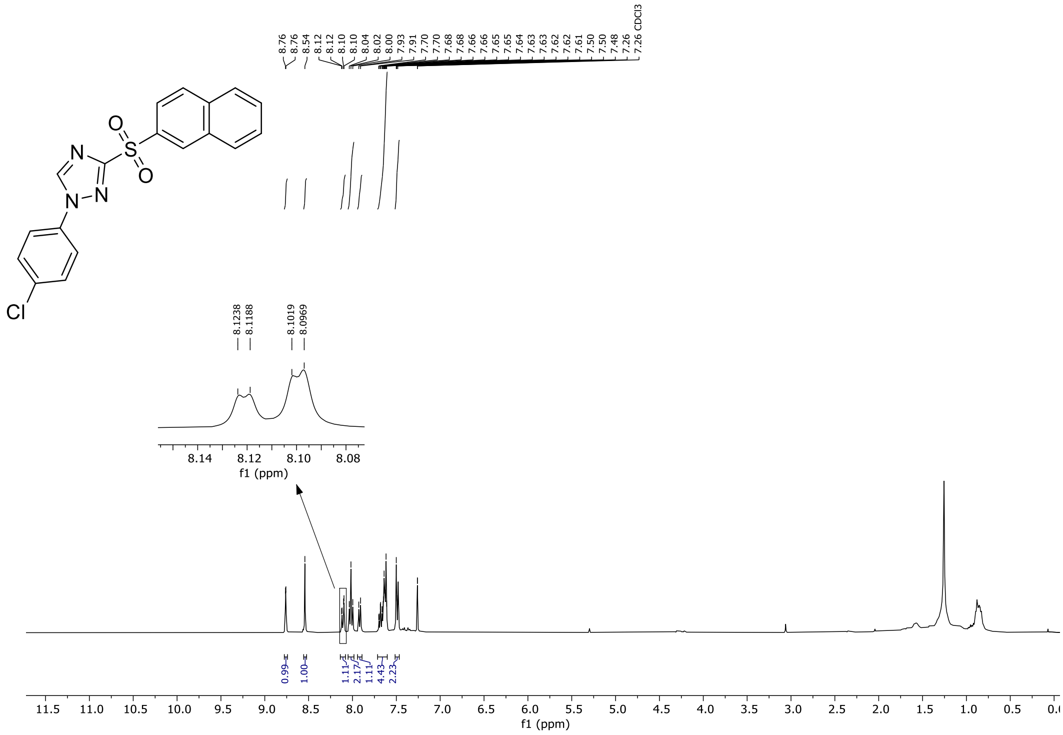

1-(4-chlorophenyl)-5-(naphthalen-2-ylsulfonyl)-1*H*-1,2,4-triazole (3c). ^{13}C{^{1}H} NMR (101 MHz, $CDCl_3$)

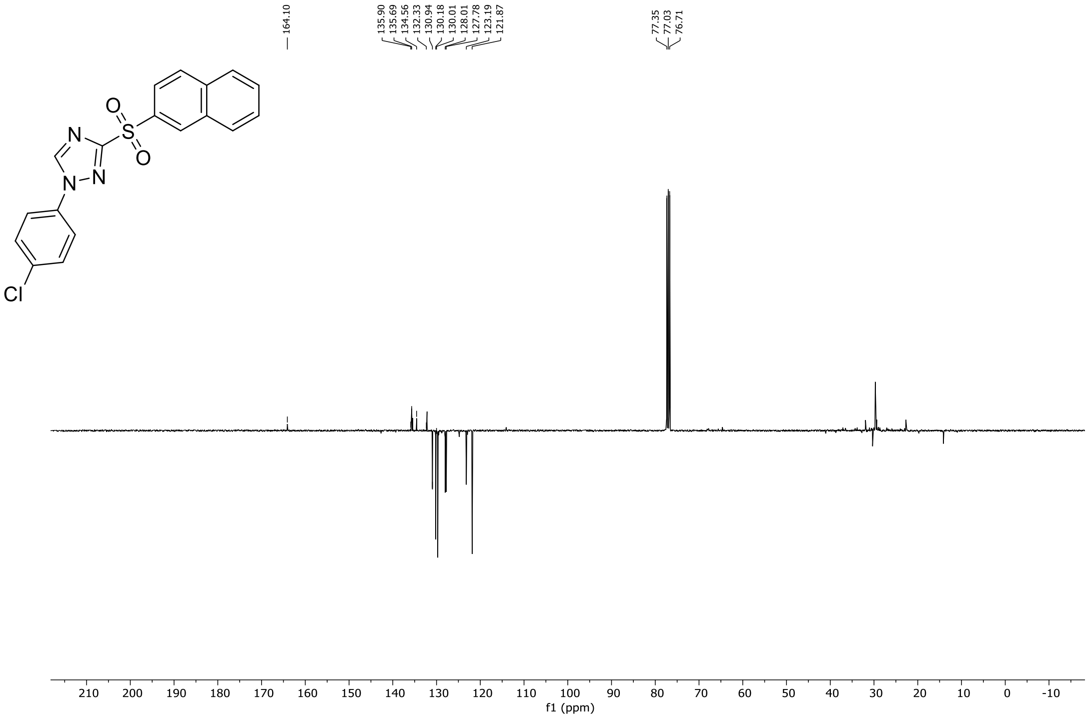

5-((4-chlorophenyl)sulfonyl)-1-(4-methoxyphenyl)-1*H*-1,2,4-triazole (3d). ^{1}H NMR (400 MHz, $CDCl_3$)

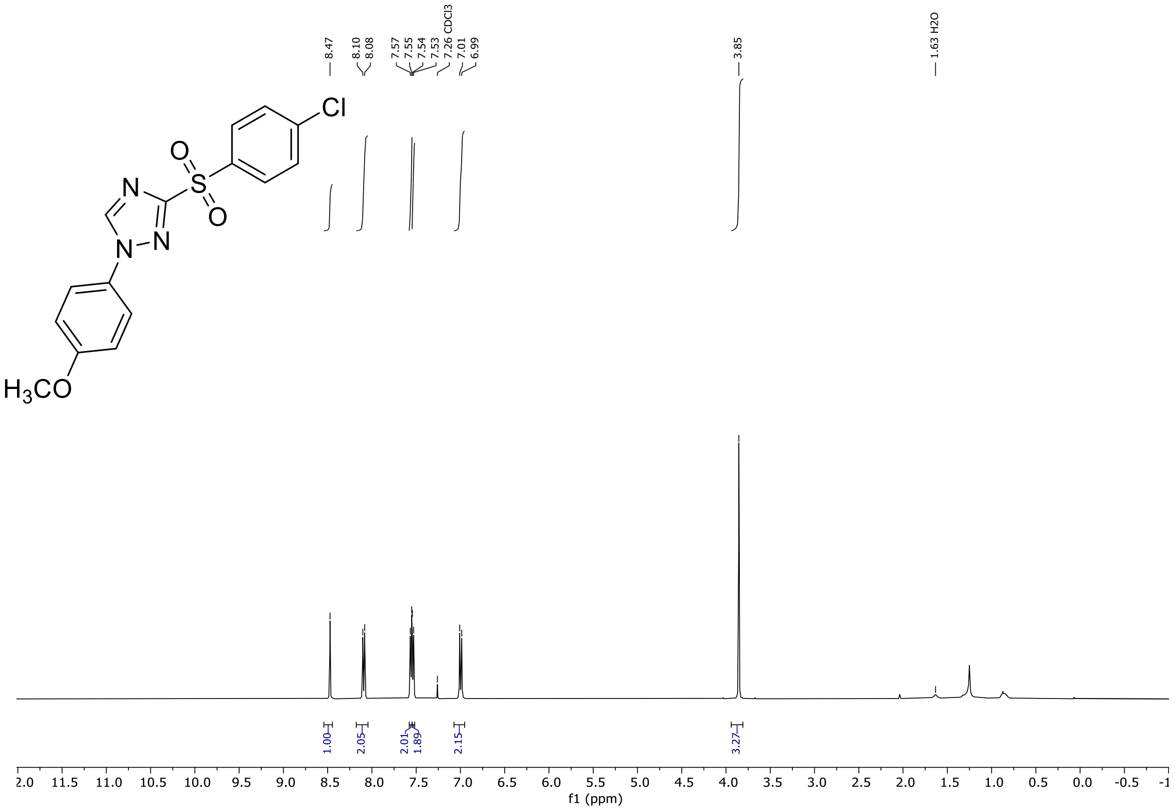

5-((4-chlorophenyl)sulfonyl)-1-(4-methoxyphenyl)-1*H*-1,2,4-triazole (3d). $^{13}C\{^{1}H\}$ NMR (101 MHz, $CDCl_3$)

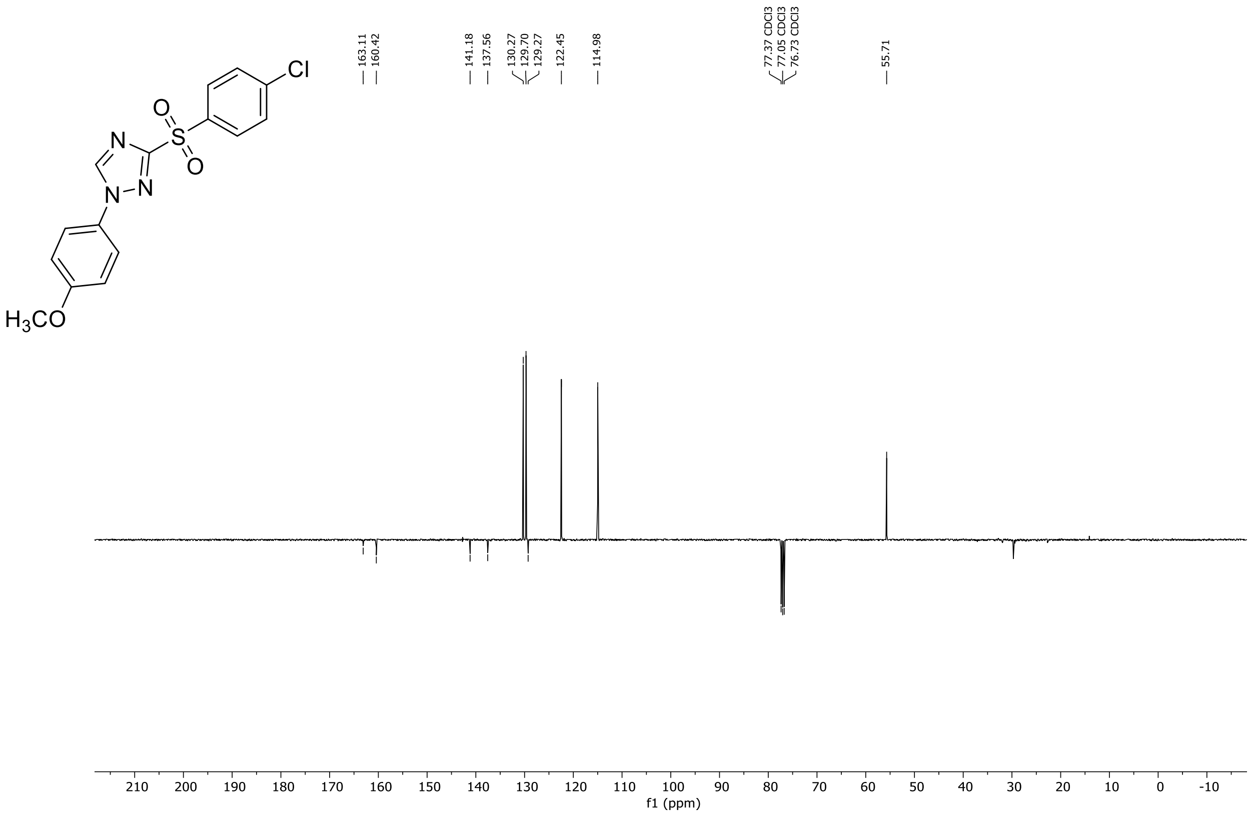

1-(4-chlorophenyl)-5-((4-chlorophenyl)sulfonyl)-1*H*-1,2,4-triazole (3e). ^{1}H NMR (400 MHz, $CDCl_3$)

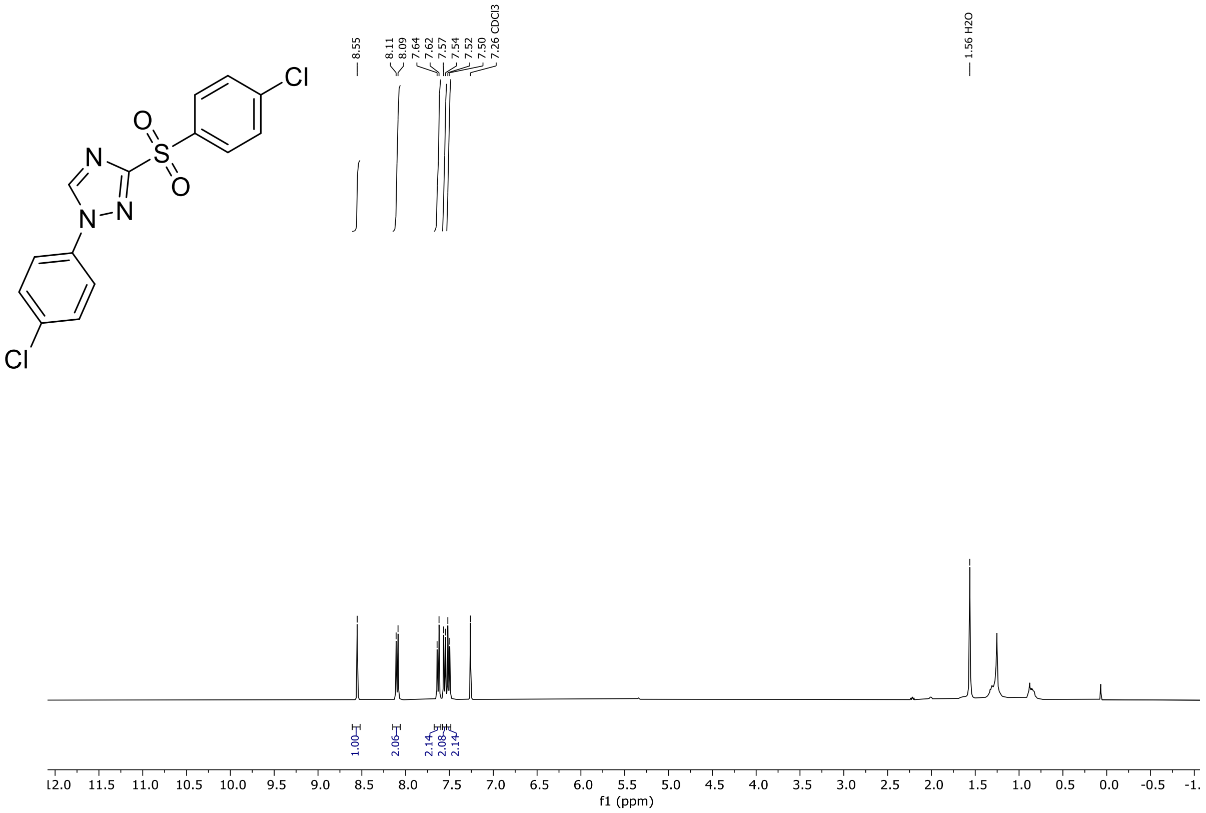

1-(4-chlorophenyl)-5-((4-chlorophenyl)sulfonyl)-1*H*-1,2,4-triazole (3e). $^{13}C\{^{1}H\}$ NMR (101 MHz, $CDCl_3$)

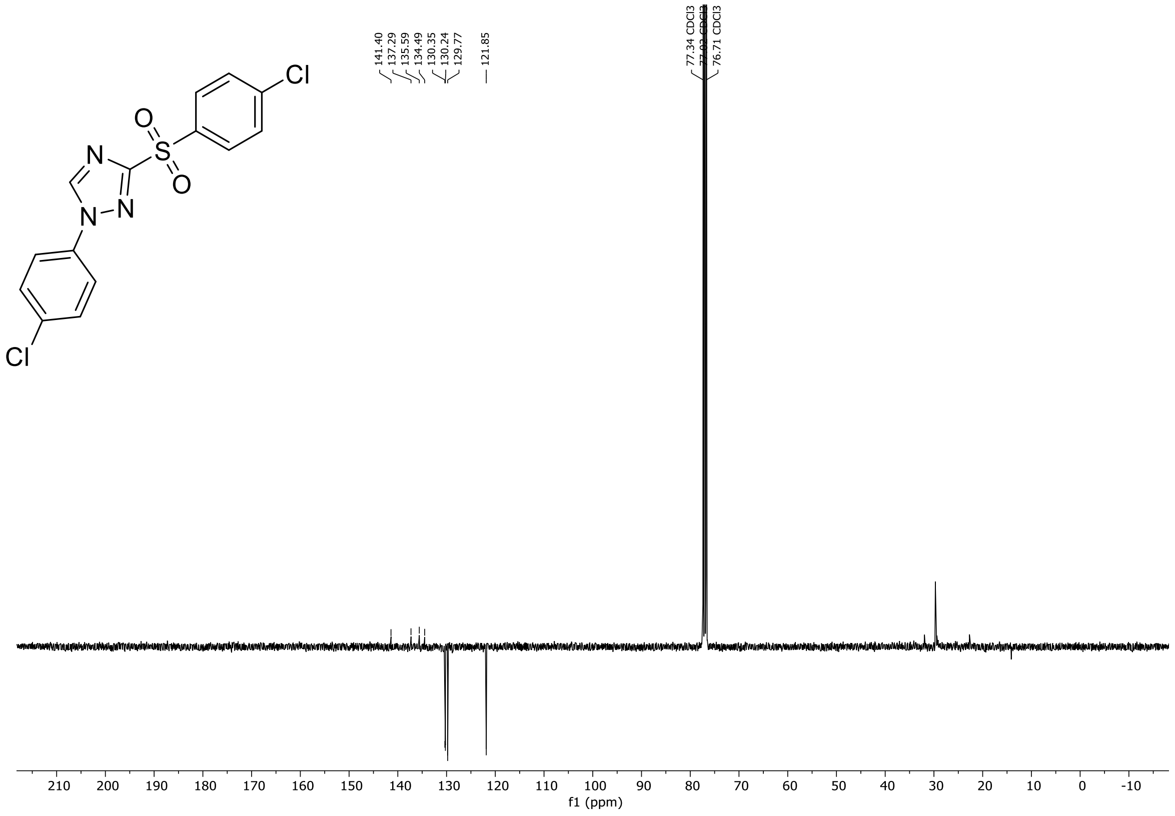

5-((4-chlorophenyl)sulfonyl)-1-(*p*-tolyl)-1*H*-1,2,4-triazole (3f). ^{1}H NMR (400 MHz, $CDCl_3$)

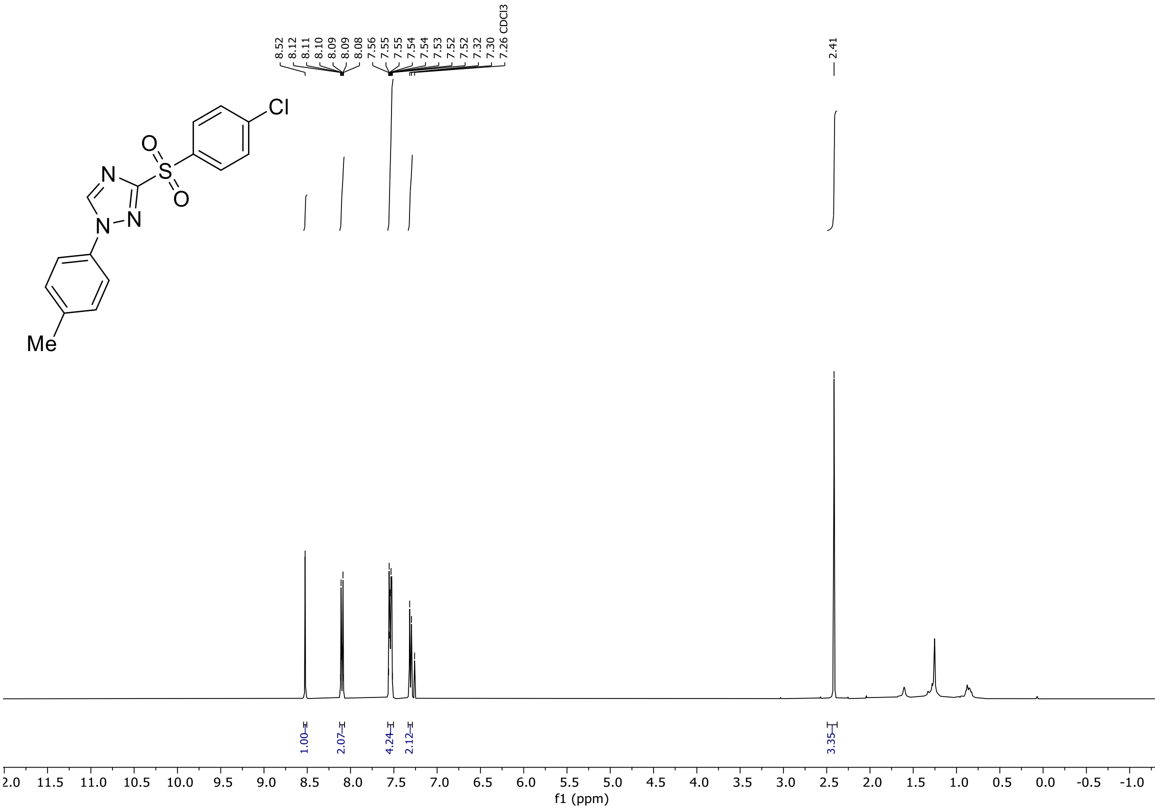

5-((4-chlorophenyl)sulfonyl)-1-(*p*-tolyl)-1*H*-1,2,4-triazole (3f). $^{13}C\{^{1}H\}$ NMR (101 MHz, $CDCl_3$)

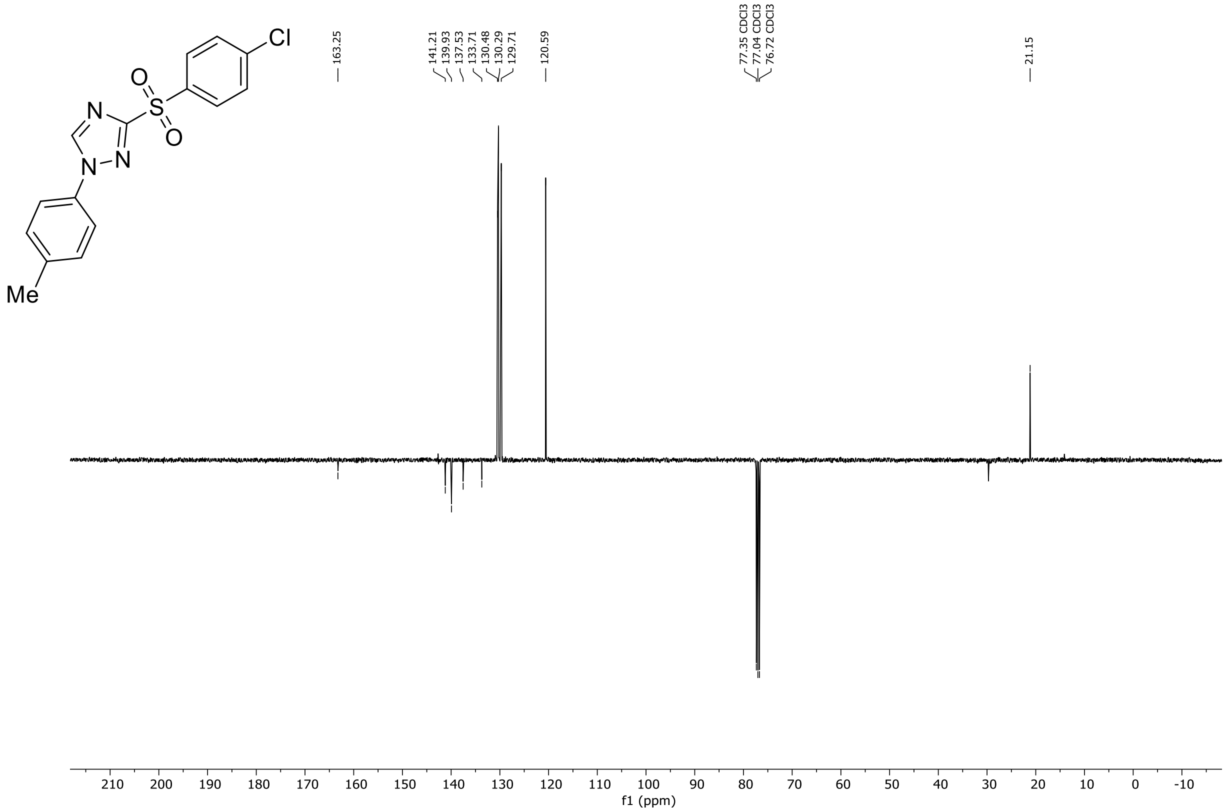

5-((4-bromophenyl)sulfonyl)-1-(4-methoxyphenyl)-1*H*-1,2,4-triazole (3g). ^{1}H NMR (400 MHz, $CDCl_3$)

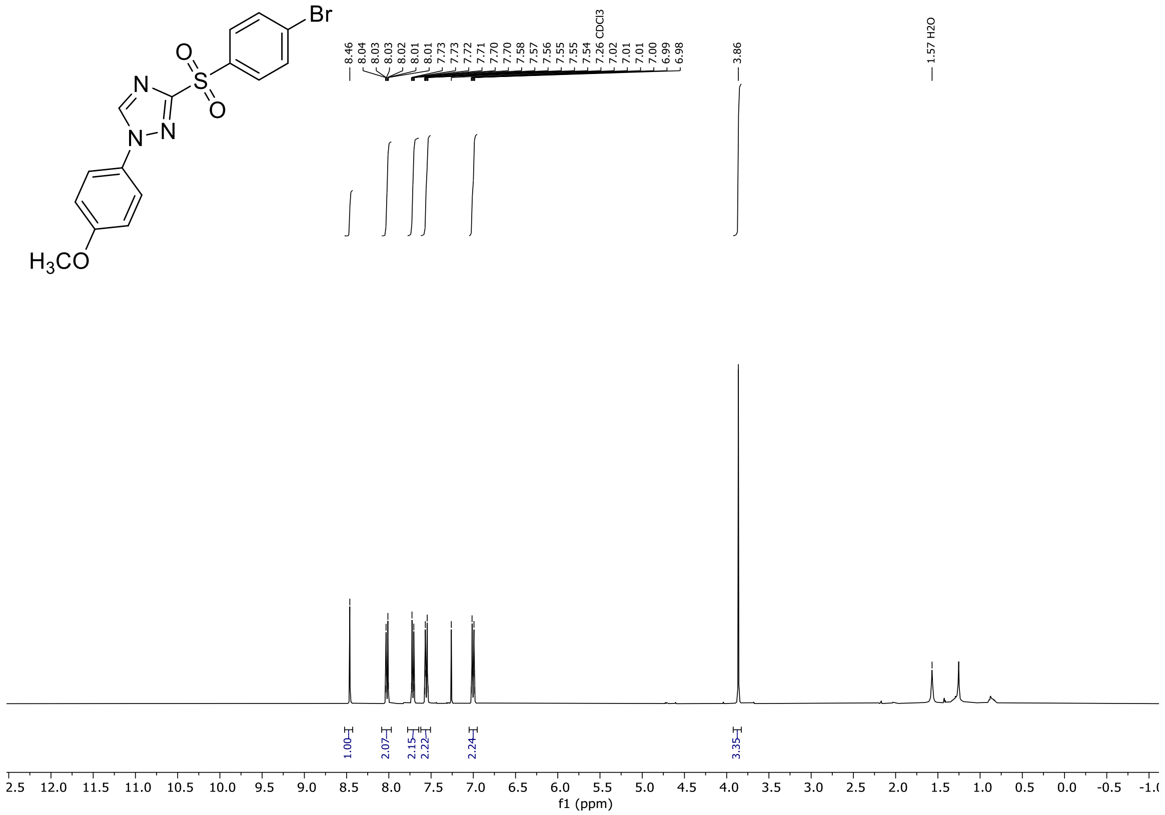

5-((4-bromophenyl)sulfonyl)-1-(4-methoxyphenyl)-1*H*-1,2,4-triazole (3g). $^{13}C\{^{1}H\}$ NMR (101 MHz, $CDCl_3$)

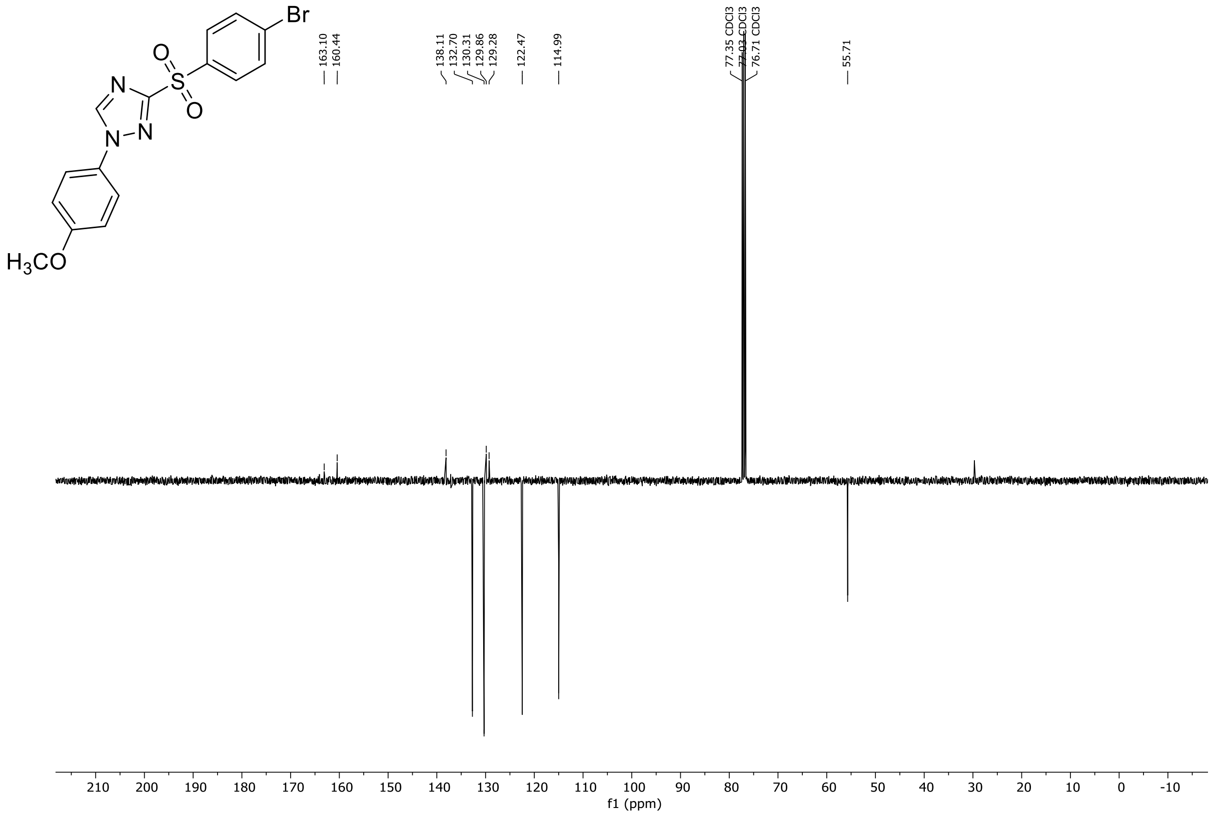

5-((4-bromophenyl)sulfonyl)-1-(*p*-tolyl)-1*H*-1,2,4-triazole (3h). ^{1}H NMR (400 MHz, $CDCl_3$)

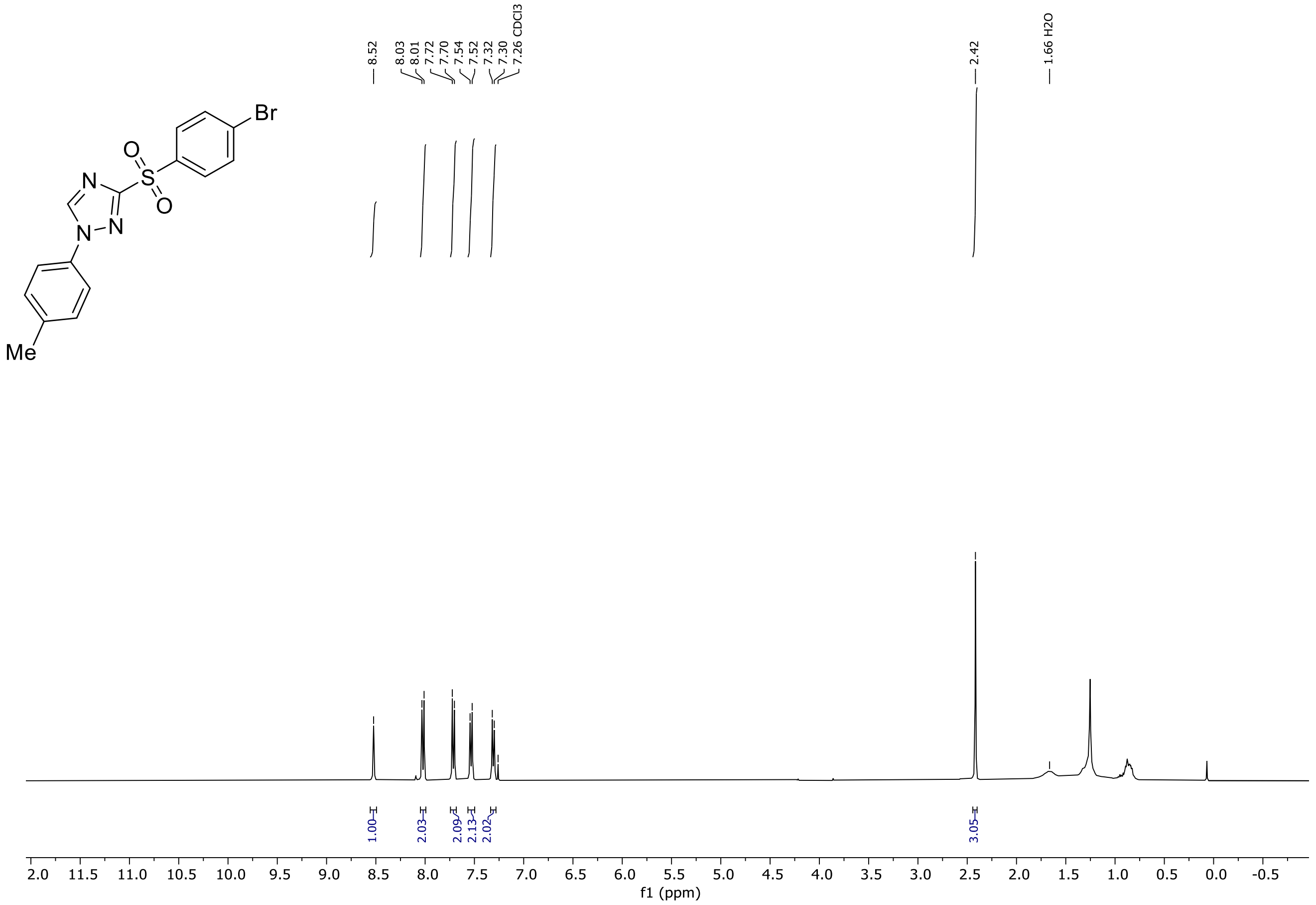

5-((4-bromophenyl)sulfonyl)-1-(*p*-tolyl)-1*H*-1,2,4-triazole (3h). $^{13}C\{^{1}H\}$ NMR (101 MHz, $CDCl_3$)

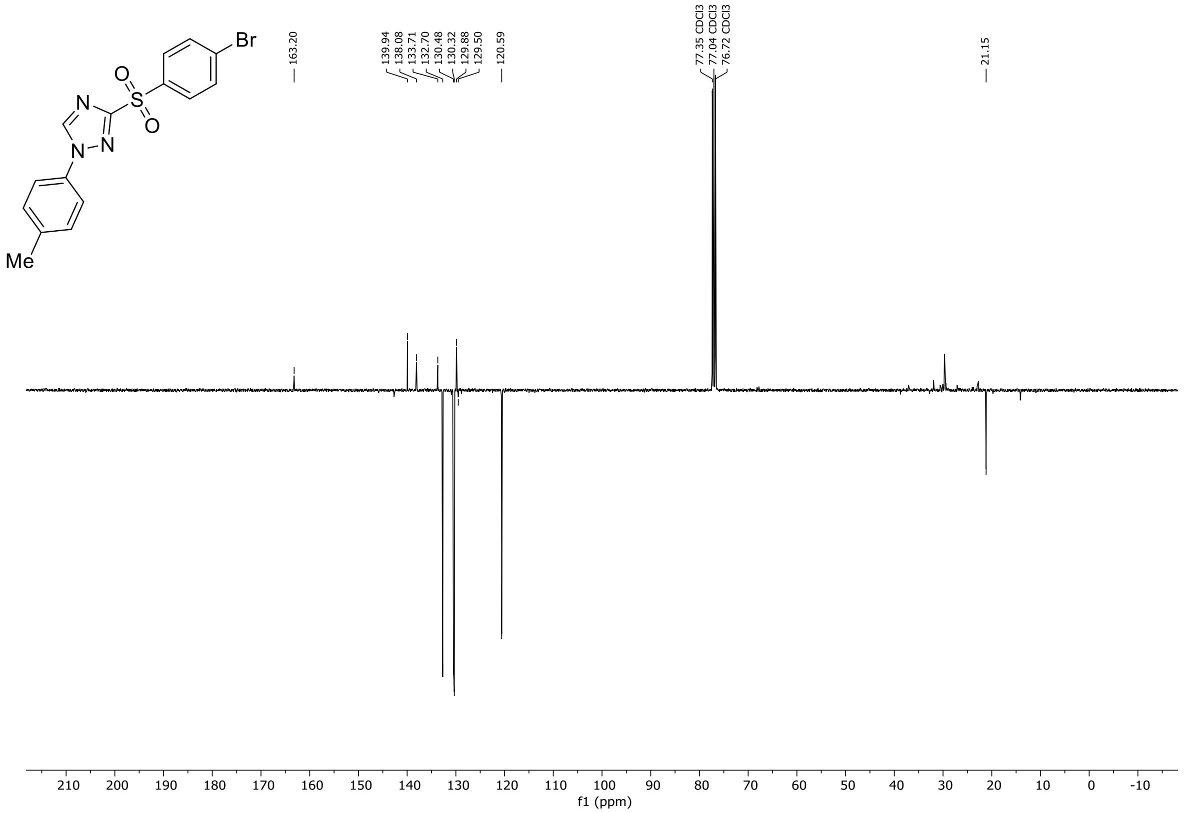

5-((4-bromophenyl)sulfonyl)-1-(4-chlorophenyl)-1*H*-1,2,4-triazole (3i). ^{1}H NMR (400 MHz, $CDCl_3$)

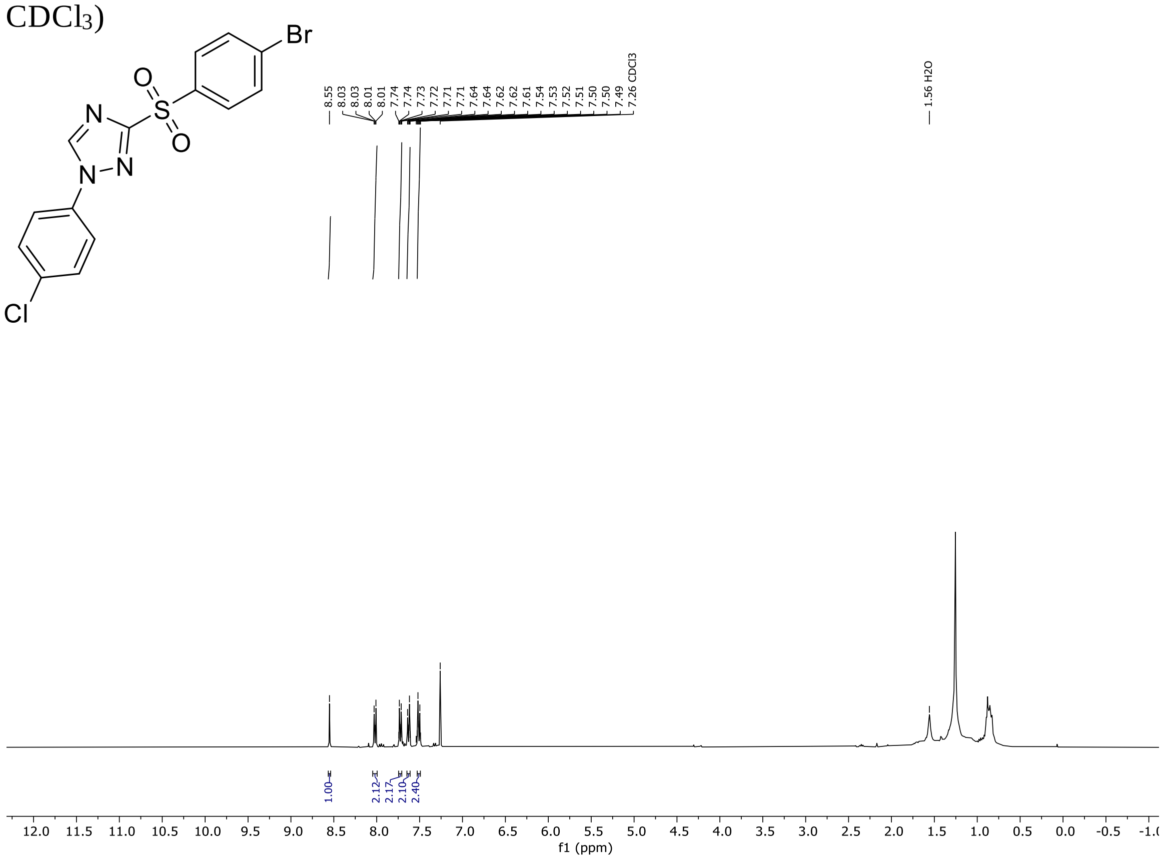

5-((4-bromophenyl)sulfonyl)-1-(4-chlorophenyl)-1*H*-1,2,4-triazole (3i). ^{13}C{^{1}H} NMR (101 MHz, $CDCl_3$)

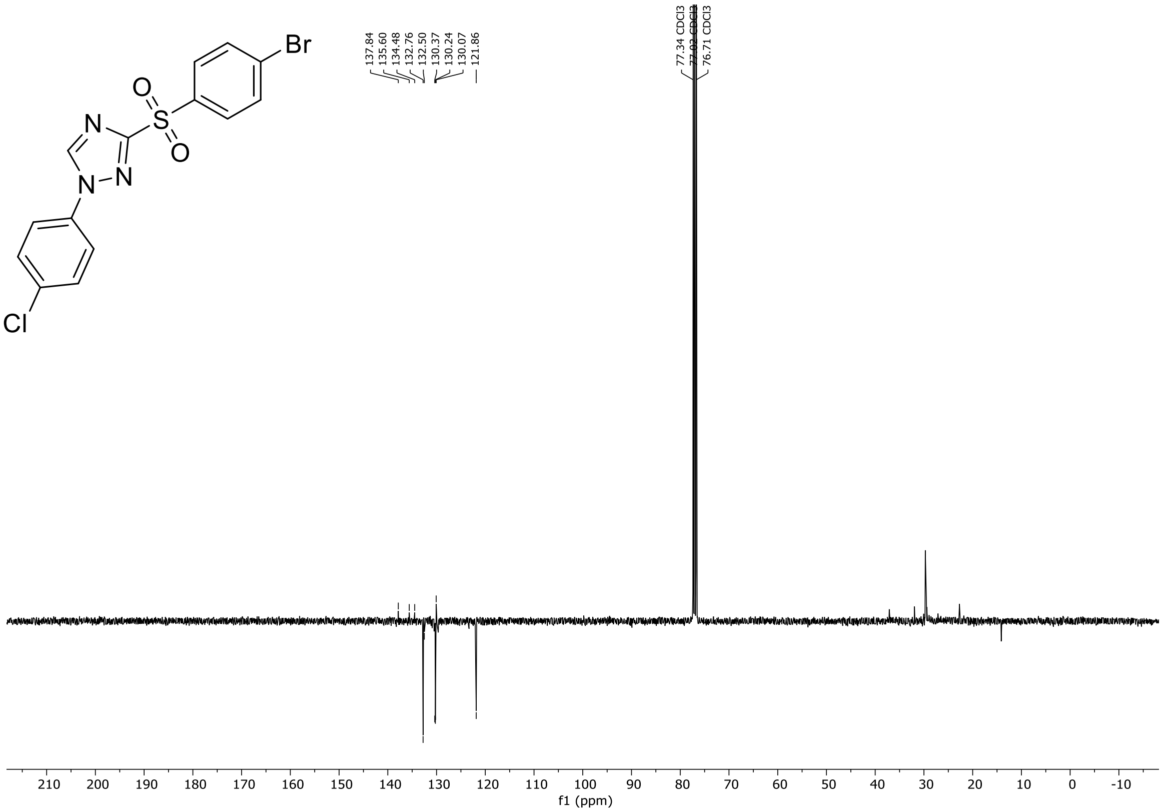

Methyl 1-(4-methoxyphenyl)-1*H*-1,2,4-triazole-5-carboxylate (3j). ^{1}H NMR (400 MHz, $CDCl_3$)

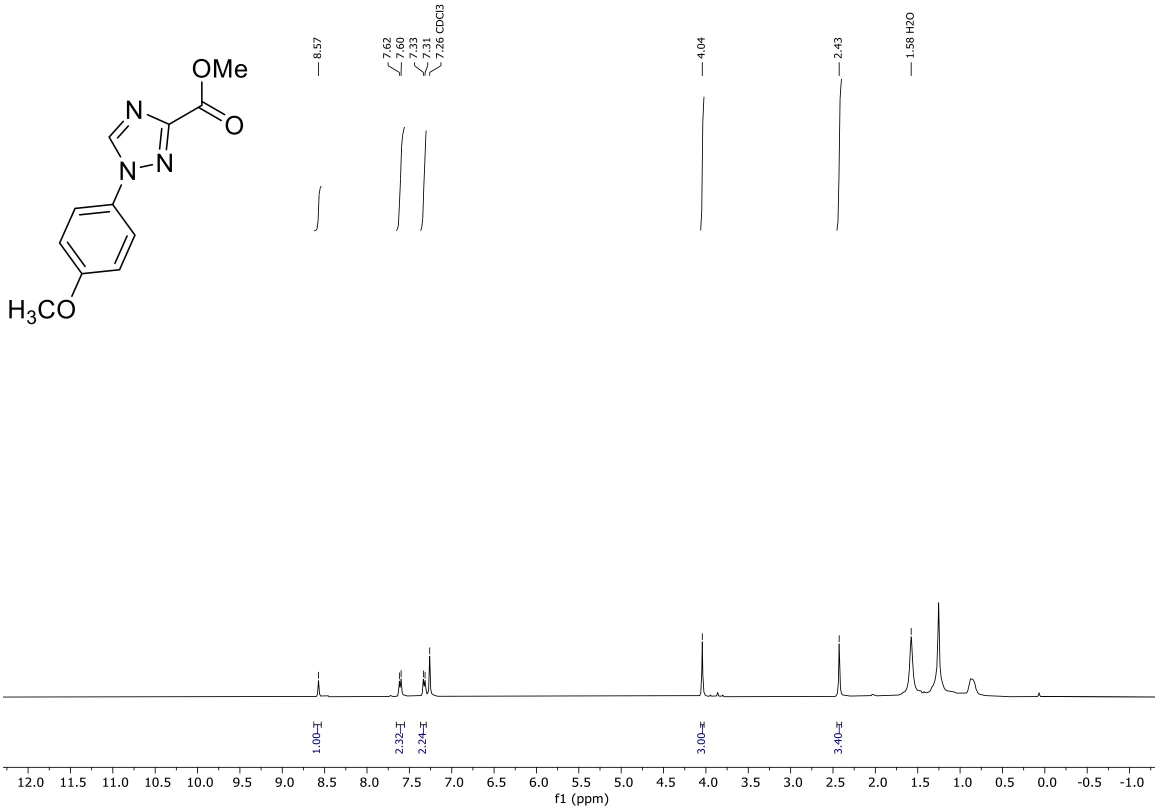

Methyl 1-(4-methoxyphenyl)-1*H*-1,2,4-triazole-5-carboxylate (3j). $^{13}C\{^{1}H\}$ NMR (101 MHz, $CDCl_3$)

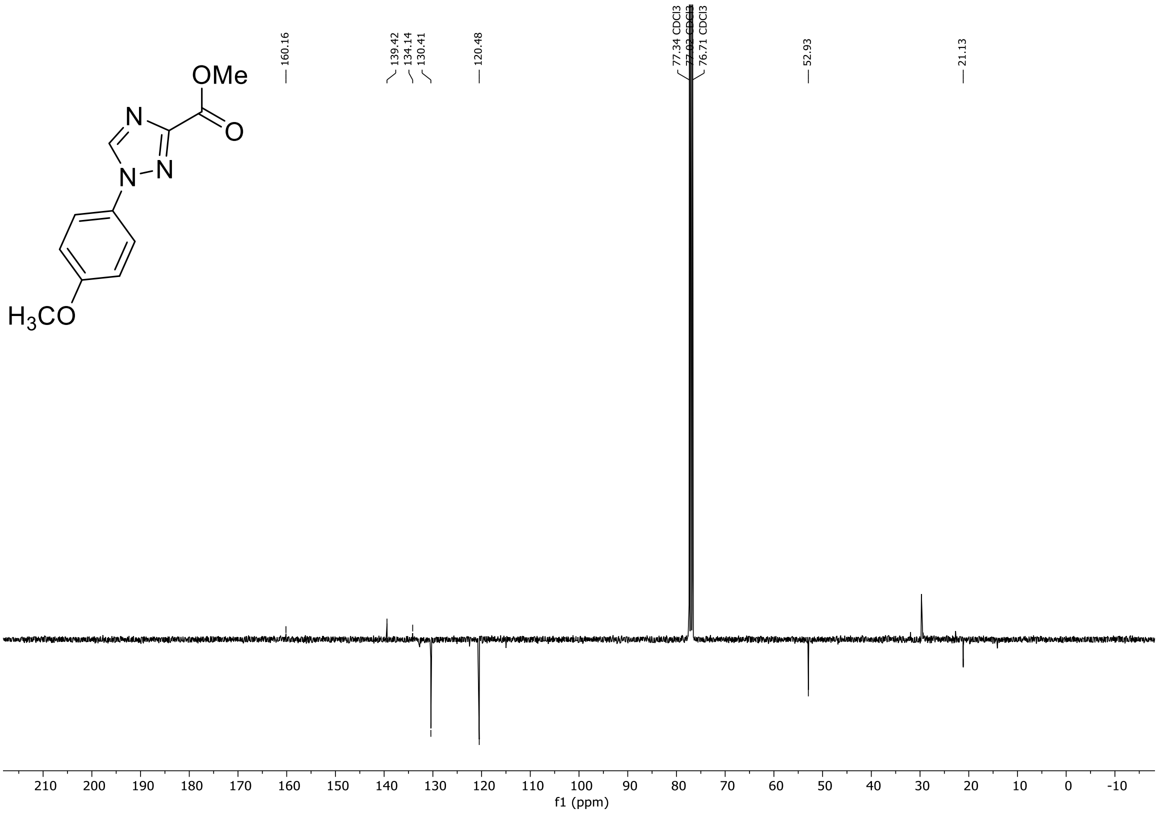

***N*,*N*-dimethyl-4-(3-tosyl-1*H*-1,2,4-triazol-1-yl)aniline (3k).** ^{1}H NMR (300 MHz, $CDCl_3$)

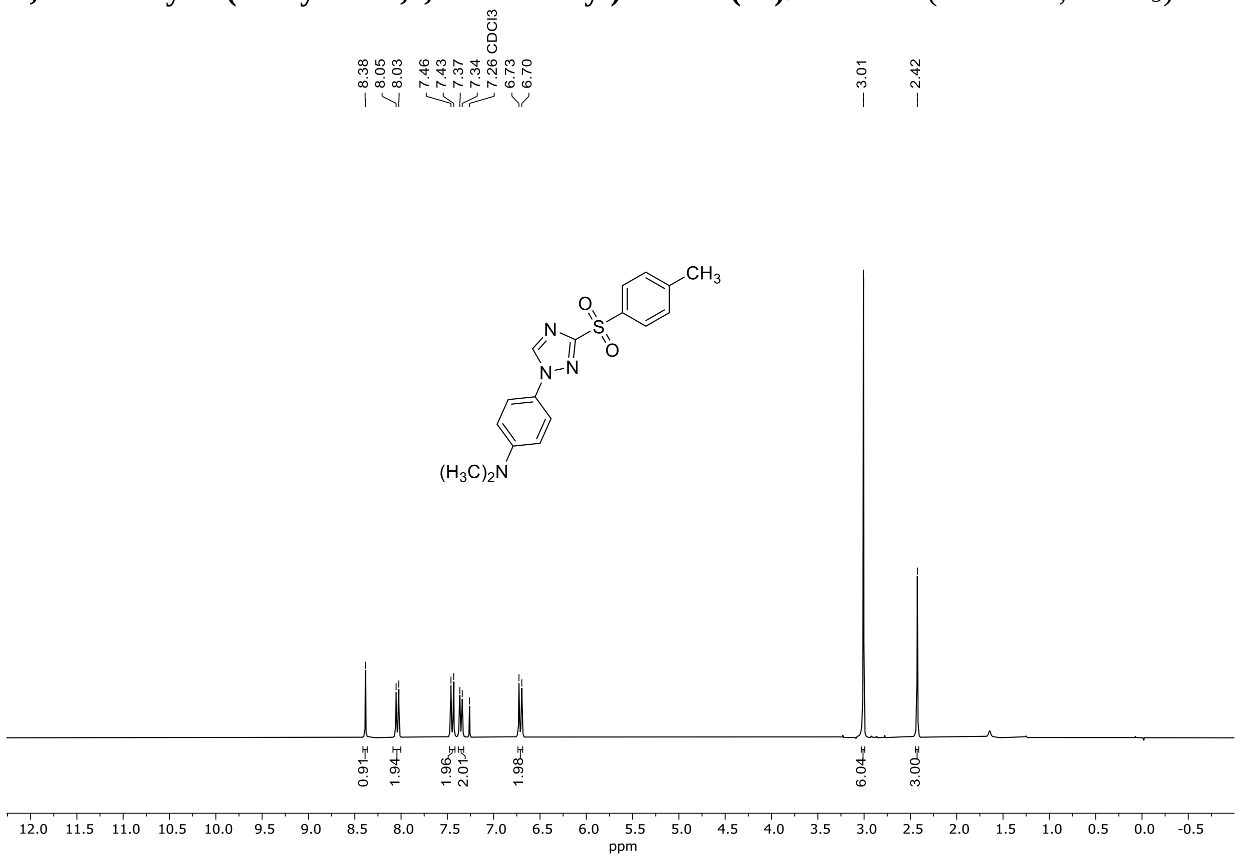

***N*,*N*-dimethyl-4-(3-tosyl-1*H*-1,2,4-triazol-1-yl)aniline (3k).** ^{13}C{^{1}H} NMR (75 MHz, $CDCl_3$)

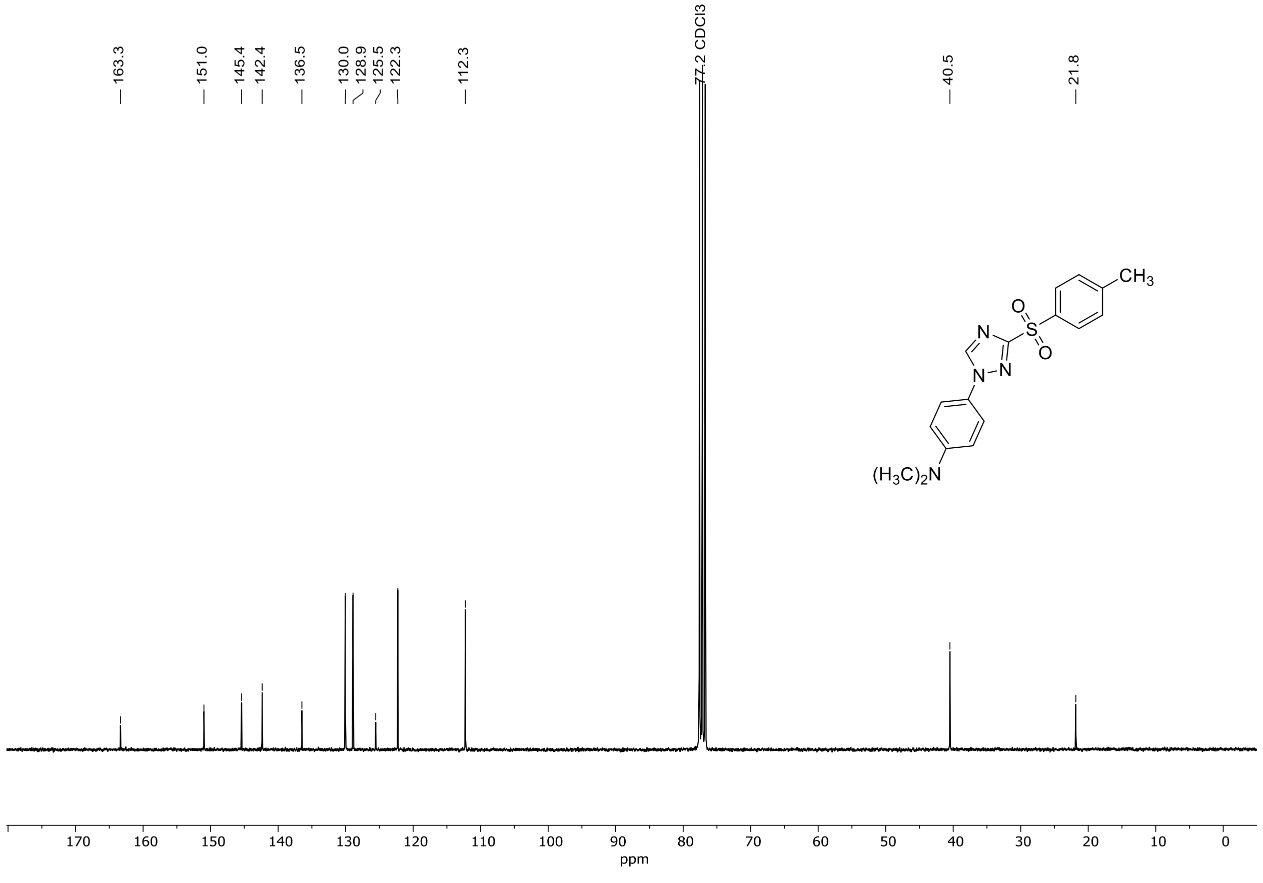

***N*-(3-(5-tosyl-1*H*-1,2,4-triazol-1-yl)phenyl)acetamide (3l).** ^{1}H NMR (300 MHz, $CDCl_3$)

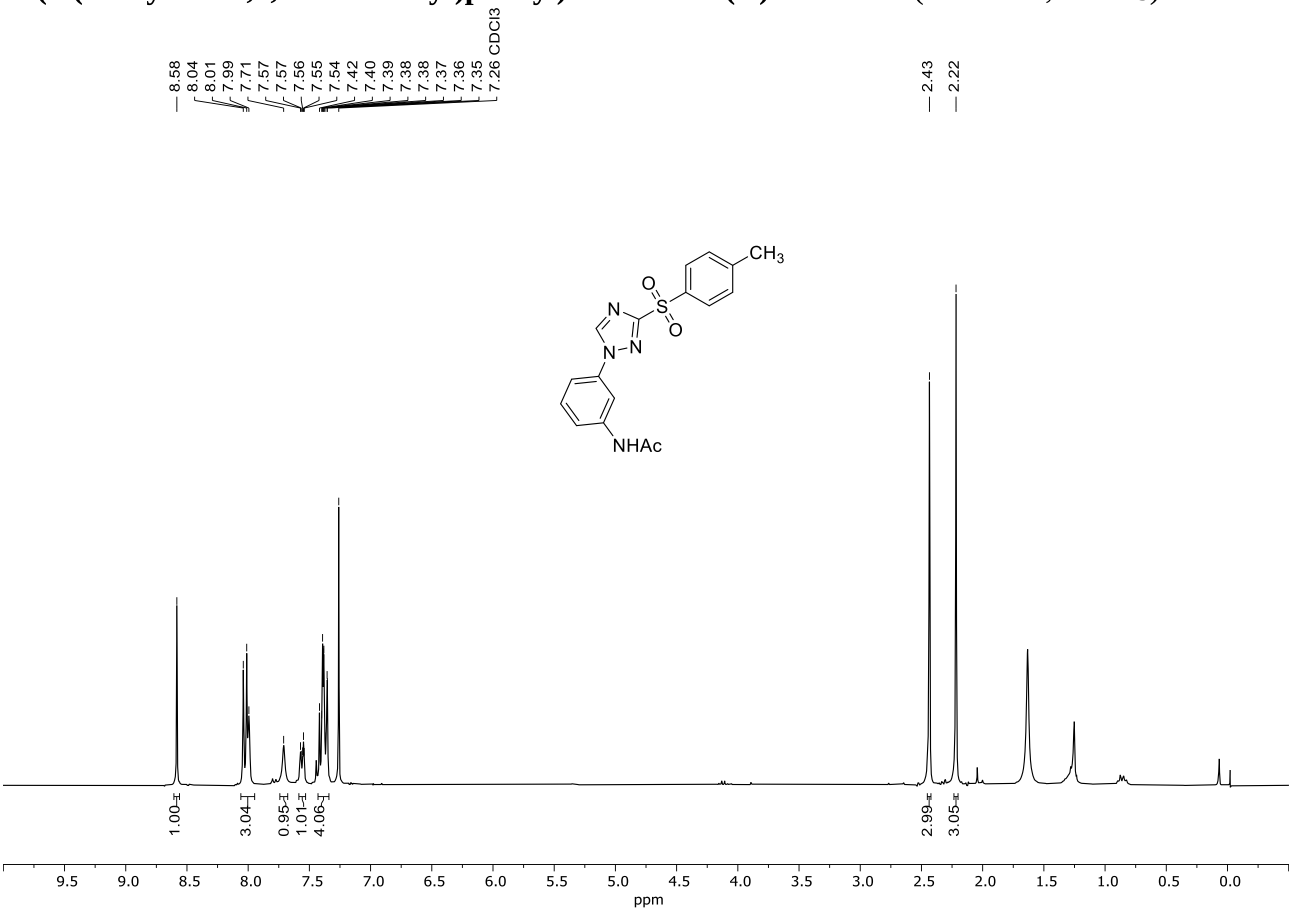

***N*-(3-(5-tosyl-1*H*-1,2,4-triazol-1-yl)phenyl)acetamide (3l).** $^{13}C\{^{1}H\}$ NMR (75 MHz, $CDCl_3$)

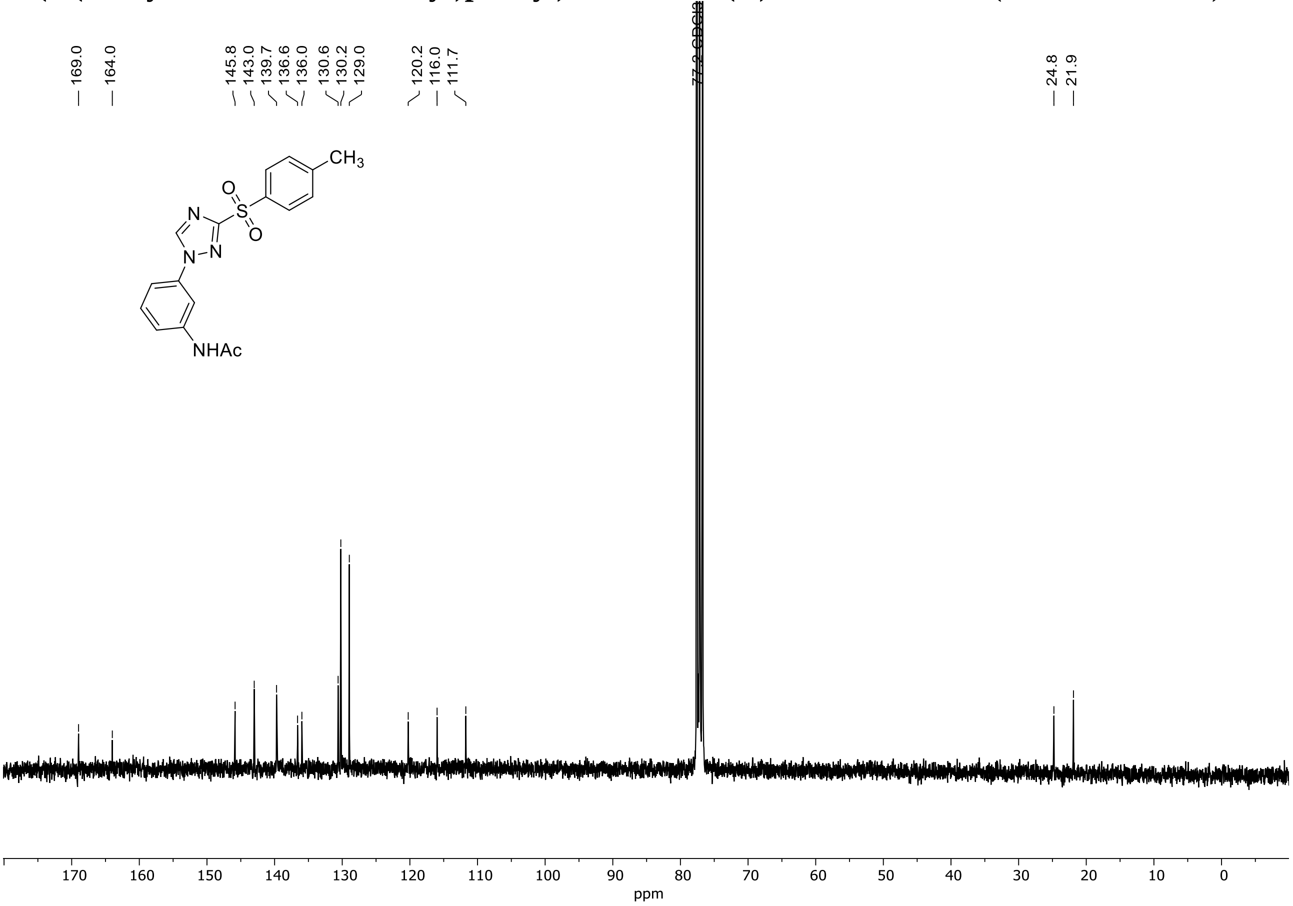

1-(2-(methylthio)phenyl)-5-tosyl-1*H*-1,2,4-triazole (3m). ^{1}H NMR (300 MHz, $CDCl_3$)

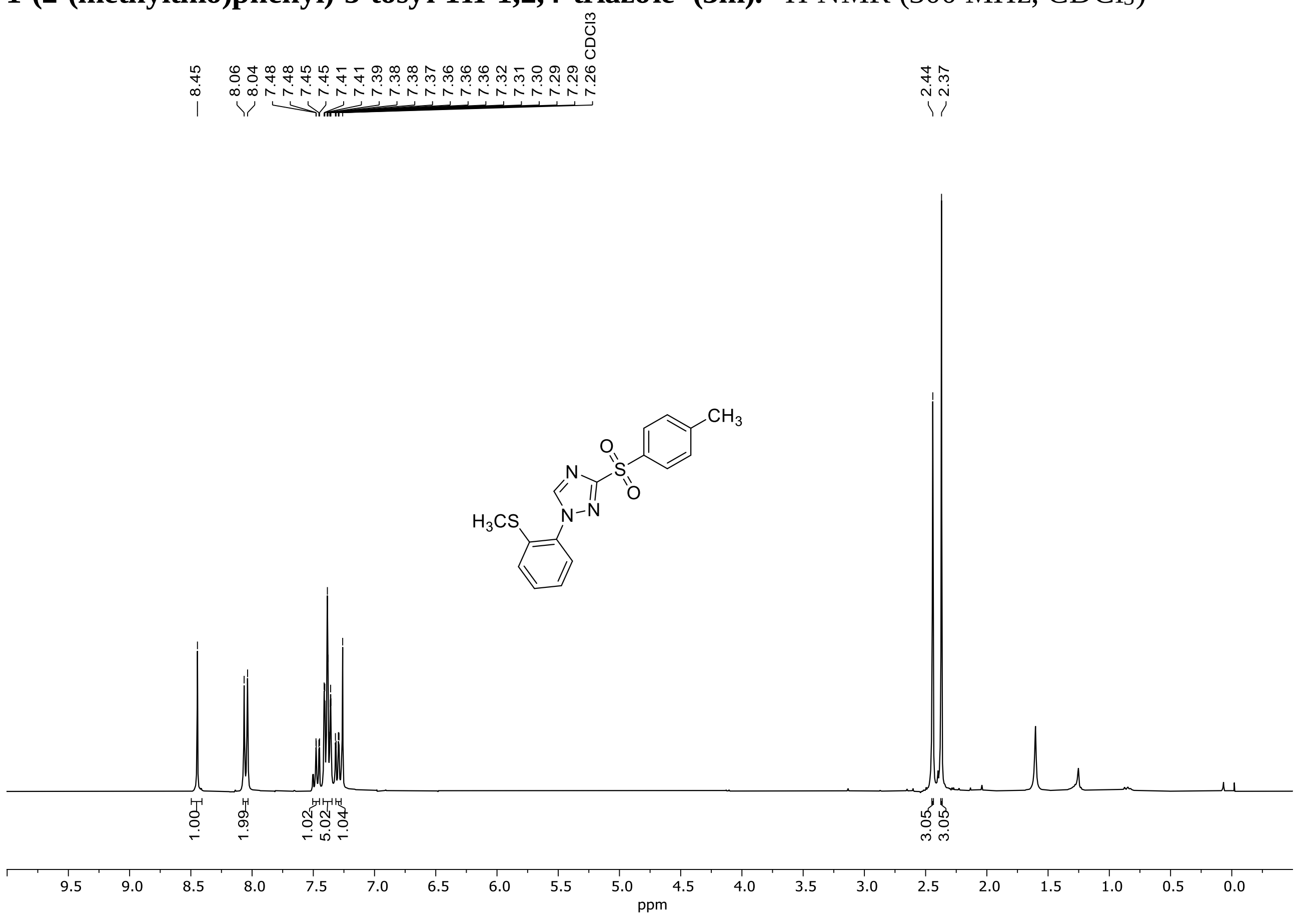

1-(2-(methylthio)phenyl)-5-tosyl-1*H*-1,2,4-triazole (3m). ^{13}C{^{1}H} NMR (75 MHz, $CDCl_3$)

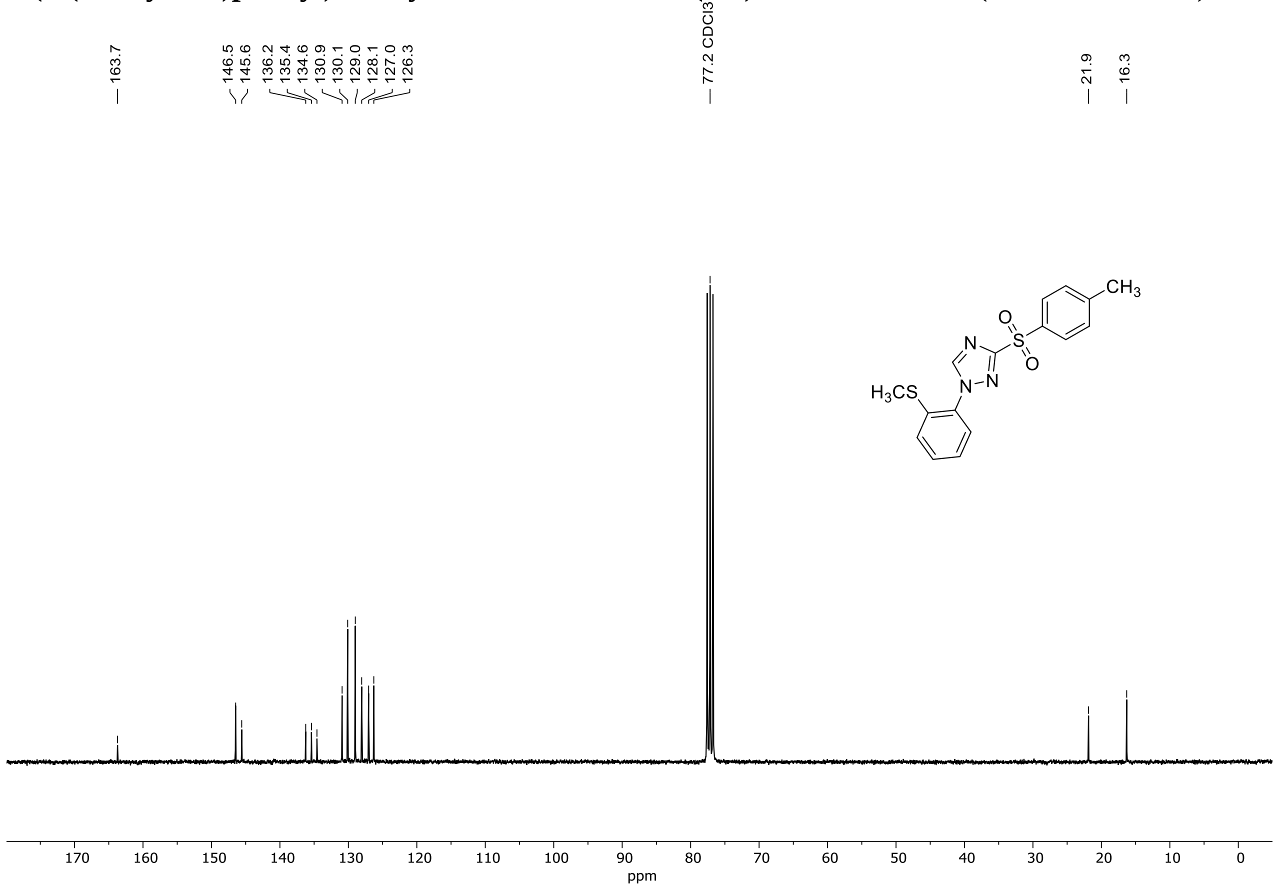

1-(2-chlorophenyl)-3-tosyl-1*H*-1,2,4-triazole (3n). ^{1}H NMR (300 MHz, $CDCl_3$)

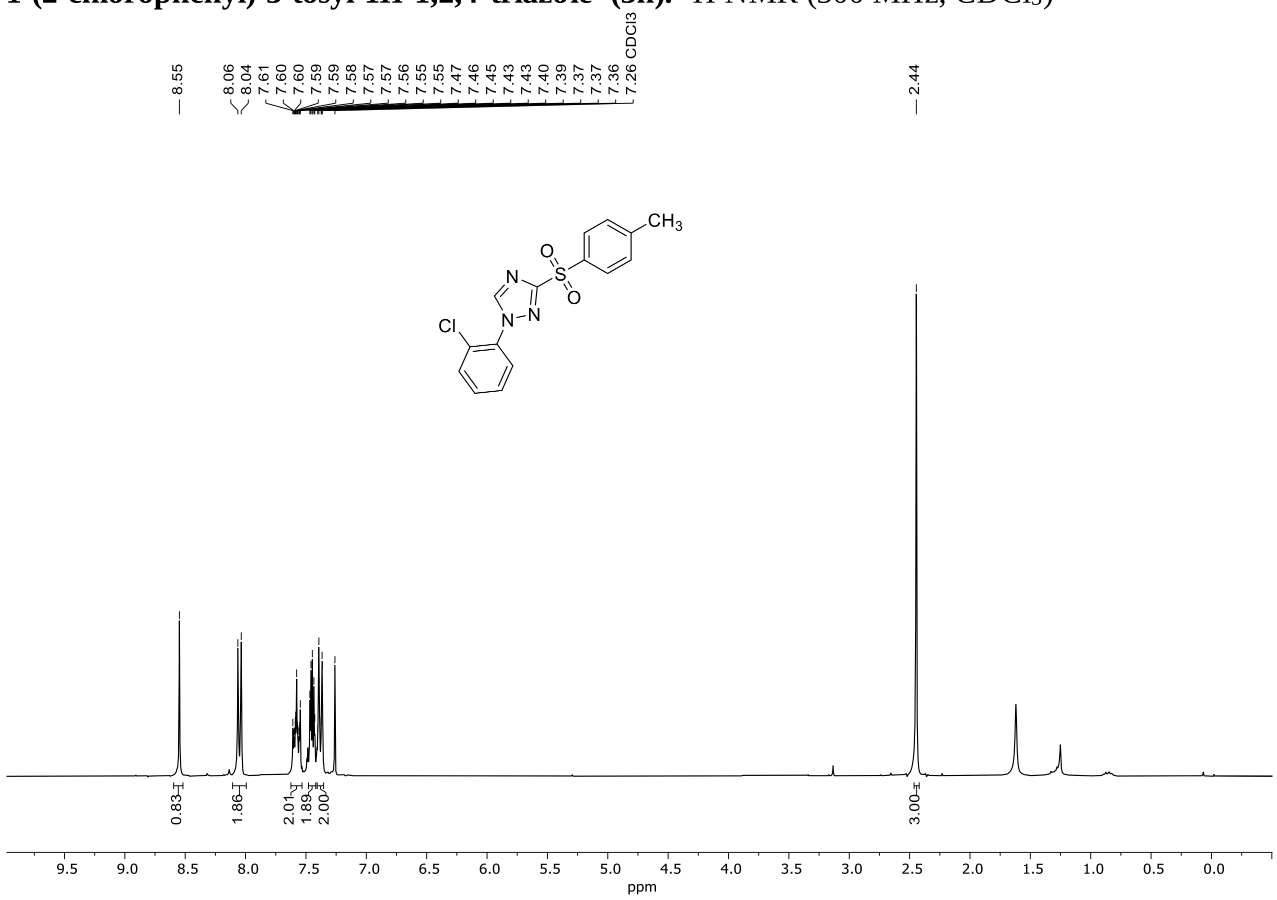

1-(2-chlorophenyl)-3-tosyl-1*H*-1,2,4-triazole (3n). ^{13}C{^{1}H} NMR (75 MHz, $CDCl_3$)

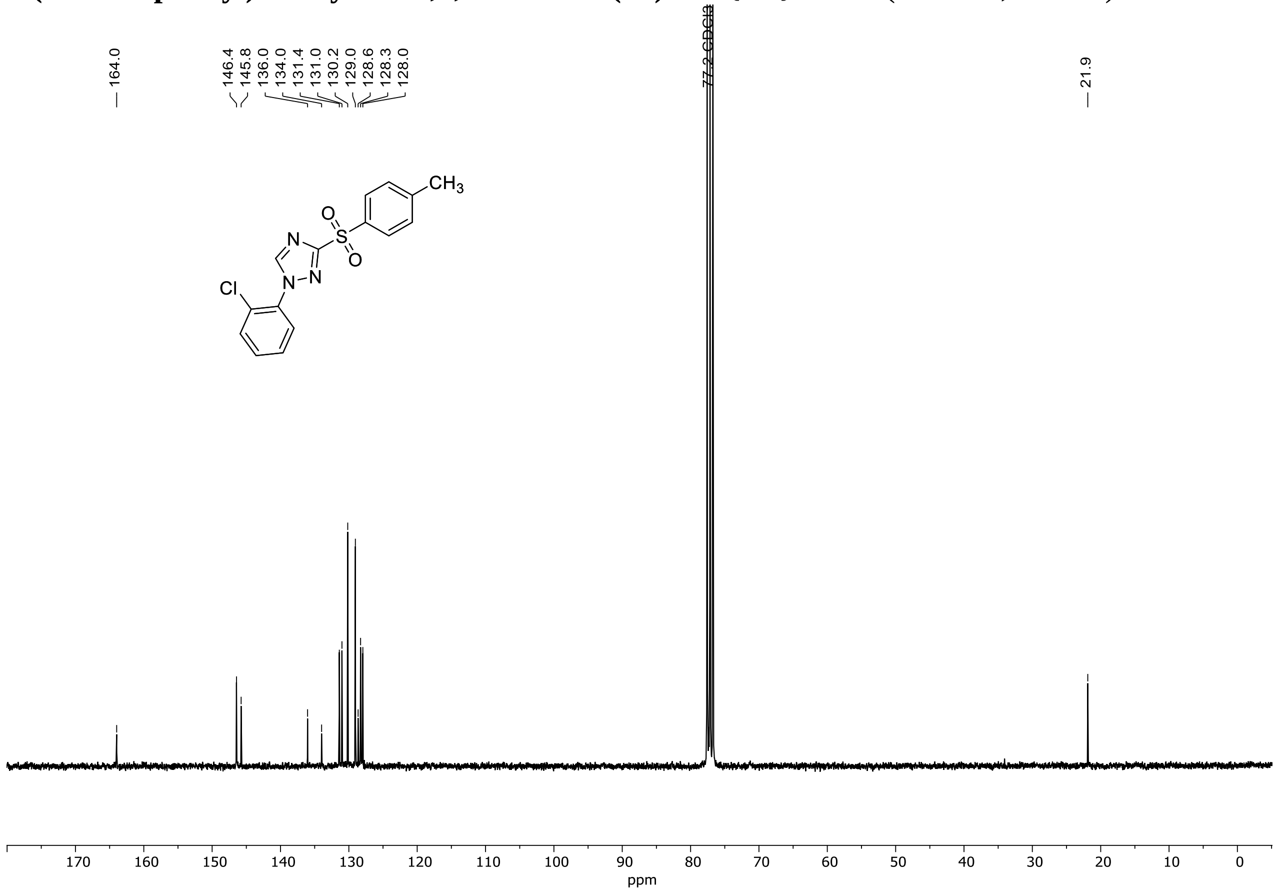

1-(3-chlorophenyl)-5-tosyl-1*H*-1,2,4-triazole (3o). ^{1}H NMR (300 MHz, $CDCl_3$)

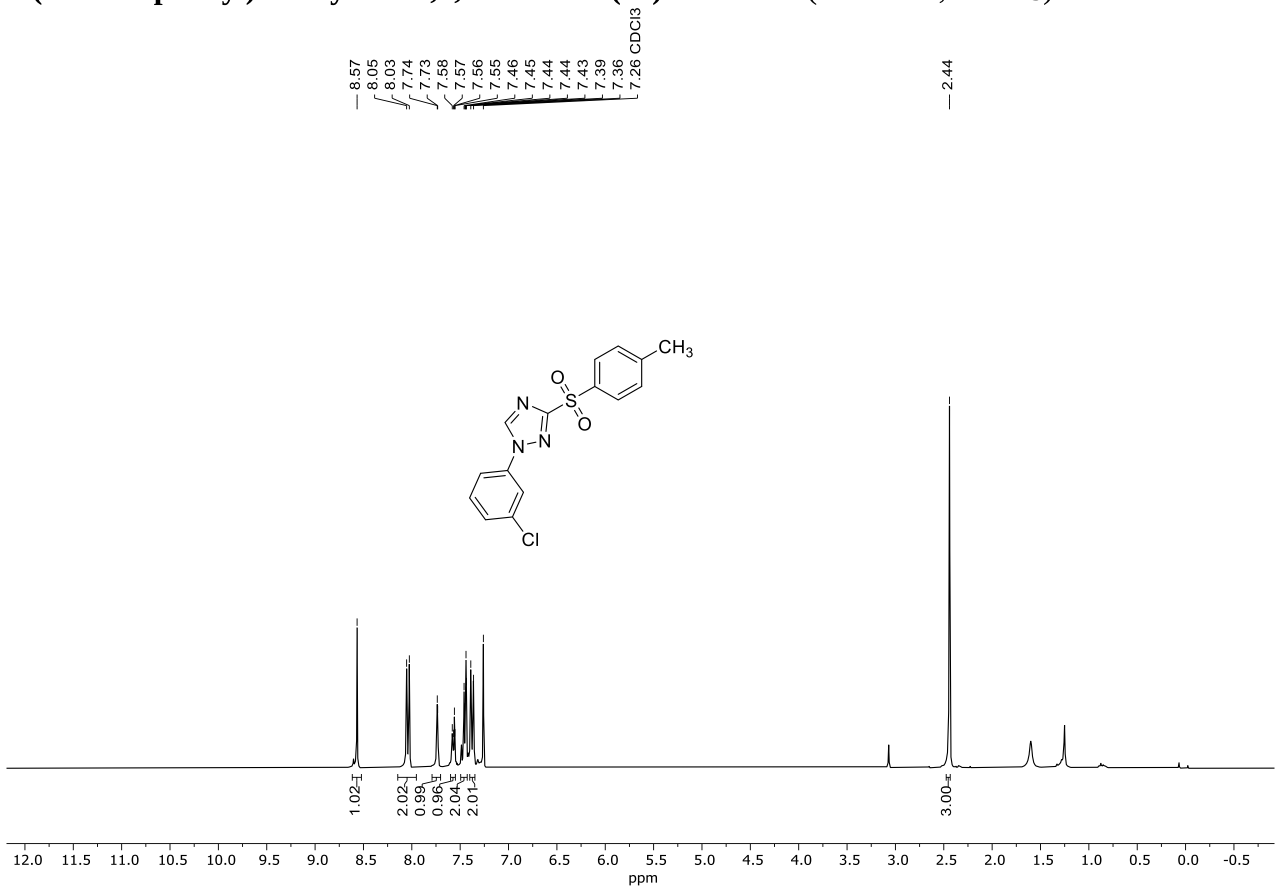

1-(3-chlorophenyl)-5-tosyl-1*H*-1,2,4-triazole (3o). $^{13}C\{^{1}H\}$ NMR (75 MHz, $CDCl_3$)

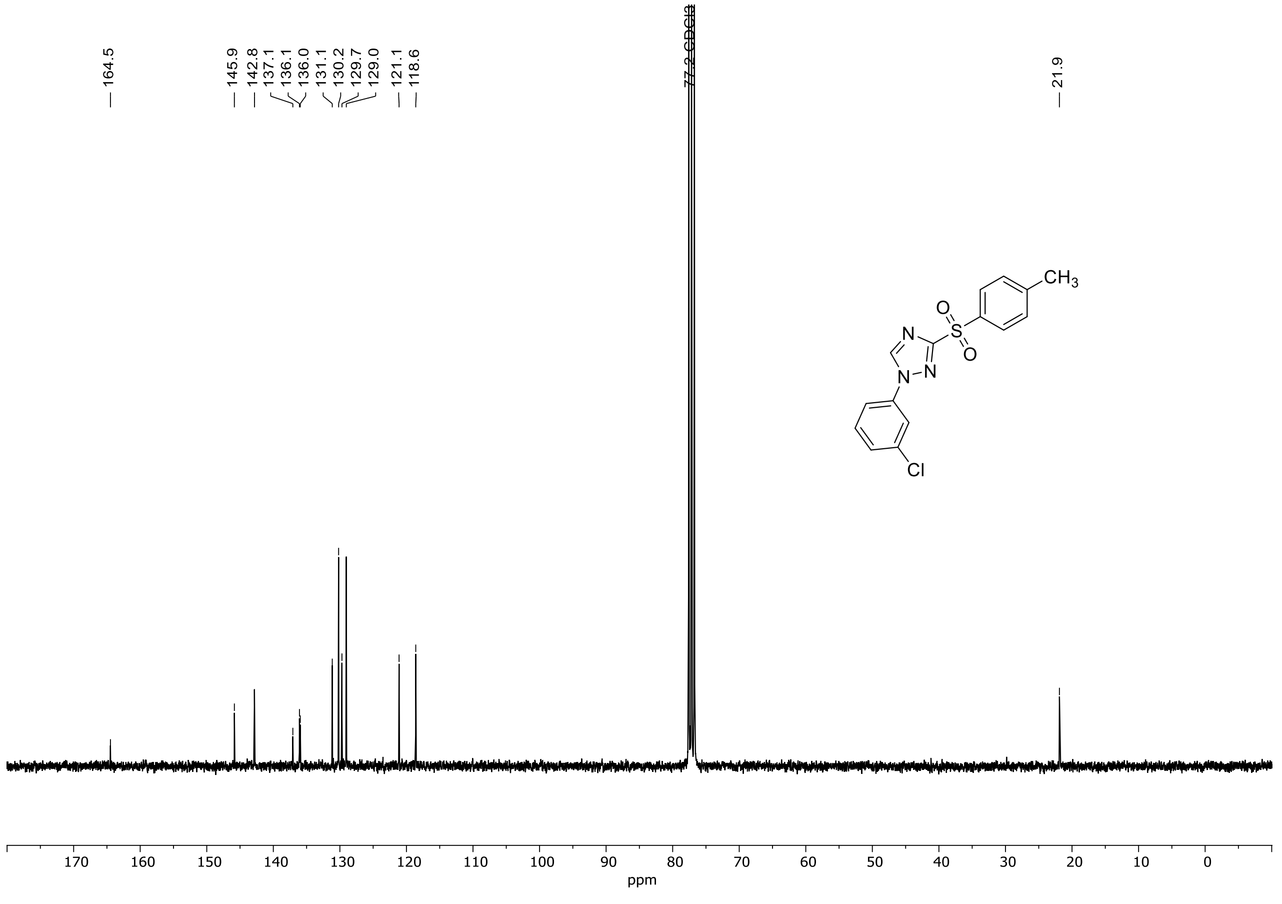

1-(4-chlorophenyl)-5-tosyl-1*H*-1,2,4-triazole (3p). ^{1}H NMR (300 MHz, $CDCl_3$)

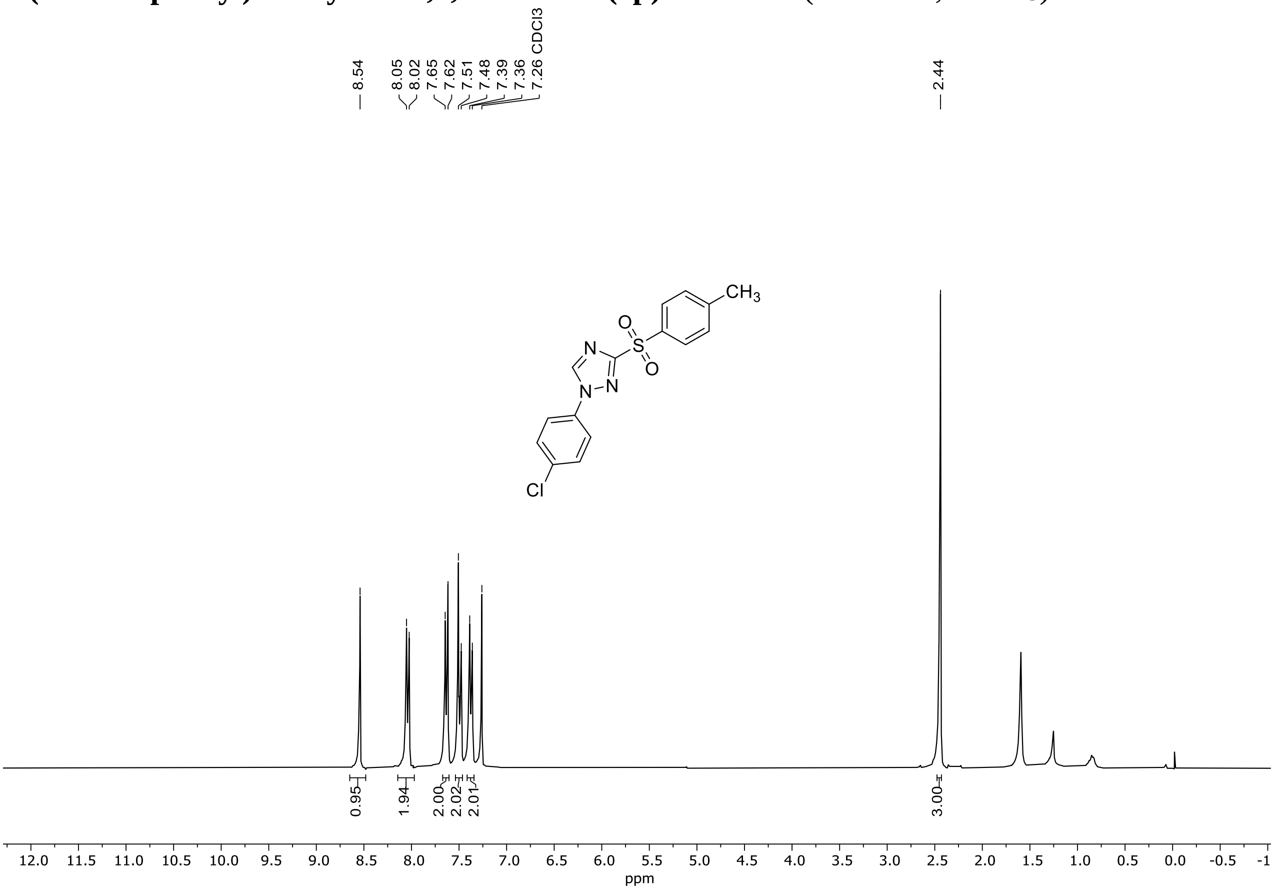

1-(4-chlorophenyl)-5-tosyl-1*H*-1,2,4-triazole (3p). $^{13}C\{^{1}H\}$ NMR (75 MHz, $CDCl_3$)

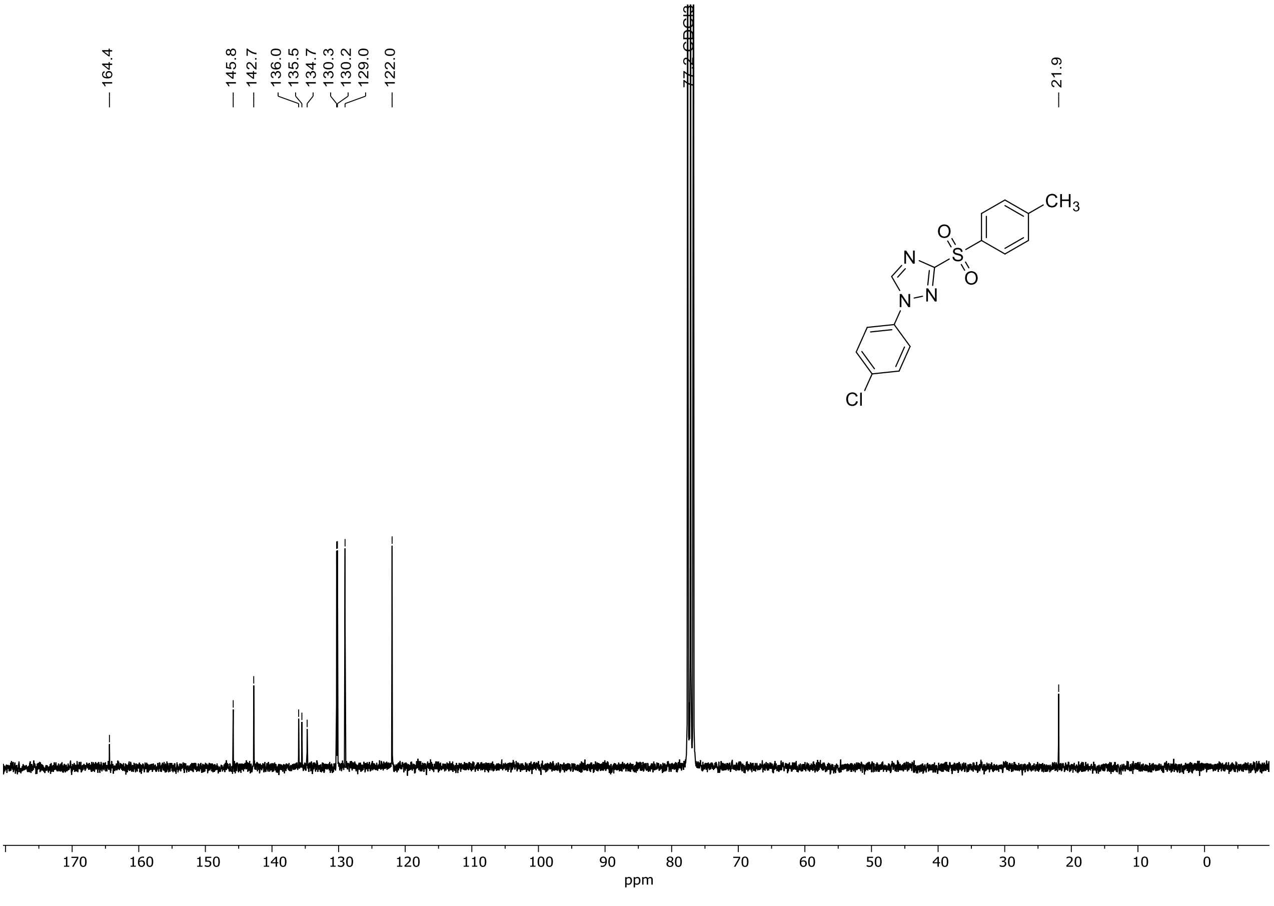

1-(4-bromophenyl)-5-tosyl-1*H*-1,2,4-triazole (3q). ^{1}H NMR (300 MHz, $CDCl_3$)

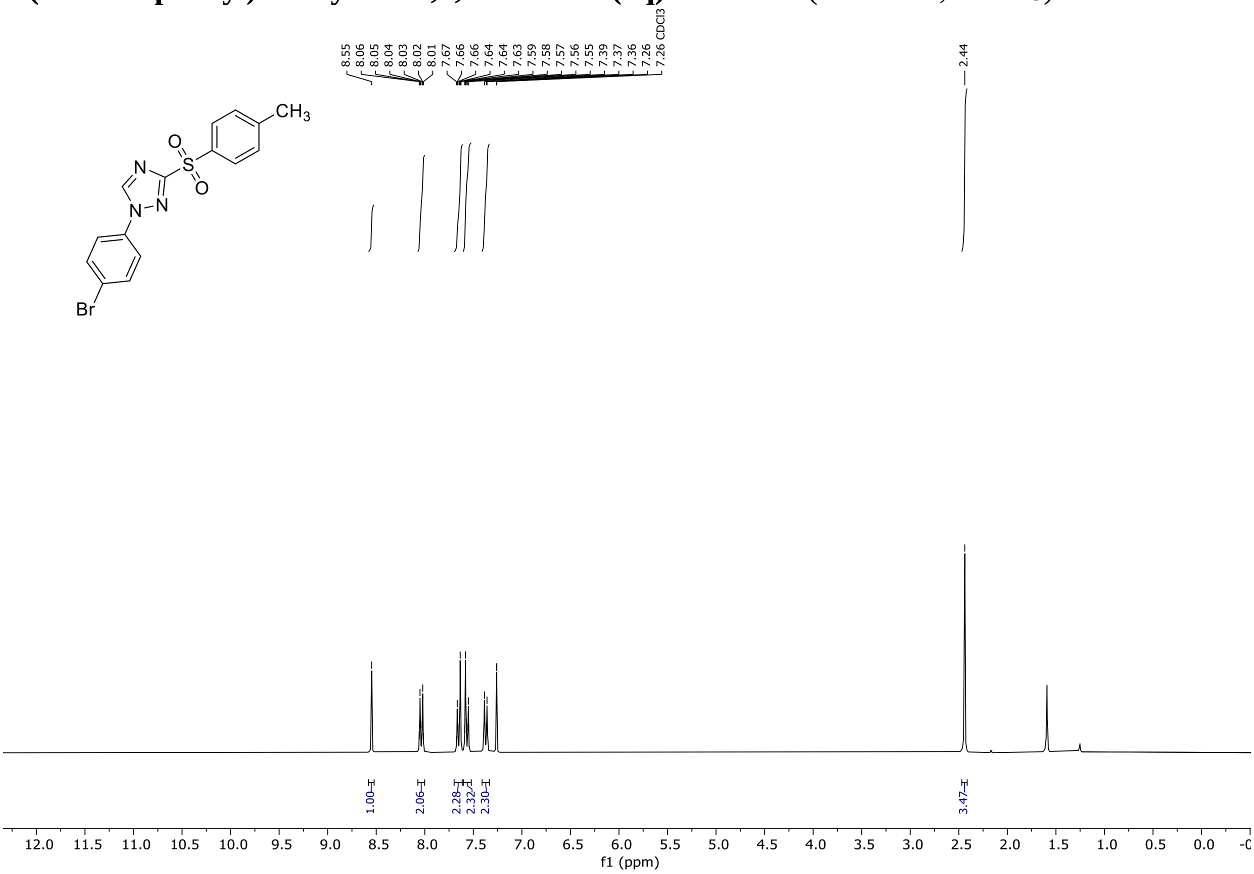

1-(4-bromophenyl)-5-tosyl-1*H*-1,2,4-triazole (3q). ^{13}C{^{1}H} NMR (75 MHz, $CDCl_3$)

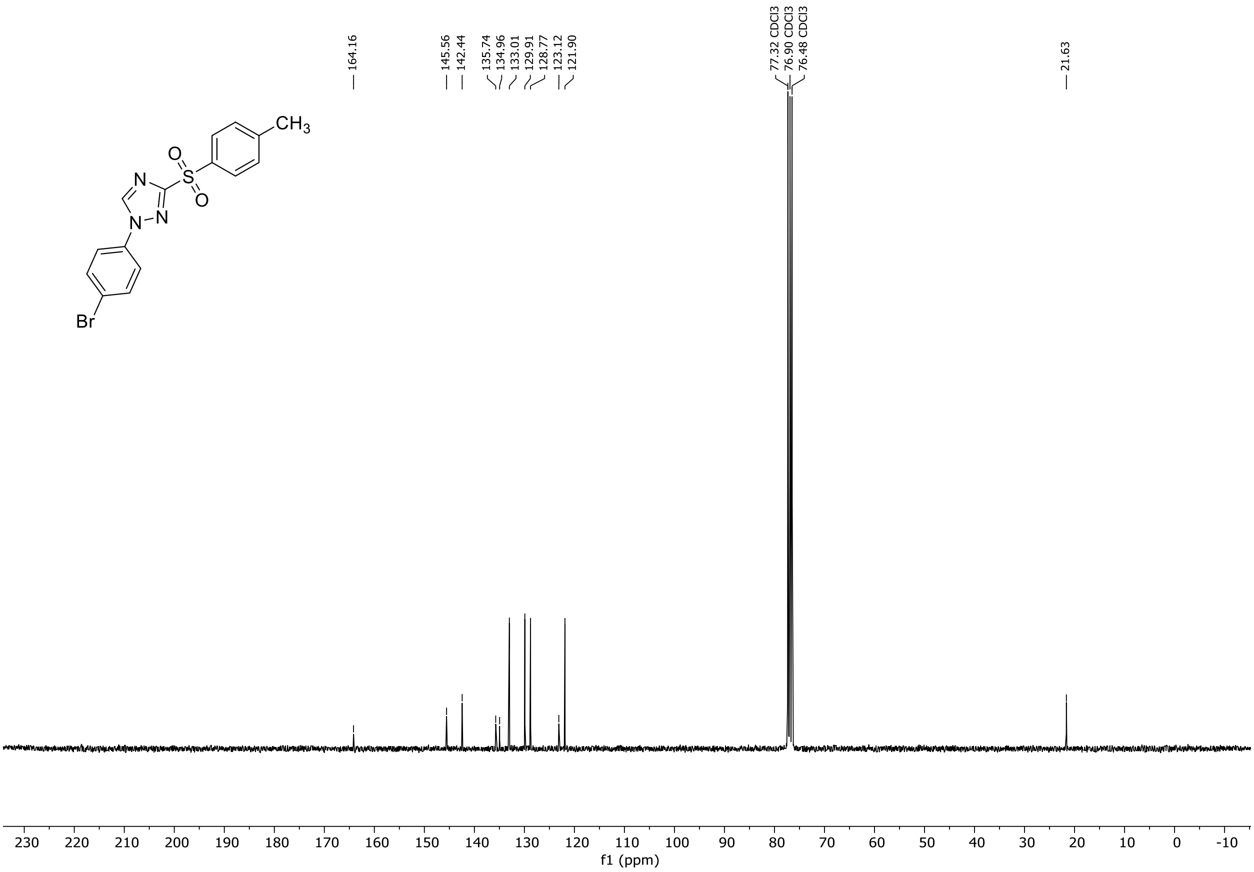

5-tosyl-1-(4-(trifluoromethoxy)phenyl)-1*H*-1,2,4-triazole (3r). ^{1}H NMR (300 MHz, $CDCl_3$)

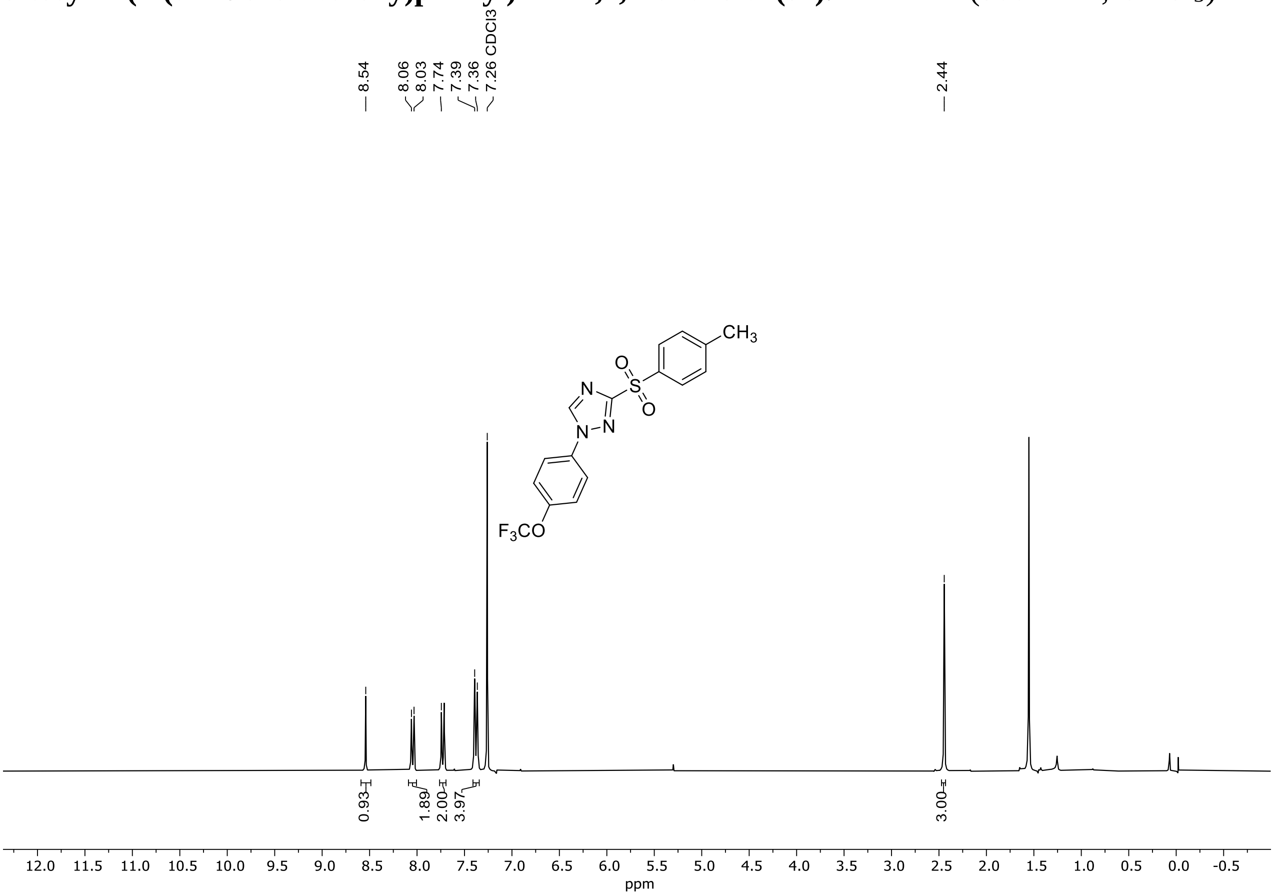

5-tosyl-1-(4-(trifluoromethoxy)phenyl)-1*H*-1,2,4-triazole (3r). $^{13}C\{^{1}H\}$ NMR (75 MHz, $CDCl_3$)

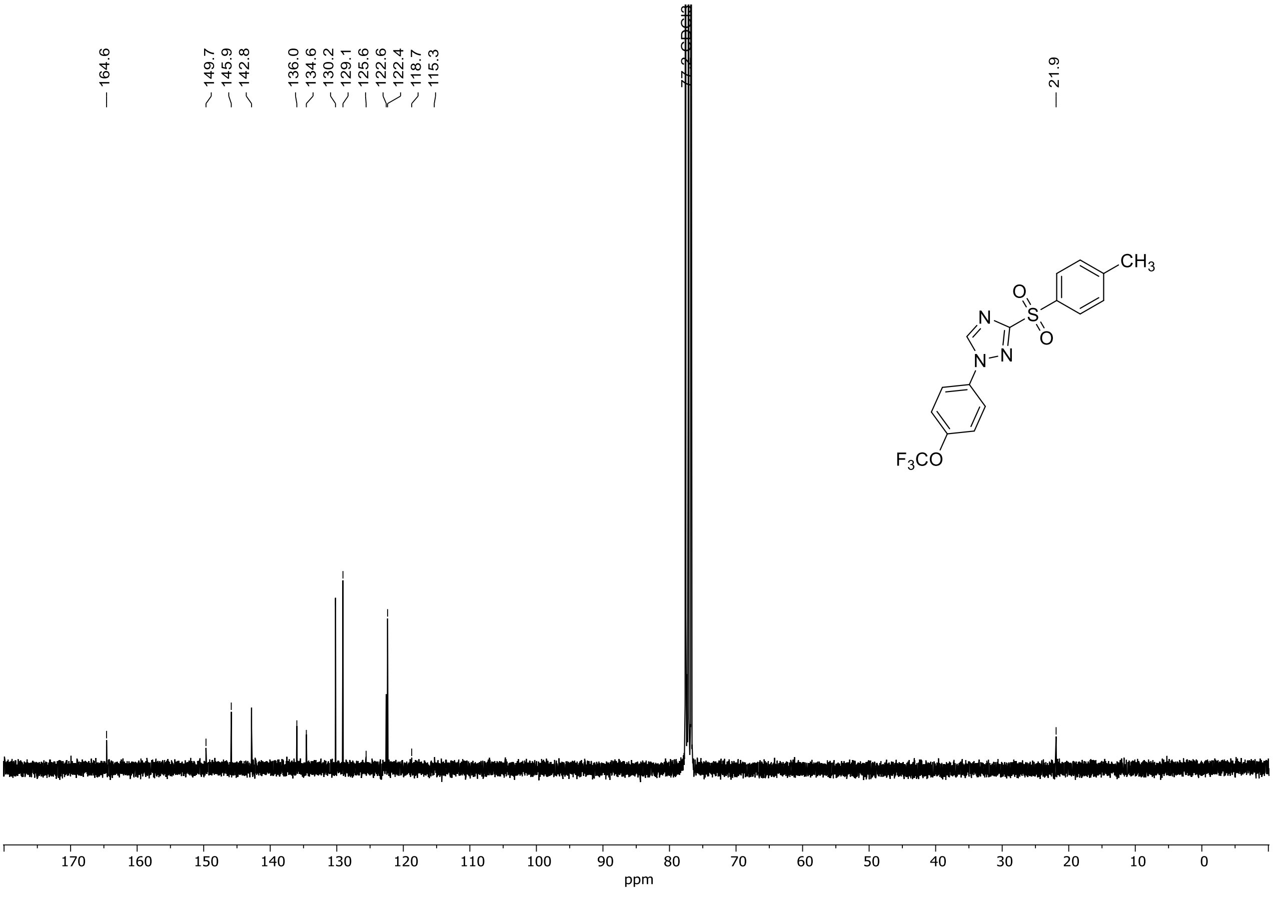

5-tosyl-1-(4-(trifluoromethoxy)phenyl)-1*H*-1,2,4-triazole (3r). $^{19}F\{^1H\}$ NMR (376 MHz, $CDCl_3$)

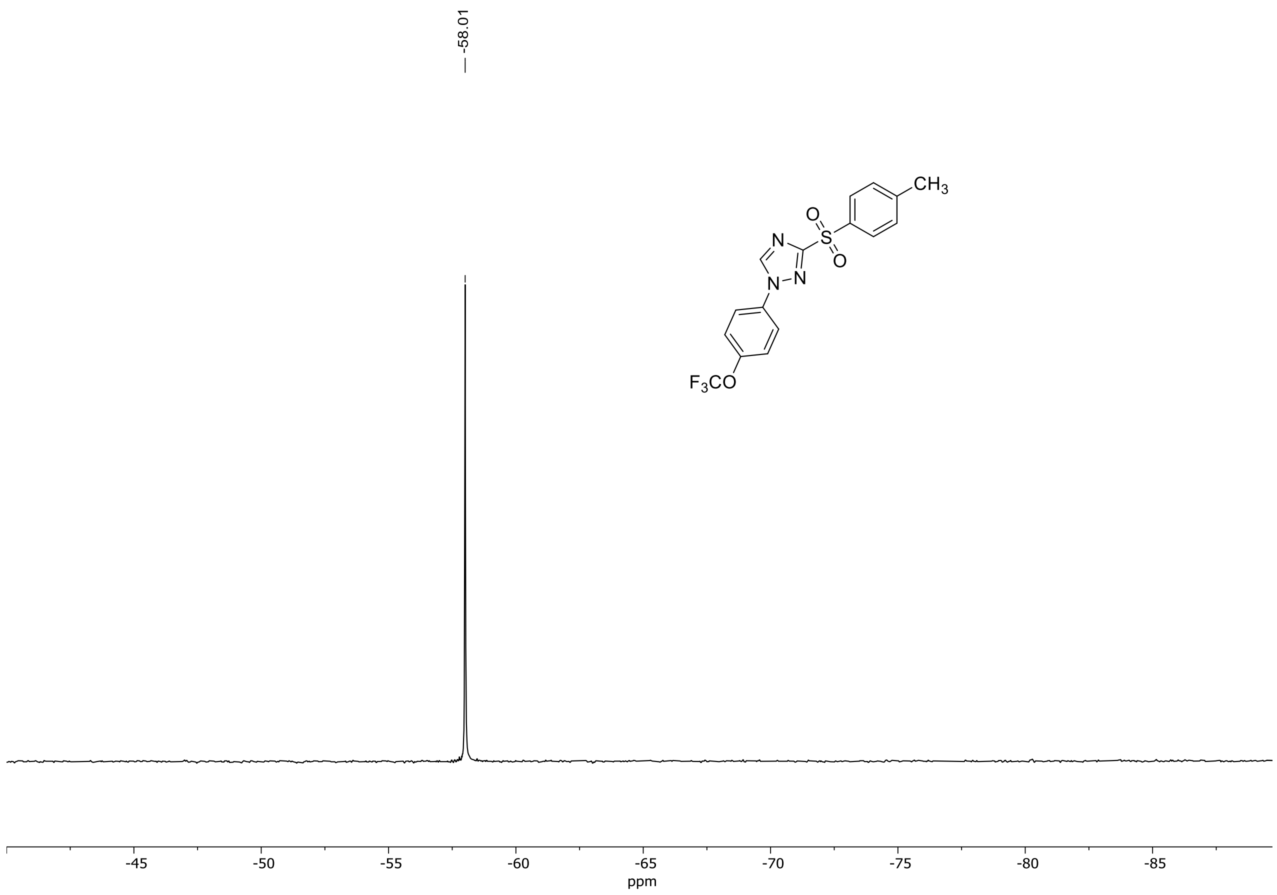

1-phenyl-5-tosyl-1*H*-1,2,4-triazole (3s). ^{1}H NMR (300 MHz, $CDCl_3$)

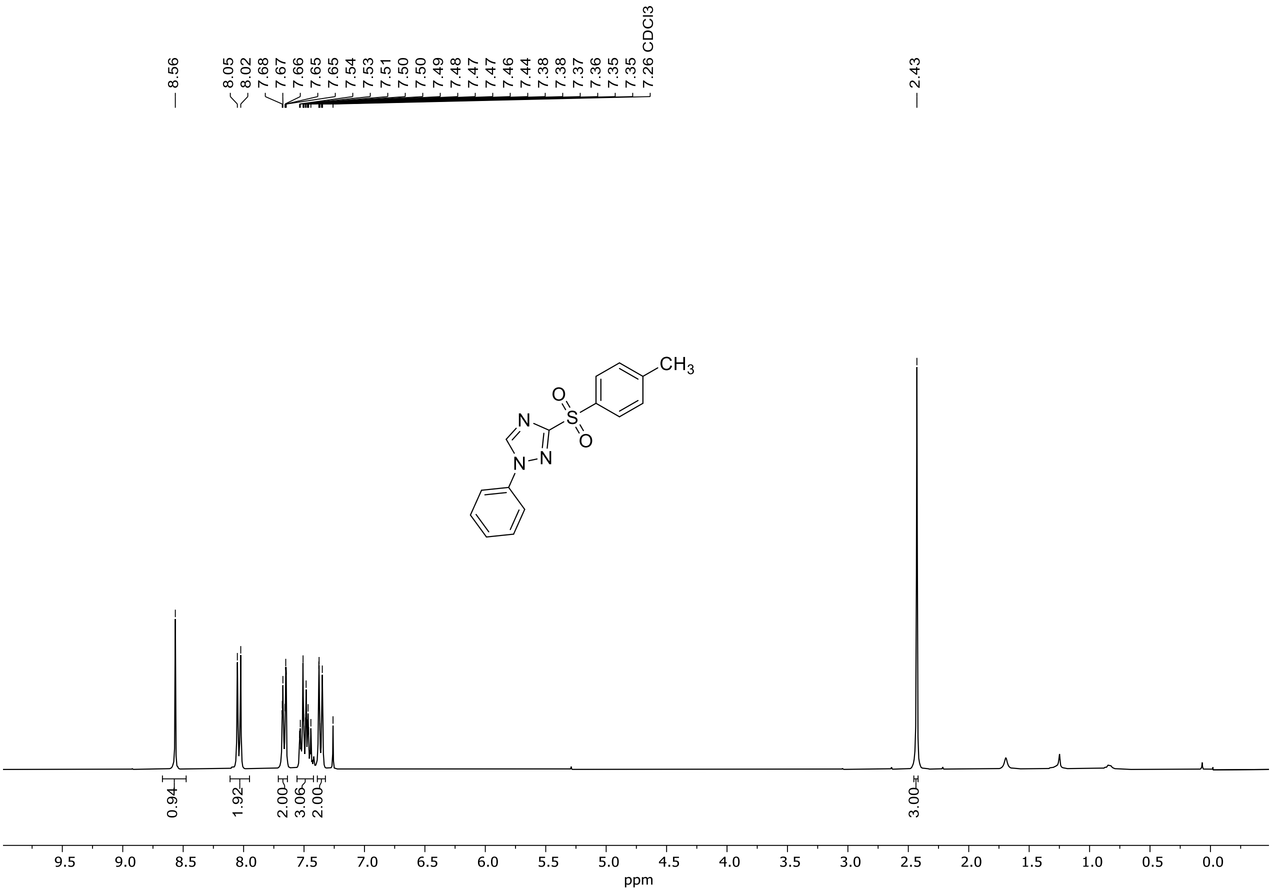

1-phenyl-5-tosyl-1*H*-1,2,4-triazole (3s). ^{13}C{^{1}H} NMR (75 MHz, $CDCl_3$)

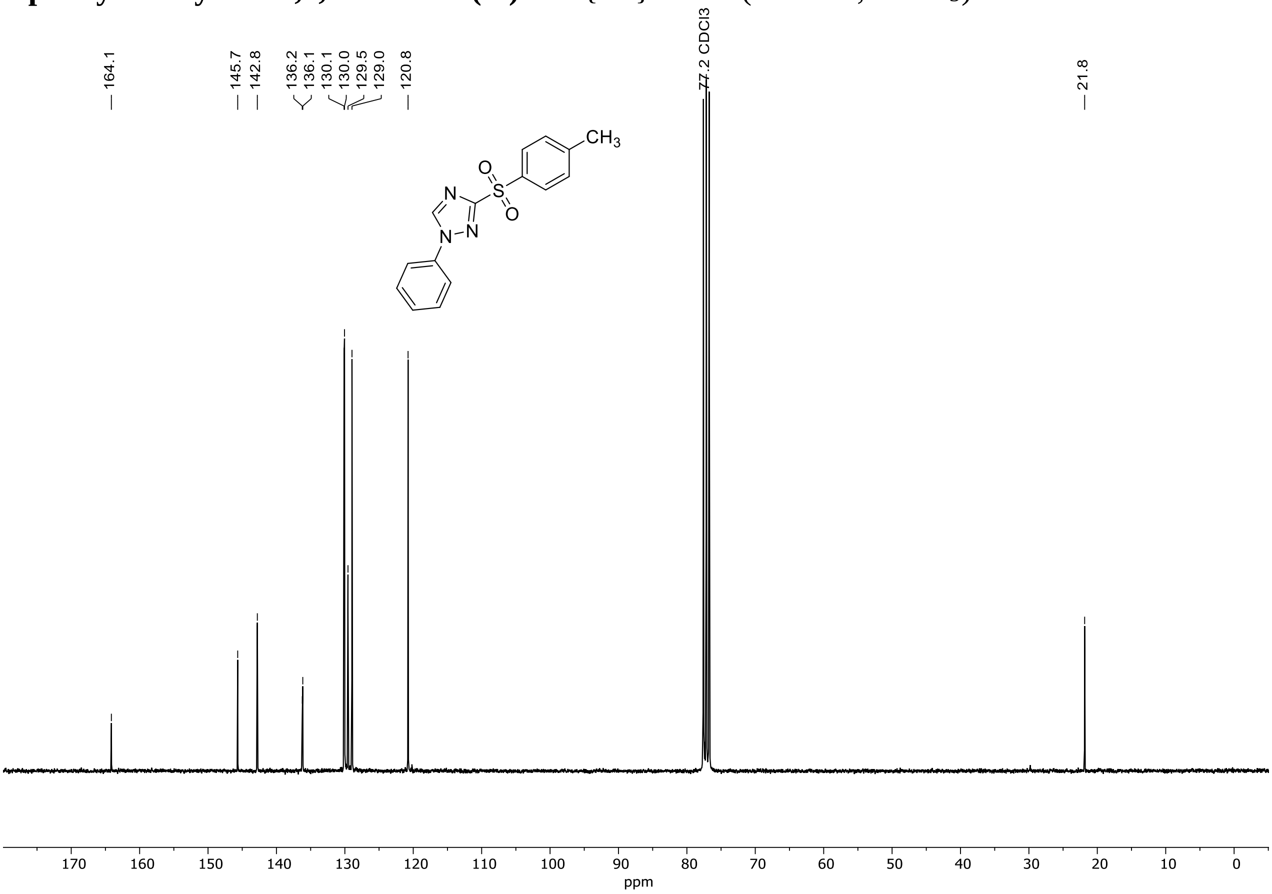

1-(p-tolyl)-3-tosyl-1H-1,2,4-triazole (3t). ^{1}H NMR (300 MHz, $CDCl_3$)

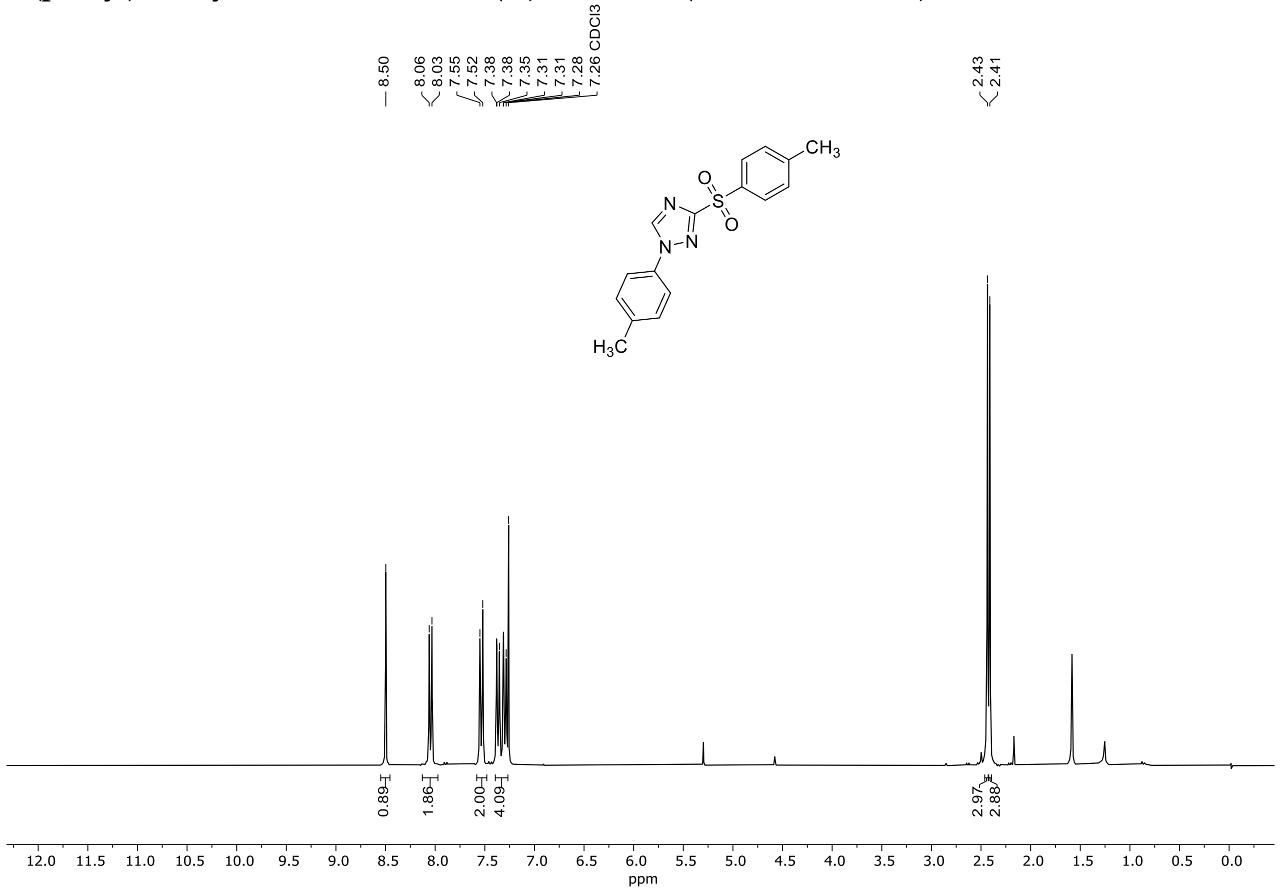

1-(p-tolyl)-3-tosyl-1H-1,2,4-triazole (3t). $^{13}C\{^{1}H\}$ NMR (75 MHz, $CDCl_3$)

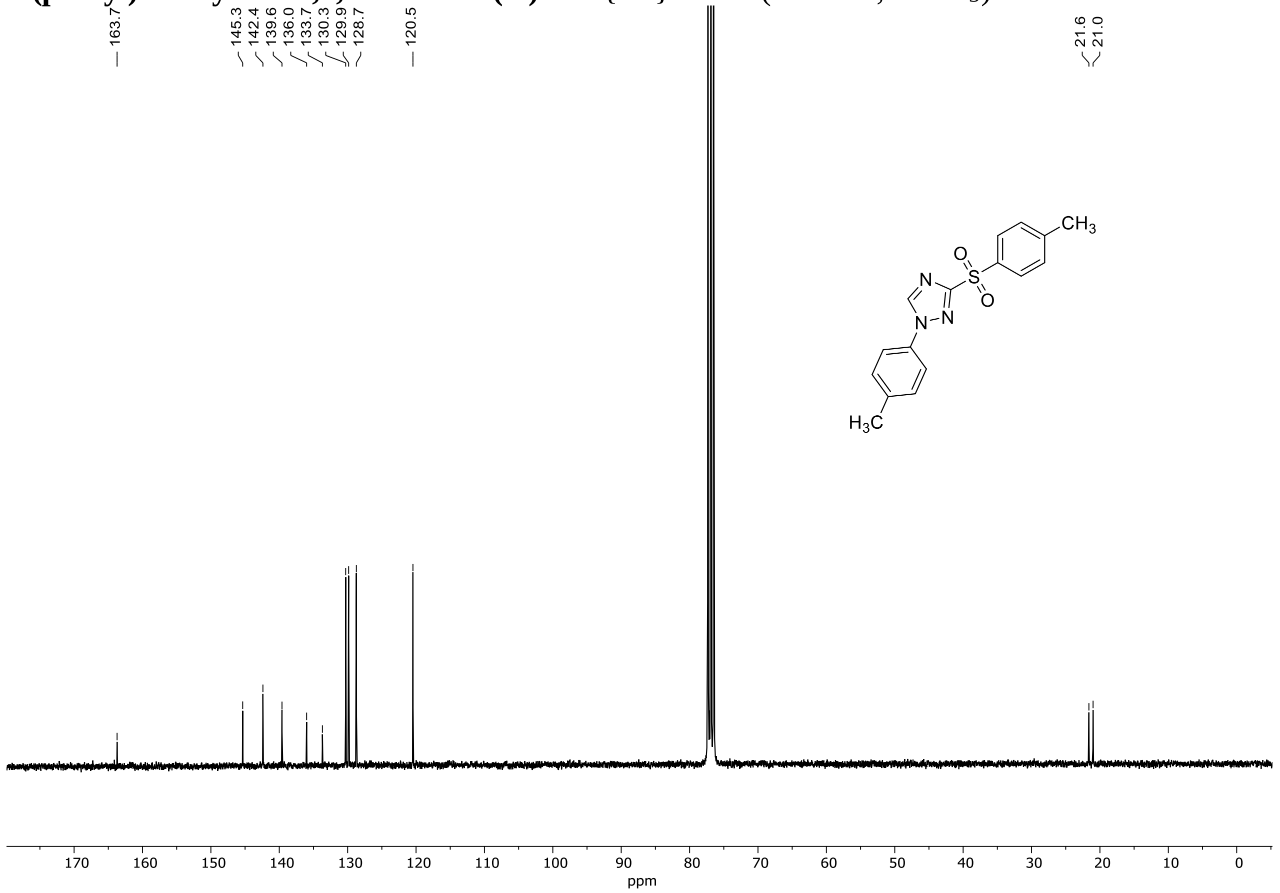

5-tosyl-1-(3,4,5-trimethoxyphenyl)-*1H*-1,2,4-triazole (3u). ^{1}H NMR (300 MHz, $CDCl_3$)

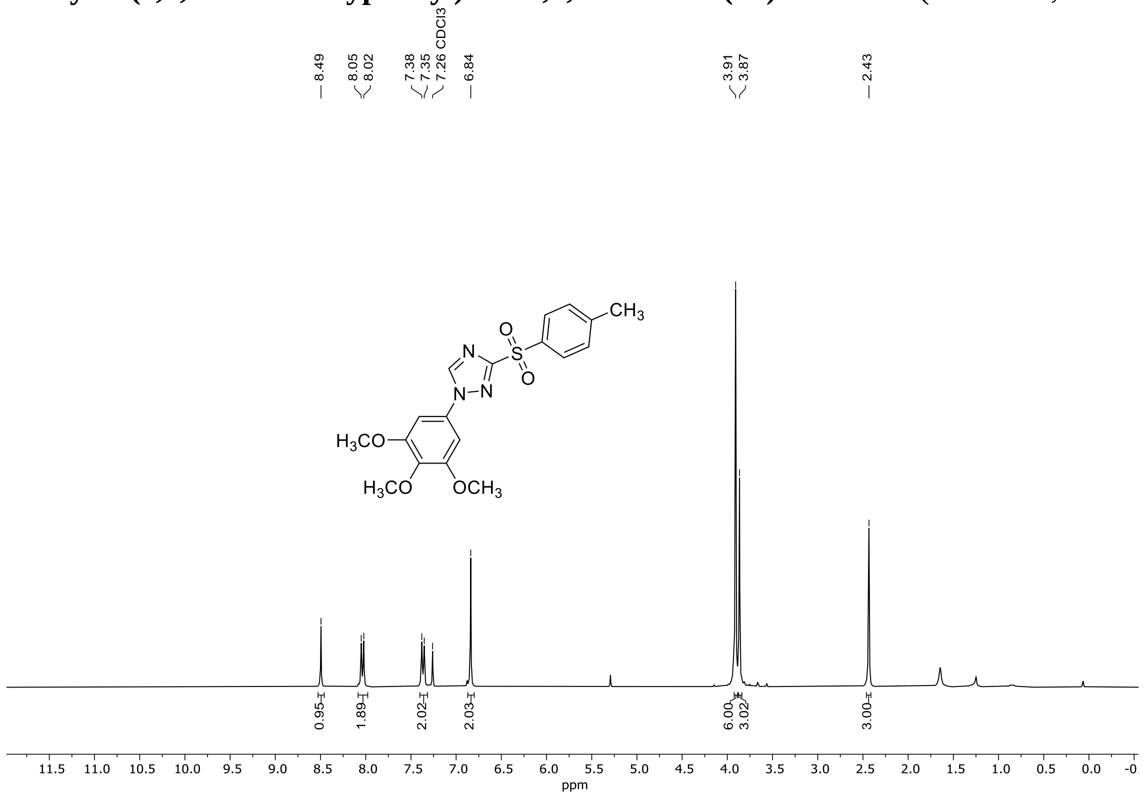

5-tosyl-1-(3,4,5-trimethoxyphenyl)-*1H*-1,2,4-triazole (3u). $^{13}C\{^{1}H\}$ NMR (75 MHz, $CDCl_3$)

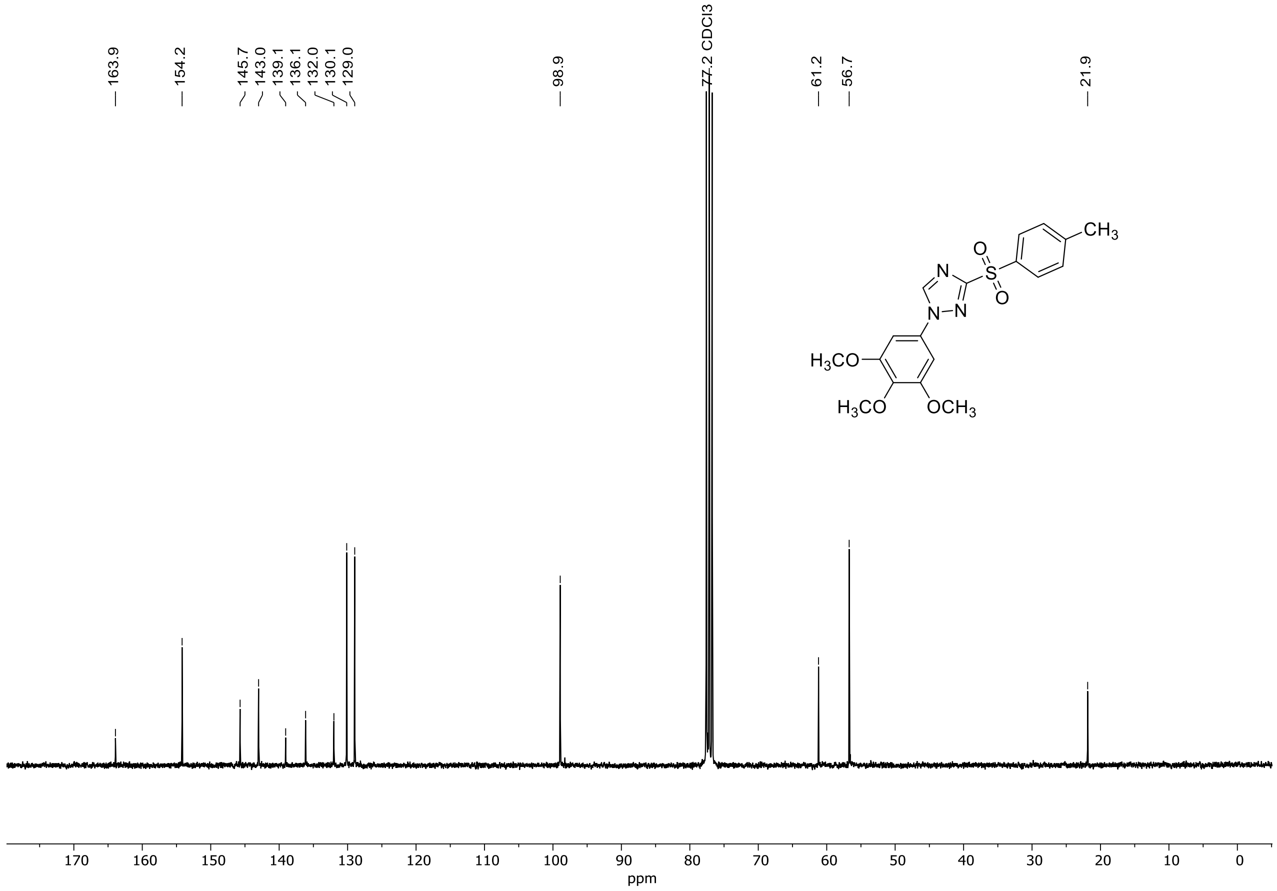

1-(4-(1*H*-1,2,4-triazol-1-yl)phenyl)ethan-1-one (3v). ^{1}H NMR (400 MHz, $CDCl_3$)

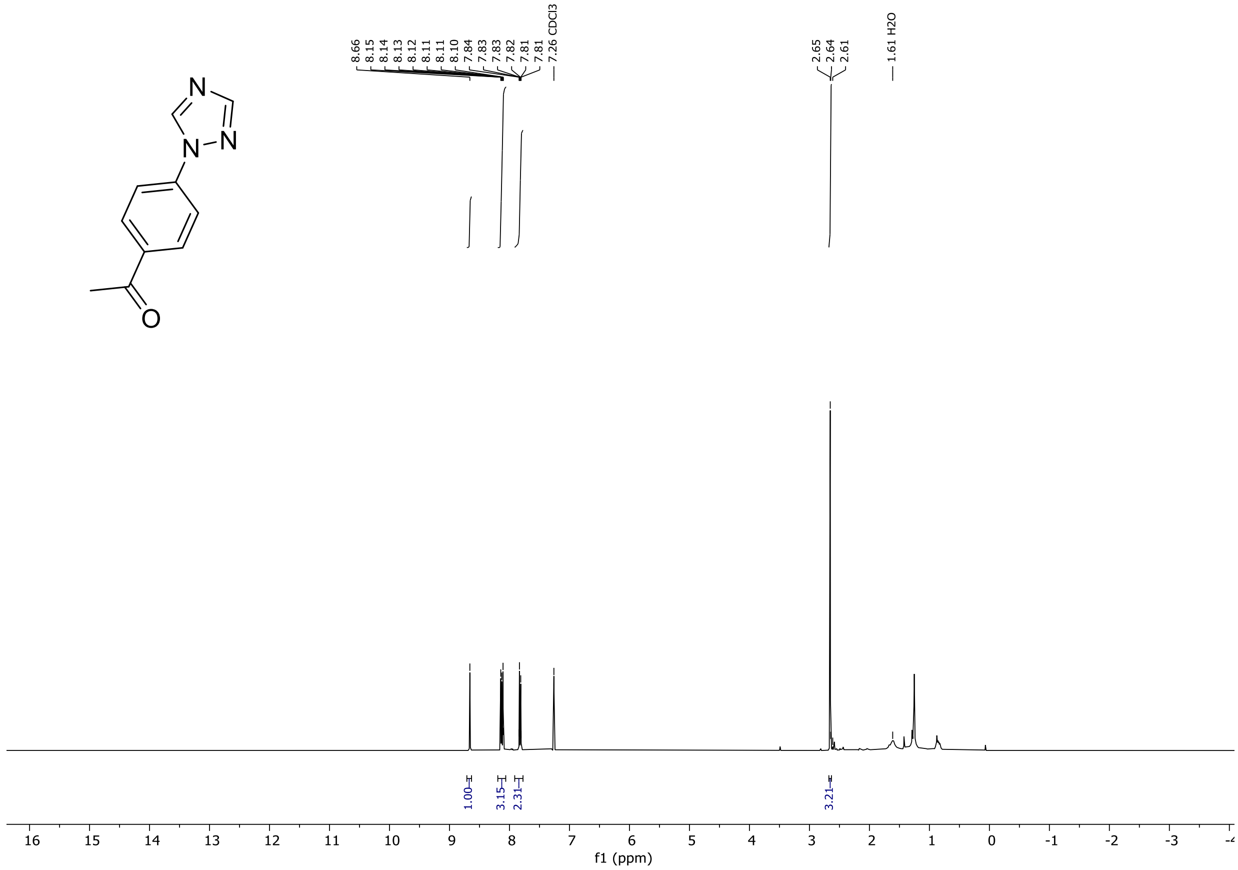

1-(4-(1*H*-1,2,4-triazol-1-yl)phenyl)ethan-1-one (3v). ^{13}C{^{1}H} NMR (101 MHz, $CDCl_3$)

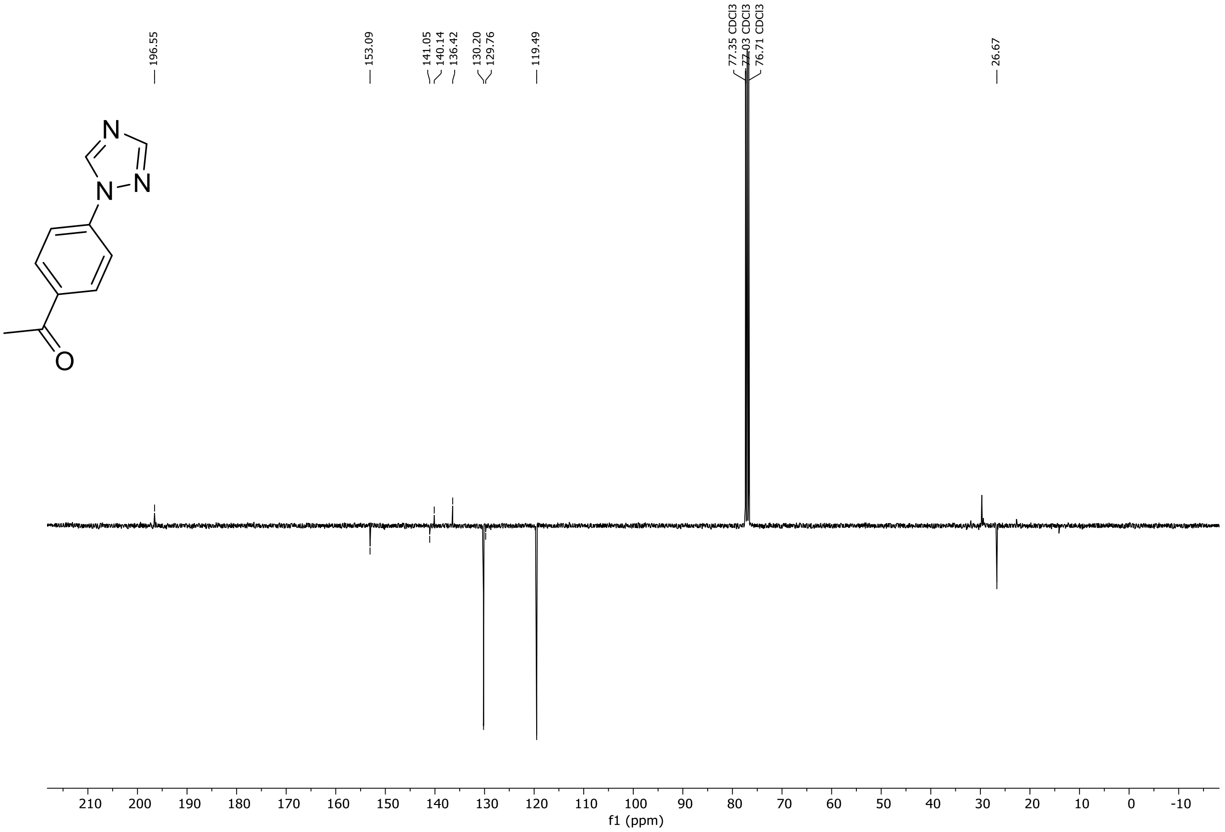

1-(4-nitrophenyl)-1*H*-1,2,4-triazole (3w). ^{1}H NMR (400 MHz, $CDCl_3$)

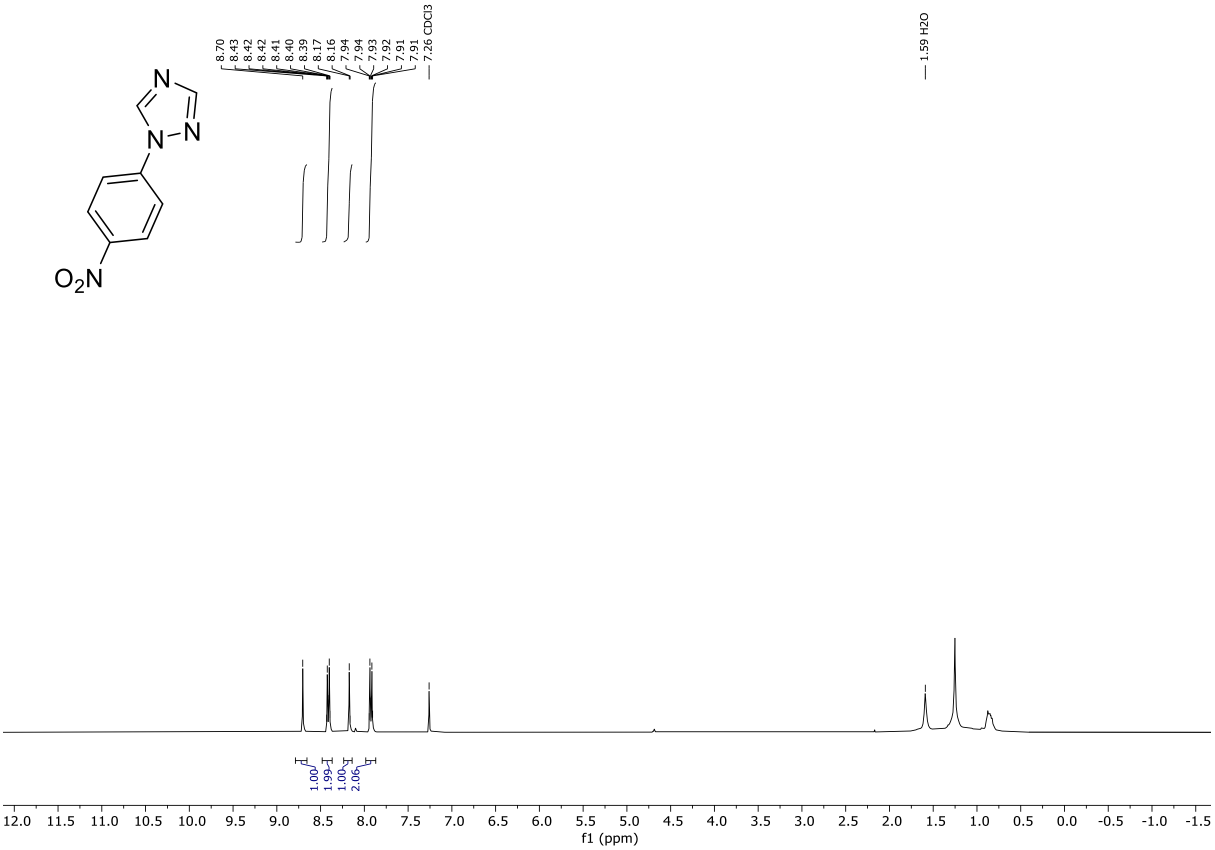

1-(4-nitrophenyl)-1*H*-1,2,4-triazole (3w). $^{13}C\{^{1}H\}$ NMR (101 MHz, $CDCl_3$)

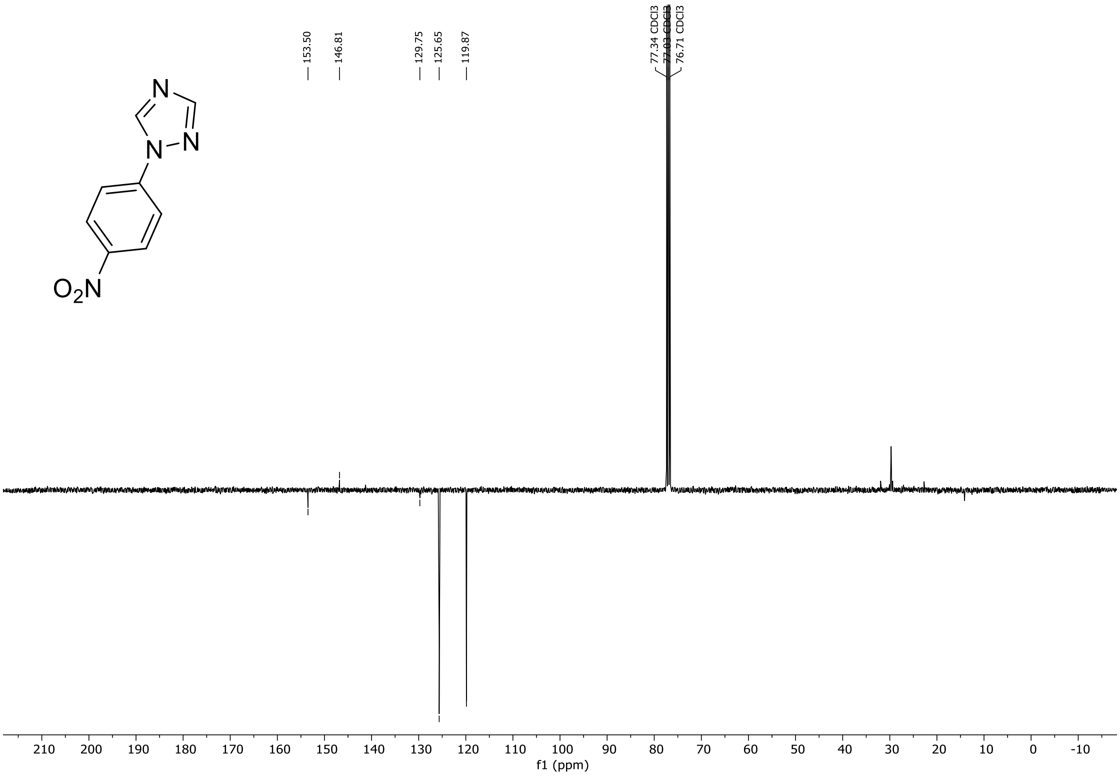

1-(4-nitrophenyl)-3-tosyl-1*H*-1,2,4-triazole (3x). ^{1}H NMR (400 MHz, $CDCl_3$)

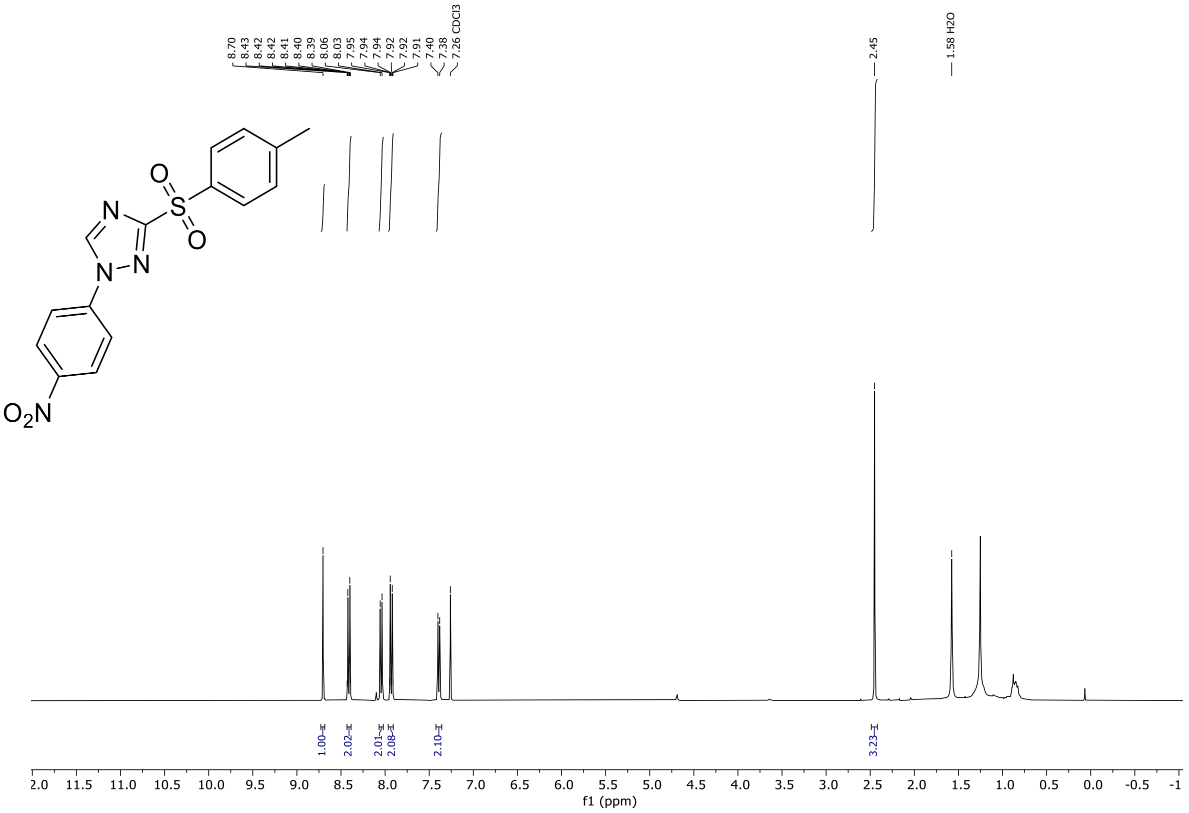

1-(4-nitrophenyl)-3-tosyl-1*H*-1,2,4-triazole (3x). ^{13}C{^{1}H} NMR (101 MHz, $CDCl_3$)

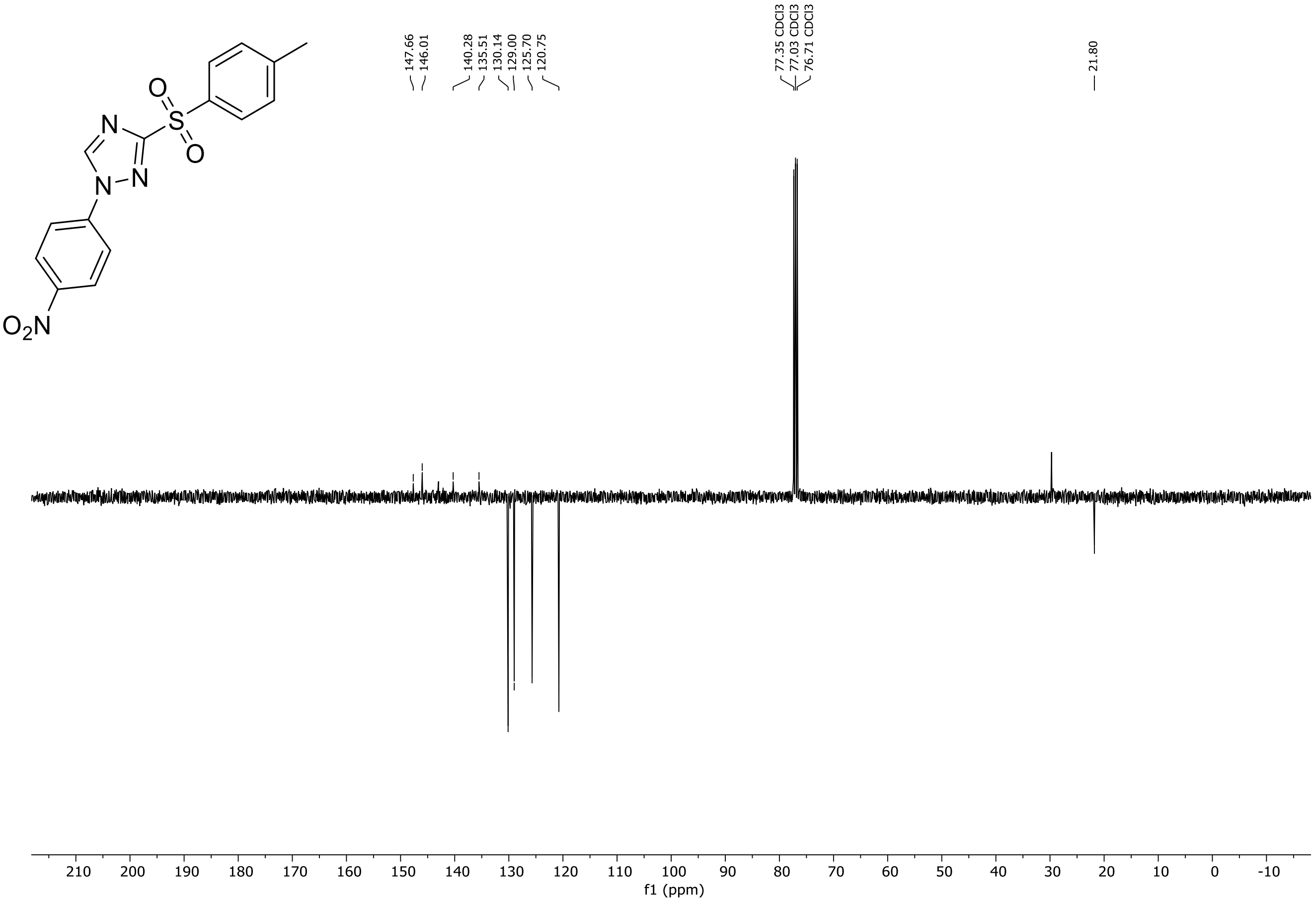

Supplement: Supplementary file 1 [file gg5c00080_si_001.pdf]
